# Enabling Controlling Complex Networks with Local Topological Information

**DOI:** 10.1038/s41598-018-22655-5

**Published:** 2018-03-15

**Authors:** Guoqi Li, Lei Deng, Gaoxi Xiao, Pei Tang, Changyun Wen, Wuhua Hu, Jing Pei, Luping Shi, H. Eugene Stanley

**Affiliations:** 1 Center for Brain Inspired Computing Research, Department of Precision Instrument, Tsinghua University, Beijing, P. R. China; 2 School of Electrical and Electronic Engineering, Nanyang Technological University, Singapore, Singapore; 3 Center for Polymer Studies, Department of Physics, Boston University, Boston, USA; 4 Beijing Innovation Center for Future Chip, Tsinghua University, Beijing, P. R. China; 5 Present Address: Department of Electrical and Computer Engineering, University of California, Santa Barbara, CA USA

## Abstract

Complex networks characterize the nature of internal/external interactions in real-world systems including social, economic, biological, ecological, and technological networks. Two issues keep as obstacles to fulfilling control of large-scale networks: structural controllability which describes the ability to guide a dynamical system from any initial state to any desired final state in finite time, with a suitable choice of inputs; and optimal control, which is a typical control approach to minimize the cost for driving the network to a predefined state with a given number of control inputs. For large complex networks without global information of network topology, both problems remain essentially open. Here we combine graph theory and control theory for tackling the two problems in one go, using only local network topology information. For the structural controllability problem, a distributed local-game matching method is proposed, where every node plays a simple Bayesian game with local information and local interactions with adjacent nodes, ensuring a suboptimal solution at a linear complexity. Starring from any structural controllability solution, a minimizing longest control path method can efficiently reach a good solution for the optimal control in large networks. Our results provide solutions for distributed complex network control and demonstrate a way to link the structural controllability and optimal control together.

## Introduction

**Figure 1:**
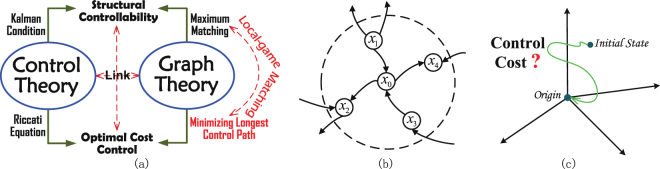
Two basic problems in controlling complex networks. (**a**) An essential issue interconnecting *graph theory* and *control theory*: how to provide a link from “structural controllability” to “optimal cost control”. (**b**) Illustration of the local topological information available to a node in a network. For a node *x*_0_ with neighbor nodes *x*_1_, *x*_2_, *x*_3_ and *x*_4_, it is assumed that the node *x*_0_ can only observe the numbers of incoming and outgoing links connected to each of the nodes *x*_1_, *x*_2_, *x*_3_ and *x*_4_. (**c**) Optimal cost control aims to determine a solution of driving a system state to any predefined state with minimum cost.

Over the past decade the complex natural and technological systems that permeate many aspects of everyday life—including human brain intelligence, medical science, social science, biology, and economics—have been widely studied^1–3^. Many of these complex systems can be modeled as static or dynamic networks, which stimulates the emergence and booming developments of research on complex networks. There are two fundamental issues associated with the control of complex networks, with different focuses on figuring out (i) *whether* the networks are controllable; and (ii) *how* to control them with least cost when they are controllable, respectively. The first issue is typically investigated by studying the *structural controllability* problem, which describes the ability to guide a dynamical system from any initial state to any desired final state in finite time. The second issue is known as the *optimal cost control* problem, with the main objective of minimizing the cost for driving the network to a predefined state with a given number of control inputs. Figure 1 illustrates the structural controllability problem and the optimal cost control problem. Note that for large complex networks without global information of network topology, both problems remain essentially open. In this work, we shall combine graph theory and control theory for tackling the two problems in one go, using only local network topology information.

Researchers are using a multidisciplinary approach to study the structural controllability of complex networks, focusing on linear time invariant (LTI)^4^ systems $\dot{{\bf{x}}}(t)=A{\bf{x}}(t)+B{\bf{u}}(t)$, where **x**(*t*) = [*x*_1_(*t*), …, *x*_N_(*t*)]^*T*^ is the state vector of N nodes at time t with an initial state **x**(0), **u**(*t*) = [*u*_1_(*t*), …, *u*_*M*_(*t*)]^T^ is the time-dependent external control input vector, and *M* (*M* ≤ *N*) is the number of inputs in which the same input u_i_(t) can drive multiple nodes. The matrix *A* = [*a*_*ij*_]_*N*×*N*_ is the weighted adjacency matrix of the network, i.e., *a*_*ij*_ ≠ 0 if there is a link connecting node *i* to node *j* and *a*_*ij*_ = 0 otherwise, and *B* = [*b*_*im*_]_*N*×*M*_ is the input matrix where *b*_*im*_ is nonzero when controller *m* is connected to node *i* and zero otherwise. The nodes have different physical meanings in different scenarios. In the Traveling Salesman Problem (TSP) it is a city or location, in a social network it is a person or group, and in an organism it could be an interacting protein. Even when networks have similar properties, a node can have a variety of interpretations in different applications. For example, in a recent work studying the structural controllability of brain networks^5^, researchers show that the neural activity process can be approximated using linearized generalizations of nonlinear models of cortical circuit activities. In their proposed LTI system, *a*_*ij*_ is the number of streamlines connecting brain region *i* to region *j*. Using an intricately detailed model of a very small region reveals whether *a*_*ij*_ describes a connection between neuron *i* and neuron *j*. Note that in this report, “controllability” always refers to “structural controllability”. Hence hereafter we shall use these two terms interchangeably for convenience of discussion.

Though there are recent literatures studying on nonlinear dynamics of complex networks^6,7^, this paper follows the mainstream work focusing on LTI systems, mainly for two reasons: (i) a lot of real-world systems can be approximated by LTI systems; the optimal control of LTI dynamics on complex networks thus forms a basis for the control and optimal control of complex systems; (ii) even for LTI dynamics on complex networks, no existing literature has considered their control and optimal cost control with only local topology information. We focus on the fundamental issues of control and optimal control of complex systems, for the first time to the best of our knowledge, demonstrating a way to link “structural controllability” and “optimal cost control” of LTI systems together. The results shall find wide applications and great potentials for further extensions in control and optimal control of complex systems. Meanwhile, there would be a long way to go in our future research to develop general methodology for control of nonlinear dynamics on complex networks.

Our work is mainly composed of two parts. In the first part, we study on the controllability problem. We show that with properly designed local operations strictly based on only local network topology information, a controllability solution can be found which is nearly as good as the optimal solution calculated using global network topology information. In the second part, we firstly propose a relatively sophisticated optimal cost control algorithm which works effectively for small or medium-sized networks. Such an algorithm has its applications in those complex systems that are not so big, and provides a benchmark for heuristic algorithm design as well. Then we propose a simple algorithm that works efficiently for large and extra-large networks. To the best of our knowledge, this is the first time that an efficient algorithm is proposed for the optimal control of large-scale complex networks. Some brief discussions on each of the three proposed algorithms are presented below, while further technical details and mathematical work can be found in the Supplementary Information (hereafter termed as SI).

Maximum matching (MM) is a concept in graph theory that has been used to address the structural controllability problem, a classic concept in control theory^8–10^. Generally speaking, MM is to find the largest set of the edges that do not share start or end nodes. A node is said to be matched if a link in the maximum matching points at it; otherwise it is unmatched. By assuming that the topological information of a network is fully known and by employing MM, the matched and unmatched nodes and edges form elementary stems and elementary circles^8^. Here an elementary stem is a directed network component consisting of N nodes 1, ...., n connected by a sequence of n − 1 directed edges {1 → 2, 2 → 3, …, n − 1 → n}, and an elementary stem becomes an elementary circle when an additional edge n → 1 is added. Note that only the starting node of an elementary stem is an unmatched node. The network controllability can be achieved when unmatched nodes are the driver nodes and each of which is connected to an independent external input. A driver node can control one of its immediate neighbors, and the propagation of control influence is through the stem. Thus all nodes on the stem can be fully controlled. On the other hand, all the matched nodes in the elementary circles do not need to be connected to extra external inputs. These nodes can be fully controlled by connecting one of the nodes in the circle to an existing external input^8^. This approach indicates which node sets are connected to a minimum number of external inputs, and subsequently reveals the input matrix *B*. Existing schemes have focused on these problems^11–13^, and they have wide significance in many real-world network applications^11,14^.

We propose a *local-game matching* (LM) algorithm to explore the structural controllability of large scale real-world networks when the global topological information of matrix *A* is absent and only local topological information is available [Fig. 1(b)]. The main idea is to form up elementary stems and elementary circles based on matching requests between adjacent nodes, using only local network topology information. We show that LM is equivalent to a static game with incomplete information as in the static Bayesian game theory^15^, a configuration common in economic or social networks, and the LM algorithm achieves a Nash equilibrium in the game (Theorems 3–4 in SI). We show that LM consistently approximates the global optimal solution found using MM, with a complexity linear in time *O*(*N*) (SI, Theorem 5). Its satisfactory performance is demonstrated in various synthetic and real-world networks (SI, Section 2.4).

**Figure 2:**
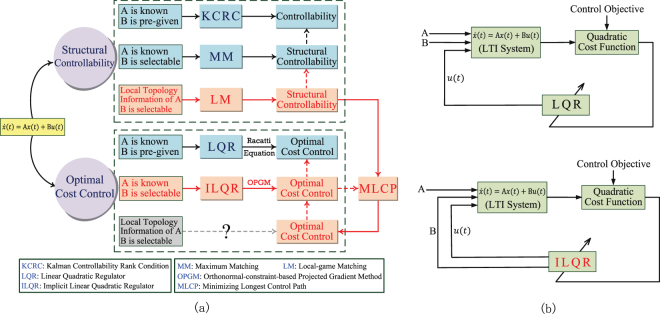
The contribution of this work in demonstrating a “link” (red solid arrows) from “structural controllability” to “optimal cost control”. In (**a**), the question mark means that currently there is no existing work considering such a problem. Our work is based on two facts: (i) To ensure “structural controllability”, we propose LM which is proven to steadily approximate the global optimal solution found by MM with linear time complexity (SI, Section 2.2); (ii) To achieve “optimal cost control”, we introduce ILQR in (**b**) to design an optimal controller for uncertain LTI systems when the input matrix *B* becomes selectable, by employing “OPGM” (SI, Section 3.2). We uncover that nodes which should be connected to external inputs tend to divide elementary topologies (stem, circle and dilation) averagely for achieving a lower cost since the control cost is mainly dependent on the length of the longest control path, which inspires (red dashed arrows) the design of the MCLP algorithm without using global topology. By combining LM and MLCP together (red solid arrows), we are able to obtain an optimal control of large scale complex networks by only using local topological information.

For the optimal control problem, we propose an orthonormal-constraint-based projected gradient method (OPGM) (SI, Section 3.2) and an implicit linear quadratic regulator (ILQR) to design an optimal controller for linear systems when the input matrix *B* is a matrix variable to be determined [Fig. 2(b)]. We find that, in the solutions, nodes connected to external inputs tend to divide the network into control paths of the same length because the control cost is strongly dependent on the length of the longest path. This finding inspires us to construct a fast and efficient *minimizing longest control path* (MLCP) algorithm without using global topology information.

As later we would see, MLCP algorithm can efficiently work out a good solution for the optimal control problem based on the results of the LM algorithm for the structural controllability. Combining LM and MLCP thus demonstrates a link (red solid line) between “structural controllability” and “optimal cost control” [Fig. 1(a)]. This allows us to control large-scale complex networks using only local topological information [Fig. 2(a)].

## Minimizing the number of driver nodes through local-game matching

Prior research has focused on structural controllability, and has not addressed method efficiency. For example, when using MM the value of N_D_ is exact but it is expensive to calculate in large networks with complexity $O(\sqrt{N}L)$. It is also extremely difficult if not impossible to apply to large real-world complex networks where global network topological information is seldom available. Even when this information is available, it is generally very difficult to control all the nodes by simply implementing MM^16^, as the communications between the central controller and so many nodes can be prohibitively expensive.

We address this issue by proposing an iterative local-game matching (LM) method. Figure 1(b) shows how we assume that each node requests only its local topology information, i.e., the input-output degrees of its immediate neighbors. We also assume that each node can initiate an action without global coordination. In a directed network, when there is a directional link from node *x*_*i*_ to node *x*_*j*_, we designate *x*_*i*_ the “parent” and *x*_*j*_ the “child.” In implementing LM, *x*_*i*_ → *x*_*j*_ → *x*_*k*_ is a matching sequence with two parent-child matches, one between nodes *x*_*i*_ and *x*_*j*_ and the other between nodes *x*_*j*_ and *x*_*k*_. When a sequence of parent-child matches forms a path, we designate it a *directed control path* (DCP) when it begins at an inaccessible node, and a *circled control path* (CCP) when it is configured end-to-end. Thus DCP and CCP correspond to elementary stems and circles in the maximum matching. To guarantee network controllability, the inaccessible nodes are connected to external inputs. To avoid confusion, all inaccessible nodes found using MM and LM are called *driver nodes*, and their numbers are denoted as *N*_*D*_ and ${N}_{D}^{LM}$, respectively. By directly controlling these driver nodes we can steer all the nodes along the control paths. We determine the minimum driver node set in a network by locating the parent-child matches for all the nodes that form directed control paths (DCPs) and circled control paths (CCPs) and minimizing the number of DCPs.

In the LM method, using local information each node requests one neighbor to become its parent and another to become its child. When there is a *match of requests* (e.g., when node *x*_*i*_ requests node *x*_*j*_ to become its parent node and node *x*_*j*_ requests node *x*_*i*_ to become its child node), a parent-child match is achieved and is fixed. The parent node then removes all of its other outgoing links, and in the iterations that follow no other node can send it a parent request. At the same time the child node removes all of its other incoming links, and in the iterations that follow no other node can send it a child request. Note that a node may send a child or parent request to itself when it has a self-loop connection. The iterative request-matching operations continue until no more child or parent requests can be sent. After implementing LM, those sequences of parent-child matches not forming a closed loop form a DCP that begins at an inaccessible node without a matched parent and ends at a node without a matched child. A closed loop (a “circle”) of parent-child matches becomes a CCP. A DCP requires an independent outside controller, but a CCP does not and can be controlled by connecting a node on the circle to any existing external control input of a directed control path. Thus the number of the independent external control inputs equals the number of DCPs found using LM.

**Figure 3:**
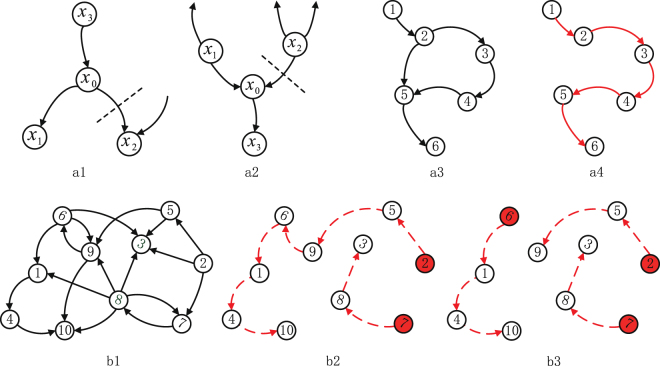
Controlling networks with local topological information by LM and MLCP. (**a**) Illustrations of the LM algorithm. In Child locating (**a1**), node *x*_0_ has two child nodes *x*_1_ and *x*_2_. As node *x*_1_ has only one parent node, while node *x*_2_ has two parent nodes, node *x*_0_ sends a child request to *x*_1_. In Parent locating (**a2**), Node *x*_0_ has two parent nodes *x*_1_ and *x*_2_. As node *x*_1_ has two child nodes while node *x*_2_ has three child nodes, node *x*_0_ sends a parent request to *x*_1_. And a simple example of LM is shown in (**a3**), where node 2 and node 3 receive parent request and child request from each other. These two nodes match with each other. Thus, LM gives the same result as MM in this example. (**b**) Controlling a network with LM and MLCP. Firstly, obtain the control paths (DCPs and CCPs) by LM. To control the network in (**b1**), node 2 and node 7 (red color in (**b2**)) form up the driver node set which should be connected to the external control inputs. Note that the driver node set for a network may not be unique. If we add one more external input to the network, as seen in (**b3**), the new input node will be added on *node 6* by applying *minimizing longest control path* (MLCP). More detailed examples can be seen in SI, Section 4.

When a node is seeking a match, we define the current number of its *unmatched* child (parent) nodes, i.e., the nodes that have not yet achieved a match with a parent (child), as its u-output (u-input) degree. To increase the probability that a match of requests will take place, we have each node send a child (parent) request to the unmatched neighbor child (parent) node with the lowest u-input (u-output) degree. Figure 3(a1) and (a2) show a simple example. Because we assume that nodes with lower u-input (u-output) degrees will on average receive fewer child (parent) requests, we expect that this technique will increase the probability of achieving a match and thus lower the probability that a node will become a driver node with no match. A simple example in Figure 3(a3 and a4 shows that by using this simple strategy the LM gives the same result as the MM.

When there is a tie, i.e., when a node has multiple unmatched child (parent) nodes with the same minimum u-input (u-output) degree, the node can either do nothing with a waiting probability ${\omega }$, or it can break the tie randomly at this iteration step. Our experiments show that introducing waiting probability ${\omega }$ into the LM method improves its performance in certain cases (SI, Section 2.6). A detailed description of the LM algorithm (the codes are available at https://github.com/PinkTwoP/Local-Game-Matching) and a few examples showing step-by-step execution of it can be found in Section 2.1 of SI.

In LM, each node tends to maximize its own chance to be matched, collecting and using only local topological information to quickly accomplish matches as far as such is possible, thus allowing LM to be used in large-scale complex networks. It is shown that the LM algorithm is equivalent to the static Bayesian game with incomplete information (SI, Theorem 4). Because this configuration is common in real-world complex economic and social networks, LM helps us understand them.

**Figure 4:**
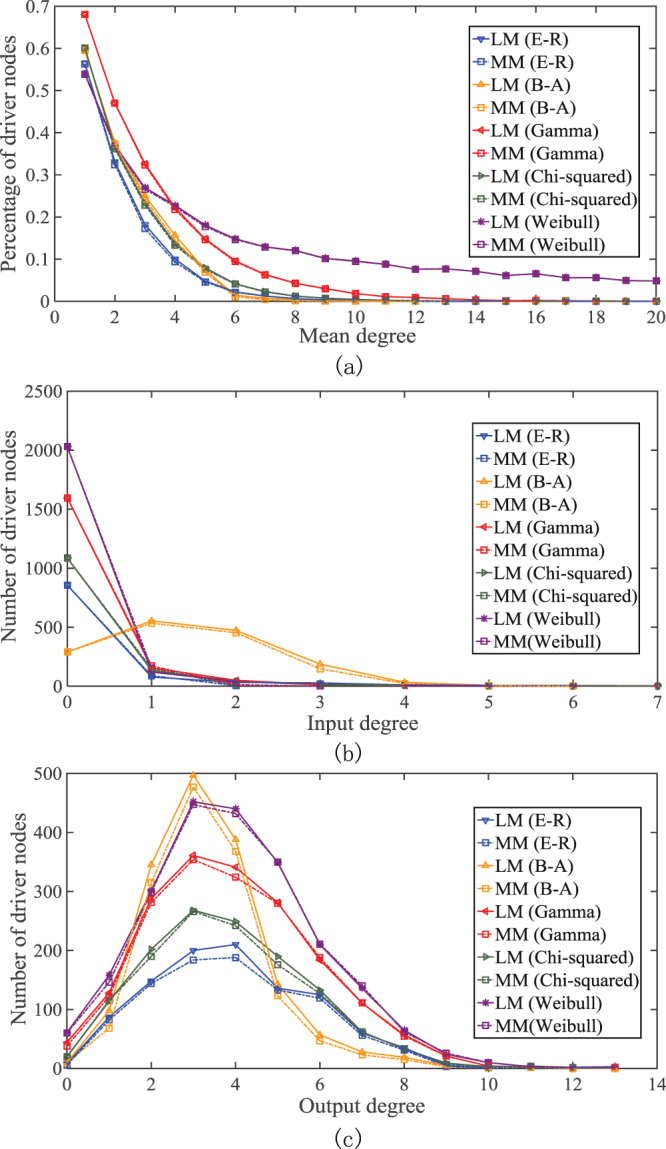
LM and MM in synthetic networks: (**a**) Number of driver nodes with respect to the mean degree μ for ER, B-A and other networks for LM and MM (N = 10000). (**b**) Input nodal degree distributions of the driver nodes found by LM and MM, respectively (*N* = 10000 and *μ* = 6). (**c**) Output degree nodal degree distributions of the driver nodes found by LM and MM, respectively.

We test the LM method on synthetic and real-life networks. The synthetic networks include the ER model^17^, the BA network^18,19^, and networks generated using Chi-squared, Weibull and Gamma distributions, respectively. Topology information about all the real-life networks we have tested is available from open sources (see the reference citations in Table S3). Figure 4(a) shows the percentage of driver nodes identified by the LM and MM methods in the synthetic networks with different average nodal degrees. The results for real-life networks are summarized in Table S3 of SI. It is observed that the number of driver nodes identified by the LM method are consistently to be close to or equal to the optimal solutions identified by the MM method in both synthetic and real-life networks.

Although the numbers of driving nodes found by MM and LM in different networks are about the same, an immediate question that arises is whether the driver nodes identified by the two different methods have similar statistical properties. Figure 4(a–c) show that the two methods produce approximately the same number of driver nodes and that they also identify the nodes with approximately the same input and output degree distributions. In addition, it is easy to see that the driver nodes generally avoid the hubs, which is consistent with the results in^8^. Thus, we conclude that the two methods either find approximately the same set of nodes, or find two sets of nodes with approximately the same statistical properties. The sub-optimality of the LM method in these synthetic networks is verified.

The SI further supplies formal proofs suggesting that (i) network’s structural controllability is guaranteed by LM (SI, Theorem 1); (ii) LM minimizes the probability that augmented paths will be formed based on local topological information and thus reduces the number of required external control inputs (SI, Theorems 2–3); and (iii) the Nash equilibrium of the Bayesian game can be achieved by LM (SI, Theorem 4). This theoretically explains why the solution of the LM method approximates the global optimal solution of the MM method in a number of synthetic and real-world networks (SI, Tables S2 and S3). We also prove that the time complexity of the LM algorithm is linear *O*(*N*) (SI, Theorem 5), which is much lower than that of MM *O*($\sqrt{N}L$)^20,21^ and comparable to the state-of-the-art approximation algorithms in graph theory^22,23^. The difference between the LM and prior algorithms is that it uses much less local topological information when approximating maximum matching (SI, Section 1.3).

## Minimization of the cost control

### Implicit linear quadratic regulator (ILQR)

Although the controllability of complex networks is an important concern, minimizing the cost of control is even more important. Simply knowing the number of driver nodes does not tell us how to design an optimal controller for a given particular control objective. Figure 1(a) shows how control theory and graph theory can be used to determine the optimal cost control, a critical problem in complex network control. The objective is to find an optimal or suboptimal input matrix B^*^ with a fixed dimension *M* without access to global topological information about adjacency matrix *A*. Although there have been some recent studies on the relationship between network controllability and control cost^24–26^, this is an issue that traditional control theory has not considered.

Traditional control theory allows us to design an input signal **u**(*t*) with the lowest cost when the systems are LTI and the cost function is quadratic. If both the topological connection matrix *A* and input matrix *B* are known^27^, this linear quadratic (LQ) problem provides a solution given by a linear-quadratic regulator (LQR) that involves solving a set of complicated Riccati differential equations^28,29^. However, LQR cannot be used in large-scale real-world networks because (i) solving a high-dimension Riccati differential equation is difficult and time consuming; and (ii) the Riccati equation requires global information of network topology *A* and input matrix *B*, which is seldom available for large-scale real-world networks. To address this issue we use a constrained optimization model for the optimal cost control problem in which the controller determines the *B* variable. Once the input matrix *B* is obtained, the optimal controller can be constructed. This optimal controller is called an implicit linear quadratic regulator (ILQR) because it is implicitly dependent on *B*. Figure 2(b) shows how ILQR differs from LQR. The only decision variable to be determined in LQR is **u**(*t*). The value of the input signal at each operating time **u**(*t*) and the nodes to which the inputs are connected and the connection weights *B* are both decision variables to be determined using ILQR.

We formulate ILQR as a matrix optimization problem under an orthonormal boundary condition, where the objective is to drive the states from any initial state **x**_0_ = **x**(0) = [*x*_1_(0), ...., *x*_*N*_(0)]^*T*^ ∈ R^*N*×1^ to converge to the origin during the time interval [0, t_*f*_] using a minimum cost defined by ${\mathbb{E}}\{{\int }_{0}^{{t}_{f}}{{\bf{u}}}^{T}(t){\bf{u}}(t)dt\}$^30^.

When **u**(*t*) is given by ${\bf{u}}(t)=-{B}^{T}{e}^{{A}^{T}({t}_{f}-t)}{W}_{B}^{-1}{e}^{A{t}_{f}}{{\bf{x}}}_{0}$^31,32^ the system state is driven to the origin. As when the input matrix B is selectable, both **x**(*t*) and **u**(*t*) become functions of *B*, which are denoted as **x**(*t*) = **x**(*t*, *B*) and **u**(*t*) = **u**(*t*, *B*), respectively. We thus present a constrained non-convex matrix optimization problem with the input matrix *B* ∈ R^N × M^ as its variable,

1 $$\begin{array}{lll} &  & {{\rm{\min }}}_{B}\,\,{\mathbb{E}}(B)={\mathbb{E}}[{\int }_{0}^{{t}_{f}}{\Vert {\bf{u}}(t,B)\Vert }^{2}dt]=tr({[{\int }_{0}^{{t}_{f}}{e}^{At}B{B}^{T}{e}^{{A}^{T}t}dt]}^{-1}{e}^{A{t}_{f}}{\mathbb{E}}({{\bf{x}}}_{0}{{\bf{x}}}_{0}^{T}){e}^{{A}^{T}{t}_{f}})\\  & s\mathrm{.}t\mathrm{.} & (A,B)\,is\,controllable,\,\,\,{B}^{T}B={I}_{M}\\  &  & \dot{{\bf{x}}}(t)=A{\bf{x}}(t)+B{\bf{u}}(t),\,\,\,{\bf{x}}\mathrm{(0)}={{\bf{x}}}_{0},\,{\bf{x}}({t}_{f})={\bf{0}}\end{array}$$

where ${\bf{x}}(t)={[{x}_{1}^{T}(t),\mathrm{...,}{x}_{M}^{T}(t)]}^{T}$ and ${\bf{u}}(t)={[{u}_{1}^{T}(t),\mathrm{...,}{u}_{M}^{T}(t)]}^{T}$ with M being the number of control inputs, ${\mathbb{E}}[B]$ is the expectation of the control cost of driving the system from an arbitrary initial state to the origin (**x**_*f*_ = **x**(t_*f*_) = **0**) during the time interval [0, t_*f*_]. Here ${\mathbb{E}}[\cdot ]$ is the argument over all realizations of the random initial state, *tr*(.) is a matrix trace function, and *I*_*M*_ is an identity matrix with a dimension *M*. Note that a necessary condition for (*A*, *B*) to be controllable is that *M* ≥ *N*_*D*_. In fact, the constraint on the controllability of (*A*, *B*) implies that the Gramme matrix ${W}_{B}=[{\int }_{0}^{{t}_{f}}{e}^{At}B{B}^{T}{e}^{{A}^{T}t}dt]$ is invertible, and *B*^*T*^*B* = *I*_*M*_ refers to the orthonormal boundary condition under which all columns of *B* are orthogonal to each other. The derivation of the model and discussion on the orthonormal constraint are presented in Section 3 of SI. By assuming that each element of the initial state **x**_0_ is an identical independently distributed (i.i.d) variable with zero mean and variance 1, we have ${\mathbb{E}}[{{\bf{x}}}_{0}{{\bf{x}}}_{0}^{T}]={I}_{N}$ in Equation (1).

Because the above nonlinear constrained optimization problem has complicated matrices as its variables, it is difficult to obtain a solution. The challenge is to obtain the gradient of the cost function, which involves a series of nonlinear matrices-by-matrices derivatives that are not widely considered. We address this problem by proposing an iterative algorithm, the orthonormal-constraint-based projected gradient method (OPGM), on Stiefel manifolds for designing ILQR (SI, Section 3)

2 $$\begin{array}{ccc}{\hat{B}}_{k+1} & = & {B}_{k}-\eta \cdot ({I}_{N}-{B}_{k}{B}_{k}^{T})\cdot \nabla {\mathbb{E}}({B}_{k})\\ {B}_{k+1} & = & \sqrt{\frac{tr({\hat{B}}_{k+1}^{T}{\hat{B}}_{k+1})}{tr({\hat{B}}_{k+1}^{T}{\hat{B}}_{k+1}{\hat{B}}_{k+1}^{T}{\hat{B}}_{k+1})}}\cdot {\hat{B}}_{k+1},\end{array}$$

where η is the learning step, $\nabla {\mathbb{E}}({B}_{k})$ is the gradient $\nabla {\mathbb{E}}(B)$ at *B* = *B*_*k*_, and we have

3 $$\nabla {\mathbb{E}}(B)=-\frac{\partial {\mathbb{E}}(B)}{\partial B}=-{\int }_{0}^{{t}_{f}}2{e}^{{A}^{T}t}{W}_{B}^{-T}{e}^{A{t}_{f}}{\mathbb{E}}({{\bf{x}}}_{0}{{\bf{x}}}_{0}^{T}){e}^{{A}^{T}{t}_{f}}{W}_{B}^{-T}{e}^{At}dt\cdot B$$

Therefore, throughout the process in ILQR, both u(*t*) and *B* are decision variables to be determined, where u(*t*) specifies the values of controller inputs at each operating time and B determines the nodes to which the controller inputs are connected and the weights of the connections. We prove that the iteration is convergent (SI, Theorem 7), with ${\mathbb{E}}({B}_{k})$ converging to ${\mathbb{E}}({B}^{\ast })$ where B^*^ is an orthonormal matrix, i.e., B^*T^B^*^ = *I*_*M*_ if *η* is sufficiently small.

**Figure 5:**
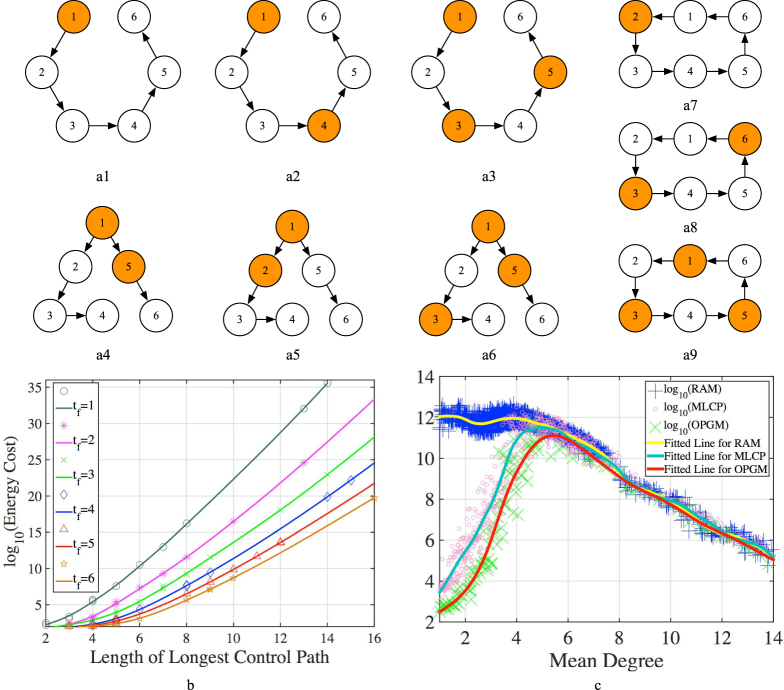
Interesting observations of ILQR. (**a**) Illustration that control nodes tend to divide elementary topologies averagely for consuming less energy cost as the cost is mainly dependent on the length of the longest control path. The located control nodes are marked in an elementary stem and circle in (**a1**–**a3**) and (**a7**–**a9**) (*M* = 1, 2, 3) and in an elementary dilation with (**a4**–**a6**) (*M* = 2, 3). When *M* = 2, the control node set converges to either {*node* 1, 5} in case a4 or {*node* 1, 2} in case a5 with different probabilities, around 34.19% and 65.81% respectively in 10000 rounds of experiments. When *M* = 3, the control node set approaches {*node* 1, 4, 5} and {*node* 1, 3, 5} with percentages 24.64% and 75.36%, respectively, in 10000 rounds of experiments. (**b**) Illustration that the control cost is proportional to the longest control path. Here *M* external control inputs are randomly allocated on a 100-node elementary circle, and the control cost vs the longest control path that is just the maximum values of all the number of edges between any two adjacent control nodes, are recorded. The experiments are simulated on Matlab with higher precision (130 significant digits) by using the Advanpix multi-precision computing toolbox. (**c**) Illustration that MLCP performs significantly better than *Random Allocation Method* (RAM), in low-degree networks while they become almost indistinguishable as the mean degree (mean in-/out-degree) of the networks becomes dense. The experiment is done on an ER network by adding edges randomly and persistently (SI, Section 4.4), with *M* being given by $M={N}_{D}^{LM}+{m}_{0}$ (*m*_0_ = 100). The mean degree increases as more edges are continuously added, and the three fitting curves plotted for RAM, OPGM and MLCP respectively coincide with each other when mean degree is around 6.

Our objective is to control the system at the lowest cost using the lowest possible number of independent control inputs determined without knowing the global topology of the network, i.e., to find the optimal input matrix B^*^ when global topological information about both *A* and *B* is unavailable. Our immediate task is to locate the *control nodes*, i.e., the nodes directly connected to external inputs for minimizing control cost. Math work for developing the relatively complicated OPGM is presented in detail in Section 3.2 of SI. Figure 5(a,1–a9) shows that by employing OPGM in three elementary topologies, control nodes divide the three topologies averagely for a lower energy cost, and the control energy is strongly dependent on the length of the longest control path [see Fig. 5(b)]. This finding enables us to design a minimizing longest control path (MLCP) method, an efficient scheme for controlling large-scale complex networks when there are sufficient control nodes to avoid the numerical controllability transition area^33^.

### Minimizing the Longest Control Path (MLCP)

Finding the minimum number of driver nodes using maximum matching N_D_ is insufficient for controlling real-world complex networks. When we attempt to gain control by imposing input signals on the minimum set of driver nodes indicated by structural controllability theory^25^, we may not be able to reach the target state because too much energy is required. This is known as the *numerical controllability transition* where large networks often cannot be controlled by a small number of drivers^33^ even when existing controllability criteria are satisfied unambiguously. The reason is that a small number of driver nodes is barely enough to ensure controllability due to the fact that the *controllability Gramian* may be ill-conditioned. Thus we need to set the number of control nodes connected to external control inputs to be sufficiently large. Basically the numerical success rate increases abruptly from zero to approximately one as the number of control inputs is increased^33^.

After implementing LM, a number of DCPs and CCPs form in the network. Based on the fact that control nodes tend to divide elementary topologies on an average, we design the minimizing longest control path (MLCP) algorithm, an efficient scheme for controlling large-scale complex networks for *M* control inputs such that $M={N}_{D}^{LM}+{m}_{0}$, where ${N}_{D}^{LM}$ is approximately the same as *N*_*D*_ and *m*_0_ ≥ 0 is large enough for the number of controllers to go beyond the *numerical controllability transition area*.

The main idea of MLCP is to make each DCP to be of nearly the same length as much as possible with minimum changes to the results got by LM. We assume that we have ${N}_{D}^{LM}$ DCPs and *L*_*i*_ is the length of path *i* for $1\le i\le {N}_{D}^{LM}$. We add *m*_0_ additional control inputs to these paths to minimize the longest path length of the newly formed paths. If *n*_*i*_ is the number of additional control inputs added on the path *L*_*i*_ subject to ${\sum }_{i=1}^{{N}_{D}^{LM}}{n}_{i}={m}_{0}$, then MLCP is formulated as a min-max optimization problem,

4 $$\begin{array}{ll}{\rm{\min }} & {\rm{\max }}\,\,\,\{\frac{{L}_{i}}{1+{n}_{i}}\}\\ s\mathrm{.}t\mathrm{.} & \sum _{i=1}^{{N}_{D}^{LM}}{n}_{i}={m}_{0}\\  & 0\le {n}_{i}\le {m}_{0}\end{array}$$

where $\{\frac{{L}_{i}}{1+{n}_{i}}\}$ is the sequence of $\{\frac{{L}_{i}}{1+{n}_{i}}\}$ for all *i*. Thus the longest control path is ${\rm{m}}{\rm{a}}{\rm{x}}{\ulcorner}\{\frac{{L}_{i}}{1+{n}_{i}}\}\urcorner$ where ${\ulcorner}\cdot {\urcorner}$ is a ceiling function. Figure 3(b) shows an example. After applying LM the lengths of two directed control paths are *L*_1_ = 7 and *L*_2_ = 3, respectively. Then the longest control path length is *max*{7, 3} = 7. Figure 3(b2 and b3 shows that when *m*_0_ = 1 the new control input is added to *L*_1_ by MLCP, giving *n*_1_ = 1, *n*_2_ = 0, and *n*_1_ + *n*_2_ = 1. Thus the longest control path length of the newly formed paths is ${\rm{\max }}\{{\ulcorner}\frac{7}{1+1}{\urcorner},3\}=4$

When both DCPs and CCPs exist after applying LM, and CCP does not require an additional external control input, we assign each CCP to a particular DCP and have a new sequence of *L*_*i*_ for $1\le i\le {N}_{D}^{LM}$. Thus assigning *m*_0_ additional inputs can be done by MLCP. A more detailed illustration is given in SI, Section 4.

MLCP is applied to synthetic networks including ER networks^17^, BA networks^18,19^, and a number of real-world networks, and comparisons to OPGM and a random connection method between controllers and network nodes are drawn. Note that in order to generate this random connection method that ensures network controllability we first apply the MM method to find one set of driver nodes, which can be any one among the multiple maximum matching solutions for the network. Using the simple *random allocation method* (RAM) we then randomly select *M* − *N*_D_ additional network nodes to construct the control node set to be connected to external inputs. Figure 5(c) presents simulation results on synthetic networks, while extensive results on real-world networks are summarized in Tables S5 and S6 in SI. We conclude that MLCP performs comparably to OPGM although network nodes are restricted to binary connections to external inputs (this shows the validity of MLCP), and it performs better than RAM. As an increasing number edges are added both MLCP and RAM are only slightly inferior to OPGM, and they become nearly indistinguishable as the network density increases. This is because when adding edges onto a low-degree network, the required number of driver nodes gradually reduces and the average/maximum length of control paths becomes longer, which causes a higher control cost. However, with the network becoming further denser, the number of paths from an arbitrary node *x*_*i*_ to another arbitrary node *x*_*j*_ increases, implying many possibilities and opportunities for *x*_*i*_ to affect *x*_*j*_. This makes the required driver nodes reduce insignificantly but the average/maximum length of control paths become shorter, which drastically decreases the control cost. Thus the performance of MLCP finally converges to that of RAM. This is a significant finding for complex network control. In large-scale dense networks we can simply randomly select the control nodes to obtain an optimal cost control, but for lower degree networks MLCP is the best choice.

## Discussions and Conclusion

We begin by proposing local-game matching (LM) to ensure the structural controllability of complex networks when we have incomplete information about the network topology, and we test the performance using real-world networks with millions of nodes. We then design a suboptimal controller, the “implicit linear quadratic regulator” (ILQR) for LTI systems with incomplete information about the input matrix. It is found that the control cost can be significantly reduced if we minimize the longest control path length. This conclusion is consistent with the findings in^30,34^. Thus, by combining the LM and MLCP, we are able to demonstrate a “link” from “structural controllability” to “optimal cost control” in complex networks without using the global topology. We can apply MLCP to select control nodes in networks of relative lower degrees, while in dense networks the random selection of control nodes is effective. As commonly most real-world networks can be modeled using LTI systems with various assigned physical meanings of nodes and edges, we believe the methodology we propose here can be applied to many real-world networks studied in the human brain, medical science, social science, biology, and economics. Furthermore, many physical constraints may exist in real-life systems, affecting the selection of matrix *B*. While some studies have been carried out to handle such constraints^35^ and MLCP may be viewed as to a certain extent helping fulfill the simple control of large-scale complex systems, extensive further studies are in demand to handle various constraints in real-life applications.

In this work, we uncover that the local network topology information of directed networks provides sufficient rich information not only for modelling real world, but also for brain inspired computing. It is well known that the human brain can be described as an extremely large-scale complex networks having 10^11^ nodes (neurons) and 10^15^ edges (synapses), with scale-free organization. And many studies indicate that the brain neural networks are organized by dense local clustering to efficiently support the distributed multi-modal information processing, including vision, hearing, olfaction, etc. These characteristics make it possible for human brain works efficiently as parallel-distributed processing computers with various circuits that process local information parallelly and distributedly. We envision that our breakthrough on the control and optimal control of complex network with limited topological information would exploit advances in future brain-inspired computing theory aiming for deliver cost-effective information processing.

## Electronic supplementary material

Supplementary Information
