# Author Correction: Enabling Controlling Complex Networks with Local Topological Information

**DOI:** 10.1038/s41598-018-25617-z

**Published:** 2018-05-22

**Authors:** Guoqi Li, Lei Deng, Gaoxi Xiao, Pei Tang, Changyun Wen, Wuhua Hu, Jing Pei, Luping Shi, H. Eugene Stanley

**Affiliations:** 1 Center for Brain Inspired Computing Research, Department of Precision Instrument, Tsinghua University, Beijing, P. R. China; 2 School of Electrical and Electronic Engineering, Nanyang Technological University, Singapore, Singapore; 3 Center for Polymer Studies, Department of Physics, Boston University, Boston, USA; 4 Beijing Innovation Center for Future Chip, Tsinghua University, Beijing, P. R. China; 5 Present Address: Department of Electrical and Computer Engineering, University of California, Santa Barbara, CA USA

Correction to: *Scientific Reports* 10.1038/s41598-018-22655-5, published online 15 March 2018

The Acknowledgements section in this Article is incomplete.

“The work was partially supported by National Science Foundation of China (61603209), and Beijing Natural Science Foundation (4164086), and the Study of Brain-Inspired Computing System of Tsinghua University program (20151080467), and Ministry of Education, Singapore, under contracts RG28/14, MOE2014-T2-1-028 and MOE2016-T2-1-119. Part of this work is an outcome of the Future Resilient Systems project at the Singapore-ETH Centre (SEC), which is funded by the National Research Foundation of Singapore (NRF) under its Campus for Research Excellence and Technological Enterprise (CREATE) programme.”

should read:

“The work was partially supported by National Science Foundation of China (61603209, 61327902), and Beijing Natural Science Foundation (4164086), and the Study of Brain-Inspired Computing System of Tsinghua University program (20151080467), and SuZhou-Tsinghua innovation leading program 2016SZ0102, and Ministry of Education, Singapore, under contracts RG28/14, MOE2014-T2-1-028 and MOE2016-T2-1-119. Part of this work is an outcome of the Future Resilient Systems project at the Singapore-ETH Centre (SEC), which is funded by the National Research Foundation of Singapore (NRF) under its Campus for Research Excellence and Technological Enterprise (CREATE) program.”

