# Supplementary material for: Enabling Controlling Complex Networks with Local Topological Information
Source: Sci Rep. 2018 Mar 15;8:4593. doi: 10.1038/s41598-018-22655-5 (PMC5854593; doi:10.1038/s41598-018-22655-5)
Supplement: Supplementary file 1 — Supplementary Information [file 41598_2018_22655_MOESM1_ESM.pdf]

Enabling Controlling Complex Networks with Local Topological Information

Supplementary Information

Guoqi Li[1,4*†], Lei Deng[1,5*], Gaoxi Xiao[2*†], Pei Tang[1,4*], Changyun Wen[2], Wuhua Hu[2], Jing Pei[1,4], Luping Shi[1,4†], and H. Eugene Stanley[3]

[1] Center for Brain Inspired Computing Research, Department of Precision Instrument, Tsinghua University, Beijing, P. R. China.

[2] School of Electrical and Electronic Engineering, Nanyang Technological University, Singapore.

[3] Center for Polymer Studies, Department of Physics, Boston University, Boston, USA.

[4] Beijing Innovation Center for Future Chip, Tsinghua University, Beijing, P. R. China.

[5] Department of Electrical and Computer Engineering, University of California, Santa Barbara, CA, USA.

[*] The authors contribute equally to this work.

[†] liguoqi@tsinghua.edu.cn or egxxiao@ntu.edu.sg or lpshi@mail.tsinghua.edu.cn.

Contents

1 Introduction **6**

1.1 Abbreviations 6

1.2 Concept Definitions 6

1.3 Related works 7

1.4 Organization of this Supplementary Information 9

1.5 The List of Notations in this Supplementary Information 10

2 Local-game Matching (LM) **13**

2.1 Examples of LM in small networks 14

2.2 Theoretical Analysis of Local-game Matching 17

Lemma 1 17

Theorem 1 17

Theorem 2 20

Theorem 3 21

Theorem 4 25

Theorem 5 28

2.3 Definitions of the networks with other distributions 34

2.4 Performance of Local-game Matching 35

2.5 Empirical estimation of the number of iteration steps 36

2.6 Effects of the waiting probability w. 41

Theorem 6 41

3 Implicit Linear Quadratic Regulator **45**

3.1 Problem formulation of ILQR 45

3.2 Convergence Proof of OPGM 48

Lemma 2 48

Lemma 3 48

Lemma 4 49

Lemma 5 49

Lemma 6 50

Lemma 7 50

Theorem 7 51

3.3 Control node selection 56

3.4 Comparisons between PGM and OPGM 56

4 Minimizing Longest Control Path **57**

4.1 MLCP Algorithm 58

4.2 Example illustration 58

4.3 Performance of Minimizing Longest Control Path 62

4.4 Experiments and Analysis for Figure 4(c) in the Main Paper 62

Tables

Table S1 **Notations in this Supplementary Information** 10

Table S2 **The absolute errors between LM and MM in synthetic networks** 36

Table S3 **The absolute errors between LM and MM in real-life networks** 38

Table S4 **Comparisons of OPGM and PGM on an elementary dilation.** 57

Table S5 **Network Experiment Result** 61

Table S6 **Dense Network Experiment Result** 61

Figures

Figure S1 **Flowchart of implementing the LM algorithm** 14

Figure S2 **An example of the LM method in a small network** 15

Figure S3 **A node x_i matches with another node** 16

Figure S4 **Proof illustration of Theorem 1** 18

Figure S5 **An example of network after implementing the LM algorithm** 19

Figure S6 **An example of bipartite graph construction** 20

Figure S7 **Illustration of the connection between v_i^+ and v_j^-** 22

Figure S8 **Estimating the probability that an augmenting path formed** 23

Figure S9 **An example of the probable changes in the local region of v_x and v_y** . . . 29

Figure S10 **LM and MM in synthetic networks** 37

Figure S11 **Number of iterations vs. number of matched nodes** 39

Figure S12 **Number of iterations in synthetic networks at $w = 0$** 39

Figure S13 **Number of iterations in synthetic networks at $w = \frac{2}{3}$** 40

Figure S14 **LM in real-life networks: number of iterations vs. mean nodal degree** . 42

Figure S15 **LM in real-life networks: number of iterations vs. mean nodal degree** . 43

Figure S16 **Having a non-zero waiting probability w helps improve LM's performance** 44

Figure S17 **Control nodes by OPGM and PGM on an elementary dilation** 57

Figure S18 **An example of MLCP algorithm** 59

1 Introduction

1.1 Abbreviations

LM: Local-game Matching

MM: Maximum Matching

LTI: Linear Time Invariant

KCRC: Kalman Controllability Rank Condition

LQR: Linear Quadratic Regulator

ILQR: Implicit Linear Quadratic Regulator

LQG: Linear Quadratic Gaussian

PGM: Projected Gradient Method

OPGM: Orthonormal-constraint-based Projected Gradient Method

MLCP: Minimizing Longest Control Path

DCP: Directed Control Path

CCP: Circled Control Path

RAM: Random Allocation Method

1.2 Concept Definitions

Structured LTI system: An LTI system $\dot{\mathbf{x}}(t) = A\mathbf{x}(t) + B\mathbf{u}(t)$ where A and B are structured matrices, i.e., their elements are either fixed zeros or independent free parameters.

Structural Controllability: A concept introduced by Lin in 1970s [1], which refers to the ability to drive a structured LTI system to any state in its entire configuration space using only certain admissible complex system manipulations. The goal of a structural controllabiity problem is typically to allocate connections between a minimum number of external control inputs and network nodes to ensure the network controllability. A system is termed as *structurally controllable* if it is possible to fix the independent free parameters in A, B to certain nonzero values such that system (A, B) is controllable in the usual sense, i.e., the Kalman's Controllability Rank Condition (KCRC) is satisfied. Thus, a structurally controllable system is controllable for almost all values of free parameters except for those in some proper algebraic variety in the parameter space [2] .

Neighbors : A node x_j is a neighbour of x_i if there exists at least one edge (either $x_i \to x_j$ or $x_j \to x_i$) between them.

Local Topology Information: A node x_0's local information is defined as the input and output degrees of all its neighbors.

Elementary stem: A directed network component consisting of N nodes $x_1, ..., x_N$ connected by a sequence of $N-1$ directed edges $x_1 \to x_2, x_2 \to x_3, ..., x_{N-1} \to x_N$.

Elementary circle: An elementary stem becomes an elementary circle when an additional edge $x_N \to x_1$ is added.

Elementary dilation: A directed network component in which two or more elementary stems share the same starting node x_1.

For the implementation of LM, we define:

Unmatched/Matched node: A node that is inaccessible/accessible.

Directed control path: A matching sequence of parent-child nodes which starts at an unmatched node.

Circled control path: A matching sequence of parent-child nodes which forms into an elementary circle.

Driver node: An unmatched/inaccessible node. As will be discussed, a driver node set contains the minimal set of nodes connected to external inputs for ensuring structural controllability of the network.

Control node: A node that is directly connected to an external control input, either for controllability or minimum cost control purpose.

For the implementation of MLCP, two concepts need to be defined to avoid confusion.

Independent control node: A node that is connected to a newly added external control input.

Dependent control node: A node that is connected to an existing external control input.

1.3 Related works

As illustrated in Figure 2(a) in the main paper, the main contribution of this article is the establishment of a "link" (red line) from "structural controllability" to "optimal cost control" utilizing *local-game matching* (LM) algorithm and *minimizing longest control path* (MLCP) algorithm. We uncover that local network topology information is sufficiently rich for enabling efficient control of extremely large real-life networks. It is also observed that, for large scale relatively dense networks, a simple scheme of randomly selecting control nodes works efficiently as a suboptimal solution. In this contribution, "local-game matching (LM)", "implicit linear quadratic regulator (ILQR)" and "minimizing longest control path (MLCP)" are three essential components establishing the red link from "structural controllability" to "optimal cost control".

The Local-game Matching is a distributed, local-information based parallel algorithm for achieving the Maximum Matching in a directed network. In the past decades, a few distributed algorithms have been developed for tackling the maximum matching problem. The headstream can be traced to Hopcroft and Karp [3], who adopted a depth-first searching method to find a maximal set of augmenting paths, based

on which a maximum matching can be found. Thereafter, a few more distributed/parallel algorithms have been proposed by Israeli and Itai [4], Schieber and Moran [5], and Wu and Loui [6] et al., which however request certain global topology information such as the distance between every pair of nodes or the degree distribution of the network etc [6].

Recently, further improvements have been made and a few local information based maximum matching methods have been proposed. Specifically, Hoepman [7] developed a distributed $O(n)$-time algorithm to find weighted matching in general graphs; Lotker [8] proposed a more general distributed algorithm for finding the maximum matching on weighted or unweighted, static or dynamic graphs; and Mansour and Vardi [9] designed a local approximation algorithm which has a high chance to solve the maximum matching problem with a low complexity of $O(\log^4(n))$.

The most significant difference between LM and the existing local information based algorithms is that LM requests the least amount of local information. For example, in the state-of-art algorithms proposed in [8,9], the information obtained by every node is developed from its immediate neighborhood to its radius-$r(k)$ neighborhood after k steps of algorithms, where $r(k)$ is a scale function of k. The greedy algorithm proposed in [7] further allows every node to discover the topology of the whole network. The LM algorithm, on the contrary, requests only limited local information throughout the whole process, hence eliminating the need of any sophisticated multi-hop information propagations.

We now briefly review some existing results related to Implicit Linear Quadratic Regulator (ILQR). Compared with linear quadratic regulator (LQR) [10] [11], a well-known result for *optimal control*, we take one step further to consider the case where the input matrix of the LTI system is selectable. On the other hand, ILQR can be viewed as related to the *Linear Quadratic Gaussian* (LQG) problem [12,13], one of the fundamental optimal control problems. The similarity lies in that both ILQR and LQG consider the control of uncertain linear systems. In an LQG problem, the uncertainty is due to additive white Gaussian noises [14], resulting in incomplete state information. In ILQR, on the other hand, uncertainty arises since we do not know which nodes are connected to output controllers, thus having incomplete input matrix information as well as incomplete state information. It should also be pointed out that the optimization problem of ILQR formulated in this article is non-convex, which requests careful heuristic algorithm design.

There are some other related works, including those on "pinning controllability" [15], "network synchronizability" [16] [17], and consensus or agreement in multi-agent systems [18], etc. Such studies have their main focuses on whether the system can exhibit specific spatio-temporal symmetries (for instance, whether all network nodes can follow a common time-varying trajectory or converge to a common value or achieve a common goal) and the robustness of such symmetries. For example, there are algorithms

for selecting "leader agents" in stochastically forced consensus networks to minimize the mean-square deviation from the consensus. The problem we address in this paper is fundamentally different from those in existing works. We study on whether the system can fully explore its state space for system control and the cost needed when applicable.

In conclusion, the fundamental difference between existing works and our study is that we exploit the networks' local topology information to achieve the optimal control objective. Essential attributes of our proposed algorithms allow the proposed methods to be truly distributed and local-information based, and hence suitable for optimal control of large scale complex networks.

1.4 Organization of this Supplementary Information

This Supplementary Information is organized as follows. From Section 2 to Section 4, the Supplementary materials for Local-game Matching (LM), Implicit Linear Quadratic Regulator (ILQR) and Minimizing the longest Control Path (MLCP) methods are presented, respectively. Specifically,

- In Section 2, we first present a formal description of the Local-game Matching (LM) algorithm (Section 2.1), followed by two examples of LM in small networks (Section 2.2). In Section 2.3, it is proven that LM is equivalent to a static game with incomplete information, named as static Bayesian game in game theory, and that LM achieves Nash equalibrium of the game. Also, LM steadily approximates the global optimal solution found by MM, with a linear time complexity $O(N)$. Definitions of the networks with a few different nodal-degree distributions are presented in Section 2.4 and performance of LM in various synthetic and real-life networks is demonstrated in Section 2.5. Finally, experimental results on the number of iterations needed and the effects of waiting probability w are illustrated and discussed in Sections 2.6 and 2.7, respectively.

- In Section 3, the formulation of the optimization problem of ILQR is discussed in Section 3.1. The convergence of "Orthonormal-constraint-based Projected Gradient Method" (OPGM) on Stiefel manifolds is proven in Section 3.2, followed by some discussions on the control node selection in Section 3.3. Finally, comparisons between PGM and OPGM are discussed in Section 3.4.

- In Section 4, a formal description of MLCP is presented in Section 4.1, followed by a simple example of algorithm implementation illustrated in Section 4.2. The performance of MLCP on synthetic and real-life networks are summarized and discussed in Section 4.3. Finally, further discussions are provided in Section 4.4 on some details of the simulations generating the results presented in Figure 4(c) in the main paper, as well as the insights we could achieve in this figure.

1.5 The List of Notations in this Supplementary Information

Table S1: **Notations in this Supplementary Information.** In the column 'Used in' of this list, 'T' is short for Theorem and 'L' is short for Lemma. 'All' means the symbol is used in almost every theorem and every lemma.

Symbol	Meaning	Used in	Symbol	Meaning	Used in
A	The adjacent matrix	All	B	The input matrix	All
E_A	Edge set of the network	L 1<br>T 1<br>T 2<br>T 3	$E(n_{wait})$	The expectation of the waiting steps	T 6
$\mathbb{E}(B)$	The energy cost function with the input matrix B	L 4<br>T 7	η	The step Length in OPGM	L 4<br>T 7
G	The static Bayesian game	T 3	$G(A, B)$	A linear control system or its corresponding digraph	L 1
$H(A)$	The corresponding bipartite graph of $G(A, B)$	L 2<br>T 3	k_i^{out}	The out-degree of v_i^+	T 3<br>T 5
k_j^{in}	The in-degree of v_j^-	T 3<br>T 5	$k_i^{out,n}$	The out-degree of v_i in the n-th iteration	T 5
$k_j^{in,n}$	The in-degree of v_j in the n-th iteration	T 5	$k_{v_m^{j,+}}^{out}$	The out-degree of Node $v_m^{j,+}$	T 3
$k_{v_m^{i,-}}^{in}$	The in-degree of Node $v_m^{i,-}$	T 3	$\bar{k}$	The mean degree	T 5
$\hat{\bar{k}}_{i,in}$, $\hat{\bar{k}}_{i,out}$	The ceiling function of the average input and output degrees of these adjacent nodes for node v_i^+ respectively	T 3	$k_{out,t}^{max}$	The maximum out-degree of the network in the t-th iteration	T 5

Continued on next page

Table S1 – continued from previous page

Symbol	Meaning	Used in	Symbol	Meaning	Used in
$k_{in,t}^{max}$	The maximum in-degree of the network in the t-th iteration	T 5	k_t^{max}	$k_t^{max} = max\{k_{out,t}^{max}, k_{in,t}^{max}\}$	T 5
L	The number of edges, in other words, L is the cardinality of the set E_A	T 5	N	The number of nodes, in other words, N is the cardinality of the set V_A	T 3<br>T 5
$Node$	The set of nodes in the digraph in game G, which consists of N nodes	T 3	$N(B)$	A function of the input matrix B; represent the boundary condition	L 3
M	The number of external control inputs	All	Ω	The set of states of nature, $\Omega = \{\omega_1, \omega_2, \omega_3\}$	T 3
p_i	Probability distribution over Ω for Node i in game G	T 3	p_{ma}	The probability that a node is matched	T 3
p_{un}	The expected probability that a node is not matched till the end	T 3	p_{ij}^n	The probability that node i and node j are matched in the n-th iteration	T 5
$p_{i,ap}(v_i^+ \xrightarrow{q-order} v_j^-)$	The probability that a q-order augmenting path about v_i^+ is formed when v_i^+ sends a child request to v_j^- and $(v_i^+ \to v_j^-)$ becomes matched	T 3<br>T 4	$\hat{p}^t$	The expected probability that the matchings are foremd in the t-th iteration	T 5
p^{in}	The in-degree distribution	T 4<br>T 5	p^{out}	The out-degree distribution	T 4<br>T 5
Q_t	$Q_t = k_{out,t}^{max} \cdot k_{in,t}^{max}$	T 5	S	Subset of V_A, $S \subseteq V_A$	L 1<br>T 1

Continued on next page

Table S1 – continued from previous page

Symbol	Meaning	Used in	Symbol	Meaning	Used in
S_i	The set of strategies of Node i in game G	T 3	$S(w)$	A function of w, $S(w) = \sum_{i=0}^{+\inf}(i+1)w^i$	T 6
Γ	Edges set of the bipartite graph, $\Gamma = \{(v_i^+ \to v_j^-) \| a_{ji} \neq 0\}$	L 2	t_i	The type of Node i in game G	T 3
t_{end}	The number of iterations of the LM algorithm	T 5	$T(S)$	Neighborhood set, $T(S) = \{x_j \| (x_j \to x_i) \in E_A, x_i \in S\}$	L 1<br>T 1
$u_i(t)$	The i-th time-dependent external control input	All	u_i	The payoff function of Node i in game G	T 3
v_i,<br>v_j	Elements of V_A, v_i, $v_j \in V_A$	All	v_i^+	Elements of V_A^+, $v_i^+ \in V_A^+$	All
v_j^-	Elements of V_A^-, $v_j^- \in V_A^-$	All	$v_x^{i,-}$	Elements of $V_{v_i^+}$	T 3
$v_y^{j,+}$	Elements of $V_{v_j^-}$	T 3	$V_{v_i^+}$	The set of adjacent nodes of v_i^+	T 3
$V_{v_j^-}$	The set of adjacent nodes of v_j^-	T 3	V_A	Node set of the network	All
V_A^+	The set of vertices corresponding to the N columns of the matrix A	All	V_A^-	The set of vertices corresponding to the N rows of the matrix A	All
w	The waiting probability	All	x_i,<br>x_j	Node in digraph $G(A, B)$	L 1
$x_i(t)$	The state of Node i at time t	All	ξ_{node}	The number of nodes left in the network in the final iteration	T 5

Continued on next page

Table S1 – continued from previous page

Symbol	Meaning	Used in	Symbol	Meaning	Used in
α_n, β_n, γ_n, δ_n	The expected number of nodes counted during calculating p_{ap}; detailed definition in the proof of Theorem 3	T 3	δ_i	The proportion of nodes with out-degree k_i^{out}	T 5
$\delta_{j\|i}$	The conditional probability that nodes with out-degree k_i^{out} have adjacent nodes with in-degree k_j^{in}	T 5	ρ_k	The normalization ration at k-th iteration in OPGM	T 7

2 Local-game Matching (LM)

The codes of the local-game matching algorithm have been already published on GitHub (available at https://github.com/PinkTwoP/Local-Game-Matching). As presented in the main paper, in the local-game matching, every node requests one of its neighbor nodes to become its parent node, and another one to become its child node, if applicable. When a node is seeking for a match, we define the number of its unmatched child (parent) nodes, i.e., the nodes that have not yet achieved a match with a parent (child), as its u-output (u-input) degree. Denote the u-input and u-output degrees of x_i as $N^{u-in}(x_i)$ and $N^{u-out}(x_i)$, respectively. Also denote the minimum u-input degree of x_i's unmatched child nodes as $\min\{N^{u-in}(x_i)\}$, and the minimum u-output degree of x_i's unmatched parent nodes as $\min\{N^{u-out}(x_i)\}$. The basic strategy is that, each node without a matched child (parent) node sends a child (parent) request to its neighboring child/parent node (including itself if there exists a self-loop link to the node) with the minimum u-input (u-output) degree. Figure S1 illustrates the flowchart of the LM algorithm containing the following three components:

1) *Child locating*: Each node without a matched child node sends a child request to its neighboring child node (including itself if there exists a self-loop link to the node) with the minimum u-input degree $\min\{N^{u-in}(x_i)\}$. When there is a tie, i.e., a node has multiple unmatched child nodes with the same minimum u-input degree, the node either holds on at a probability w or randomly breaks the tie.

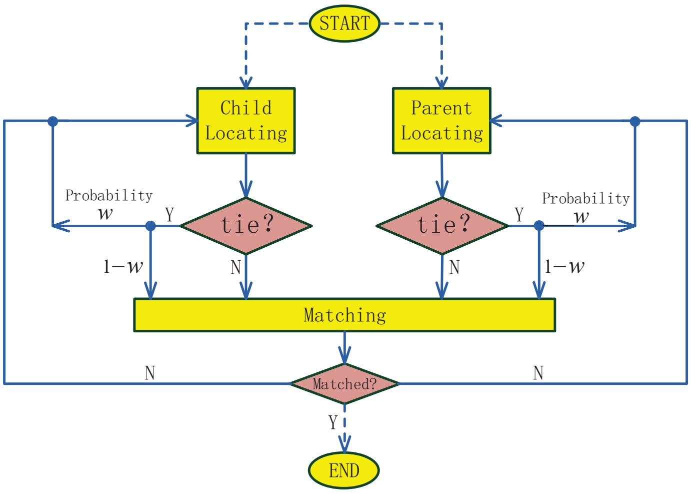

Figure S1: **Flowchart of implementing the LM algorithm.**

2) *Parent locating*: Each node without a matched parent node sends a parent request to the neighboring parent node (including itself if there exists a self-loop link to the node) with the minimum u-output degree $\min\{N^{u-out}(x_i)\}$. When there is a tie, i.e., a node has multiple unmatched parent nodes with the same minimum u-output degree, the node either holds on at a probability w or randomly breaks the tie.

3) *Matching*: When there is a match of requests, i.e., two nodes receive each other's parent/child request, a parent-child match is achieved and fixed. The child node in this match removes all its links to other parent nodes, and the u-output degrees of those parent nodes are reduced by 1. The parent node in this match removes all of its links to other child nodes, and the u-input degrees of those child nodes are reduced by 1.

The iterative request-matching operations in the LM algorithm continue until no more child/parent match can be achieved.

2.1 Examples of LM in small networks

We use two simple examples to illustrate the matching process of the LM algorithm. In the first example in Figure S2, it is interesting to observe that LM manages to reach the same final answer with the minimum number of driver nodes through a few different paths. Specifically, for node x_2, as x_5 and x_7 have the same number of input links, they have the equal chance to be selected as x_2's child node; and for node x_6, as x_5 and x_9 have the same number of output links, they have the equal chance to be selected as x_6's parent node. In LM, each node has a waiting probability w to hold on when there is a tie. As some edges may be removed in the iteration process, the tie may be broken. As illustrated in Figure S2, there

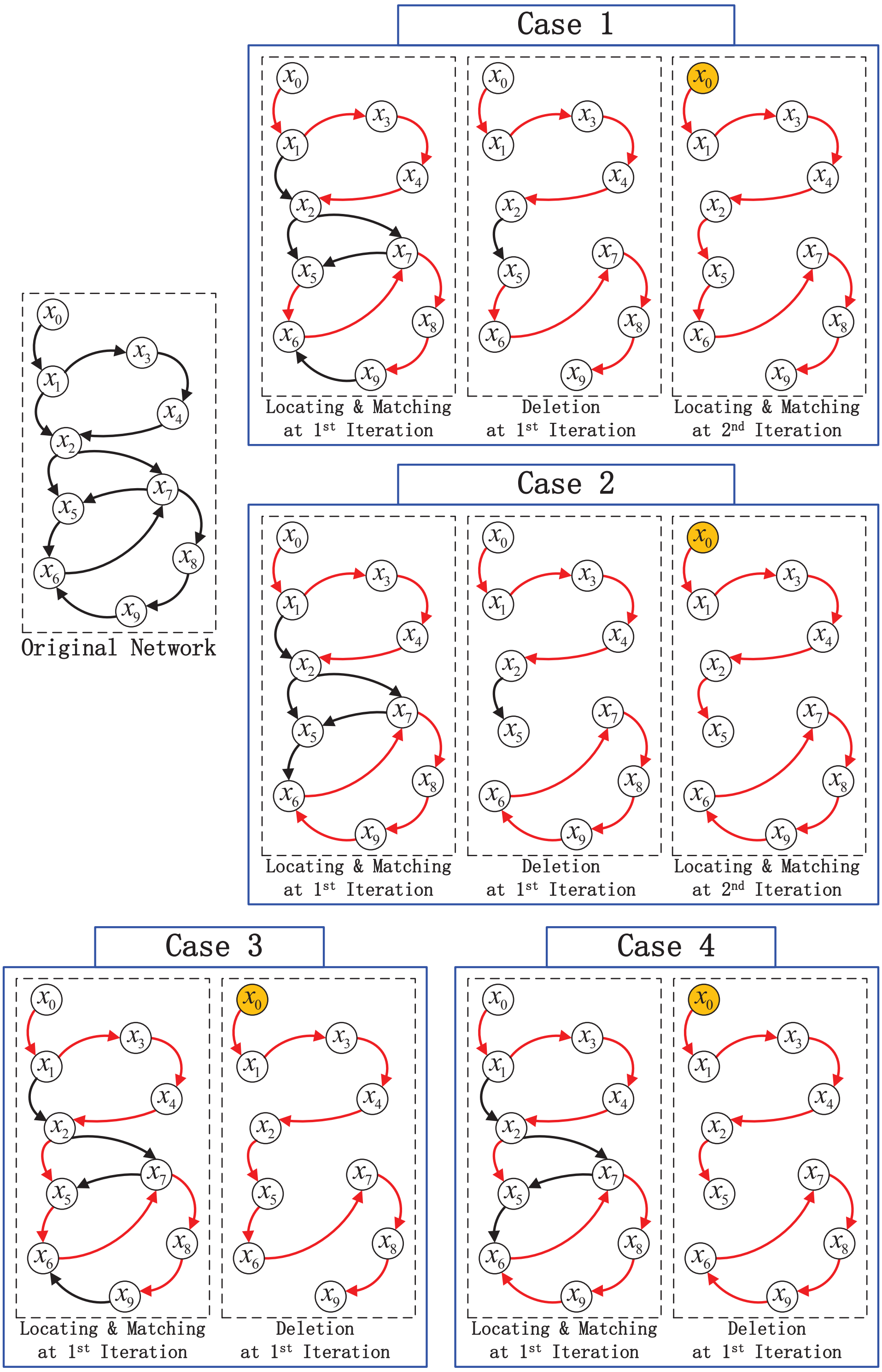

Figure S2: **An example of the LM method in a small network.** The nodes connected by dot lines are matched nodes and x_0 is the driver node determined by LM.

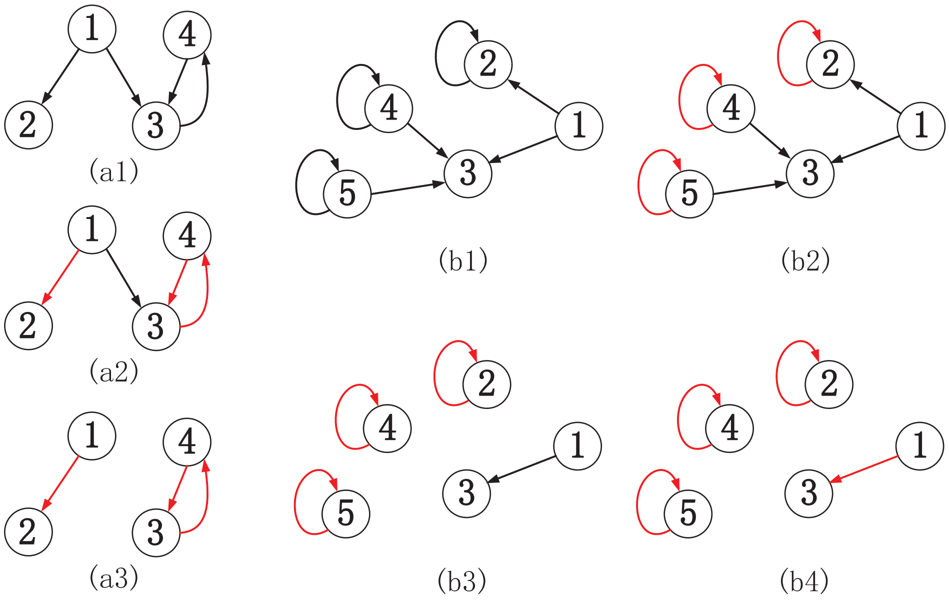

Figure S3: **A node x_i matches with another node which does not necessarily has** $\min\{N^{u-in}(x_i)\}$ **or** $\min\{N^{u-out}(x_i)\}$ **in its neighborhood in the original network.**

may be 4 different cases with different tie-breaking process, which however lead to the same number of driver nodes in this example. As will be discussed later in Section 2.7, different waiting processes may lead to different results, though the differences typically are not significant.

In the second example in Figure S3, we show that while a node x_i always sends a child/parent request to another node with $\min\{N^{u-in}(x_i)\}$ or $\min\{N^{u-out}(x_i)\}$, that does not necessarily mean that it will be finally matched with the node with $\min\{N^{u-in}(x_i)\}$ or $\min\{N^{u-out}(x_i)\}$ in the original network. As shown in Figure S3, each red arrow means a *matched edge* (i.e., an edge with its end nodes making a match) based on a bidirectional-choice rule. For example, in (a1), node 1 and node 2 form a parent-child match because node 1 requests node 2 as its child, and node 2 also requests node 1 as its parent. The whole matching process is simple and easy to understand. Figure S3(b) illustrates a different case. In this case, some nodes have self-loop links thus these nodes may send their parent/child requests to themselves. In Local-game matching, node 3 may request node 1 as its parent with a probability of 1/3, while node 1 will request node 2 to be its child. Meanwhile nodes 2, 4 and 5 each matches with itself in the first iteration. Finally node 1 matches with node 3, a node which does not have minimum u-input degree in node 1's neighborhood in the original network (b1). Note that the number of driver nodes obtains its minimum value, i.e., $N_D = 1$, in this example.

2.2 Theoretical Analysis of Local-game Matching

To facilitate discussions, hereafter we denote A as the adjacent matrix, V_A as the vertex set, and E_A as the edge set.

Lemma 1. *(Lin's Structural Controllability Theorem [1]). The following two statements are equivalent:*

1*) A linear control system $G(A, B)$ is structurally controllable.*

2*) The digraph $G(A, B)$ contains no inaccessible nodes or dilation.* □

Remarks:

1. A node x_i in the digraph $G(A, B)$ is called inaccessible iff there are no directed paths reaching x_i. Note that a node with a self-loop edge is an accessible node.

2. The digraph $G(A, B)$ contains a dilation iff there is a subset $S \subseteq V_A$ such that $|T(S)| < |S|$. Here, the neighborhood set $T(S)$ of a set S is defined as the set of all nodes x_j where there exists a directed edge from x_j to a node in S, i.e., $T(S) = \{x_j | (x_j \to x_i) \in E_A, x_i \in S\}$. $|S|$ and $|T(S)|$ are the cardinality of set S and $T(S)$, respectively.

□

Theorem 1. *Structural controllability of a complex network can be ensured by applying the LM algorithm.*

Proof. We prove by contradiction. Assume that the complex network is not structurally controlled after using the LM algorithm. Then there must exist inaccessible nodes and dilations. When there exist inaccessible dilations, from the definition of dilation, we have $\exists\ S \subseteq V_A,\ |T(S)| < |S|$. In other words, $\exists\ v_j \in T(S)$ and $v_i \in S,\ (v_j \to v_i) \in E_A$. Since $\exists\ (v_j \to v_i) \subseteq E_A$, we have that v_j lacks child and v_i lacks parent. Otherwise, if v_j already has a child or v_i already has a parent, $(v_j \to v_i)$ should have been removed according to the LM algorithm. With the link $(v_j \to v_i)$ still being there, the LM algorithm can continue, as v_j can send a child request to v_i and v_i can send a parent request to v_j. Figure S4 shows a simple example. This leads to a contradiction. Hence we prove that the LM algorithm can continue untile there is no dilation left.

For inaccessible nodes, they can be controlled by assigning new external control inputs to them which makes them become driver nodes.

From above we have the conclusion that there is no inaccessible node or dilation after implementing the LM algorithm. From Lemma 1, we have that the network is structurally controlled. There are two

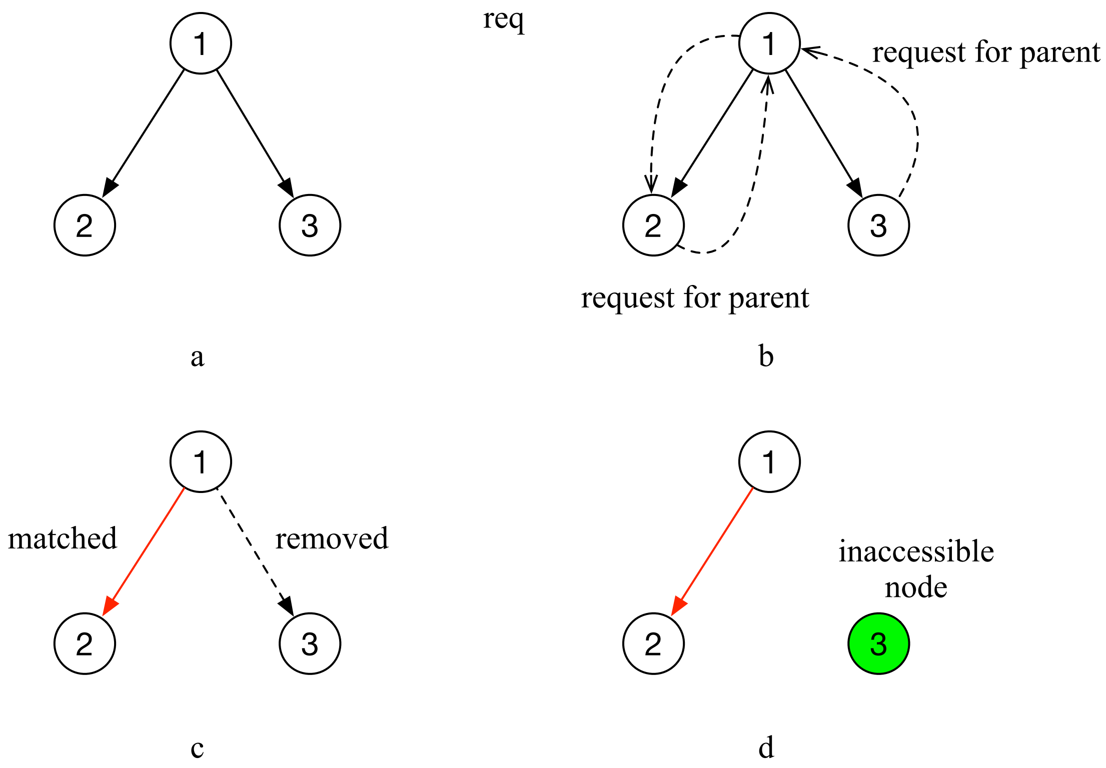

Figure S4: **Proof illustration of Theorem 1**. (a) There are some dilations left in the network, which are labeled as Node 1, Node 2 and Node 3. Node 1 lacks child while Node 2 and Node 3 lack parent. (b) Node 2 and Node 3 send parent requests to Node 1. Node 1 randomly chooses one of Node 2 and Node 3 to send a child request. In this example, Node 1 chooses Node 2. (c) Node 1 and Node 2 are matched, consequently the directed edge between Node 1 and Node 3 is removed. (d) After one step of the LM algorithm, one match and one inaccessible node are formed.

types of control paths in a complex network after implementing the LM algorithm: directed control paths (DCPs), the origin of each of which is an unmatched node which is taken as a driver node; and circled control paths (CCPs), in which every node has a child and a parent. □

Figure S5 illustrates an example, from which we can see that there are only DCPs and CCPs left in the network after implementing the LM algorithm. By connecting external control inputs to the origins of DCPs, the network can be structurally controlled. Note that for the CCP, a randomly selected node needs to be connected to an existing external controller.

Definition [19]. For any directed complex network, its digraph can be represented in a corresponding bipartite graph denoted as $H(A)$. The bipartite graph is defined as $H(A) = (V_A^+ \cup V_A^-, \Gamma)$, where $V_A^+ = \{v_1^+, v_2^+, ..., v_N^+\}$ and $V_A^- = \{v_1^-.v_2^-, ..., v_N^-\}$ are the set of vertexes corresponding to the N columns and rows of A respectively, and edges set $\Gamma = \{(v_i^+ \to v_j^-)|a_{ji} \neq 0\}$.

Figure S6 shows an example of the above definition.

We have that an augmenting path in $H(A)$ has the properties as follows [6]:

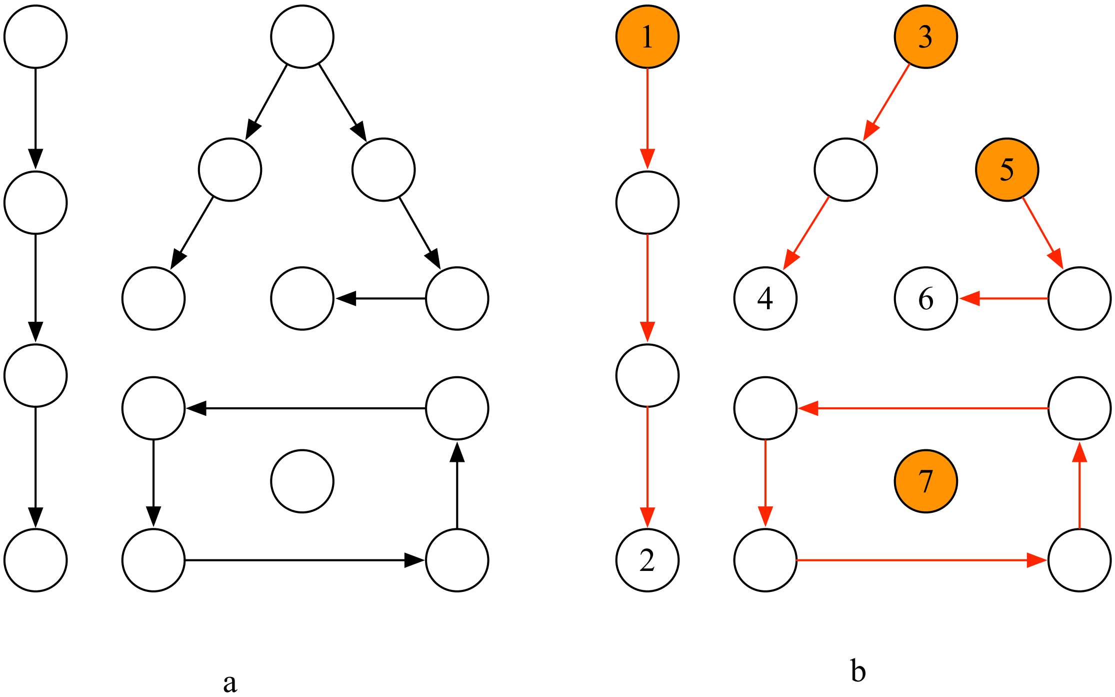

Figure S5: **An example of network after implementing the LM algorithm**. (a) The network has 15 nodes with two elementary stems, an elementary dilation and an elementary circle. (b) After implementing the LM algorithm, Node 1, Node 3, Node 5 and Node 7 are the origins of four directed control paths respectively. By connecting external control inputs to these four nodes (which are colored in orange), the network can be structurally controlled. For the elementary circle, a randomly selected node needs to be connected to an existing external control input.

(1) It has an odd number of edges;

(2) Its origin and end are not matched;

(3) Its origin and end are on the different sides of $H(A)$;

(4) Its edges are alternatively unmatched (at least one endpoint of the edge is not matched) and matched (both two endpoints of the edge are matched);

(5) By reversing the matching of the edges in the augmenting path (set the unmatched edges to be matched and set the matched edges to be unmatched), the number of matchings will be increased by one.

In Figure S6, $(v_3^+ \to v_2^-)$, $(v_1^+ \to v_2^-)$ and $(v_1^+ \to v_1^-)$ form an augmenting path, if only $(v_1^+ \to v_2^-)$ is matched.

Theorem 2. *Implementing the LM algorithm on digraph of a complex network is equivalent to applying it on the corresponding bipartite graph.*

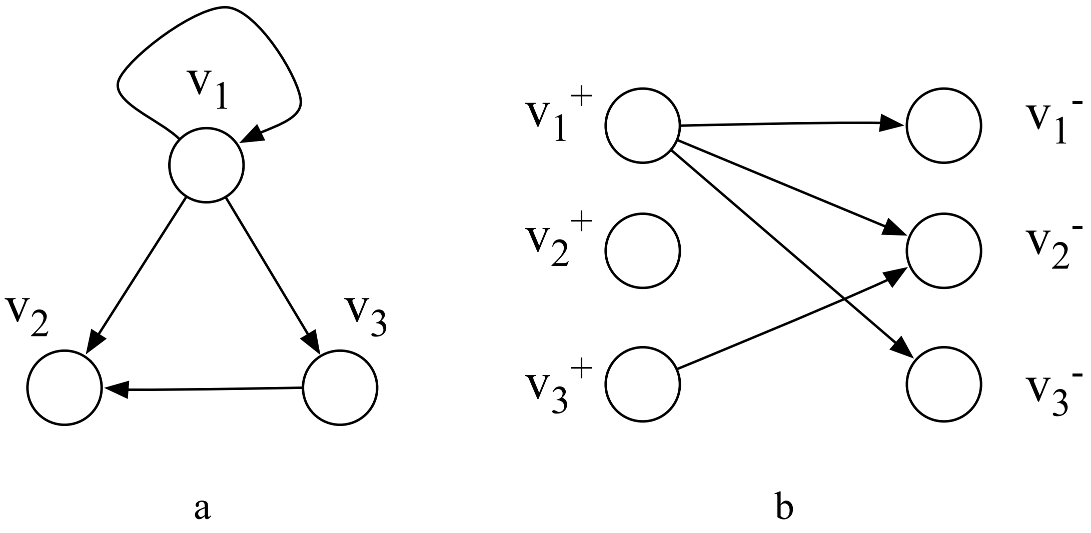

Figure S6: **An example of bipartite graph construction**. (a) The digraph of a network with three nodes and four edges. (b) the corresponding bipartite graph $H(A)$ with six nodes and four edges.

Proof. In digraph, the LM algorithm lets each node send a parent (child) request to its neighbor node with the minimum out-degree (in-degree); such remains unchanged in the corresponding bipartite graph. Furthermore, in digraph where a match is identified between v_i and v_j, the LM algorithm removes other edges of v_i and v_j. Such would ensure that, in the corresponding bipartite graph, a matched node will not be matched with any other nodes, which makes the matching in the LM algorithm to be equivalent to the matching in the corresponding bipartite graph.

To make it easier to understand, we describe the LM algorithm in the bipartite graph as follows:

Step (1) Each node in V_A^+ sends a child request to its adjacent node in V_A^- with the minimum in-degree, while each node in V_A^- sends a parent request to its adjacent node in V_A^+ with the minimum out-degree;

Step (2) Iff v_i^+ sends a request to v_j^- while v_j^- sends a request to v_i^+ in step (1), would v_i^+ and v_j^- form up a match;

Step (3) If v_i^+ and v_j^- form up a match in step (2), remove all the other edges connected to v_i^+ and v_j^-; this ensures that these two nodes will not send requests to any other nodes;

Step (4) Repeat step (1) to step (3), until no further matches can be formed.

□

We define an augmenting path with q matched edges, $(q+1)$ unmatched edges and v_i^+ being the second node along the path as a q-order augmenting path about v_i^+. We also define $p_{i,ap}(v_i^+ \xrightarrow{q-order} v_x^{i,-})$ as

the probability that a q-order augmenting path about v_i^+ is formed when node v_i^+ sends a child request to node $v_x^{i,-}$ and $(v_i^+ \to v_x^{i,-})$ becomes matched. We have the following Theorem.

Theorem 3. *Assume that $0 \leq p_{un} \leq 1$ ($p_{ma} = 1 - p_{un}$) is the expected probability that a node is unmatched till the end of the LM algorithm. We have that $p_{i,ap}(v_i^+ \xrightarrow{q-order} v_j^-)$ can be approximated as*

$$\hat{p}_{i,ap}(v_i^+ \xrightarrow{q-order} v_j^-) = \left(1 - p_{ma}^{(k_j^{in}-1)\cdot\sum_{m=0}^{q-1}\prod_{n=1}^{m}\alpha_{n+1}}\right) \cdot \left(1 - p_{ma}^{(k_i^{out}-1)\cdot\sum_{m=0}^{q-1}\prod_{n=1}^{m}\beta_{n+1}}\right), \quad (1)$$

where v_j^- is one of v_i^+'s child nodes with an input degree of k_j^{in}. The detailed definition of α_n and β_n is in the proof. By letting each node send a child/parent request to its adjacent nodes with the minimum input/output degrees, the LM algorithm minimizes $\hat{p}_{i,ap}(v_i^+ \xrightarrow{q-order} v_j^-)$ and $\hat{p}_{j,ap}(v_i^+ \xrightarrow{q-order} v_j^-)$, regardless of whether there exist ties (defined in Section 1.2) or not.

Proof. According to Theorem 2, hereafter we prove Theorem 3 in the corresponding bipartite graph.

Randomly observe $(v_i^+ \to v_j^-) \in H(A)$, which corresponds to $(v_i \to v_j) \in E_A$. We assume that v_i^+ has k_i^{out} outgoing edges and v_j^- has k_j^{in} incoming edges. The adjacent nodes of v_i^+ form the set $V_{v_i^+}$; and the adjacent nodes of v_j^- form the set $V_{v_j^-}$. In other words,

$$V_{v_i^+} = \{v_x^{i,-} | x = 1, 2, ..., k_i^{out}\}, \; V_{v_j^-} = \{v_y^{j,+} | y = 1, 2, ..., k_j^{in}\}.$$

Figure S7 shows the connection between v_i^+ and v_j^-.

Now we assume that there is at least one node in $\complement_{V_{v_i^+}}\{v_j^-\}$ ($\complement_{V_{v_i^+}}\{v_j^-\}$ is a complement operation, which is a node set comprising of nodes in $V_{v_i^+}$ except v_j^-) that is not matched until the end of the algorithm. When there is at least one node in $\complement_{V_{v_j^-}}\{v_i^+\}$ (similarly, $\complement_{V_{v_j^-}}\{v_i^+\}$ is a set comprising of nodes in $V_{v_j^-}$ except v_i^+) unmatched till the end, the two unmatched nodes, two unmatched edges and matched $(v_i^+ \to v_j^-)$ form an augmenting path. This happens at a probability of $(1 - p_{ma}^{k_j^{in}-1})$. Otherwise, if all nodes in $\complement_{V_{v_j^-}}\{v_i^+\}$ are matched to some nodes while at least one of these nodes has at least one unmatched adjacent node till the end, the two unmatched nodes, three unmatched edges and two matched edges also form an augmenting path. This happens at an expected probability of $p_{ma}^{k_j^{in}-1} \cdot \left(1 - p_{ma}^{(k_j^{in}-1)\cdot\alpha_2}\right)$, where α_2 is the expected number of nodes in $\complement_{V_{v_y^{j,+}}}\{v_j^-\}$ ($\forall\, v_y^{j,+} \in \complement_{V_{v_j^-}}\{v_i^+\}$) which have no connections with the anterior part (1-order path) of the augmenting path (viz. node v_i^+). Note that some nodes may have the same unmatched adjacent nodes. Each of the overlapped nodes should be counted once only. This is illustrated in Figure S8(b). In Figure S8(b), the blue dashed line means v_k^- has one connection with v_i^+ which is already on the augmenting path. And the green dashed line means v_k^- and $v_p^{z,-}$ have the same unmatched node v_s^+ which is the overlapped part. When calculating the probability that all nodes

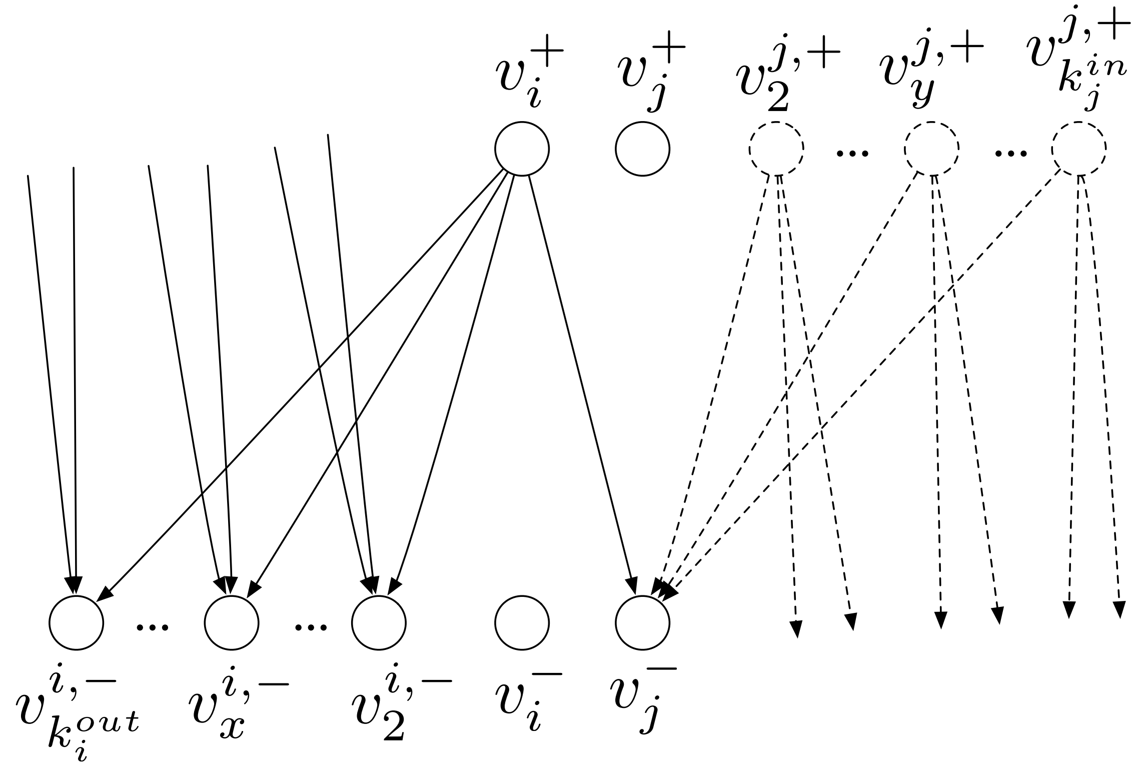

Figure S7: ($v_i^+ \to v_j^-$). On the left are the adjacent nodes of v_i^+ and their respective incoming links, plotted in solid lines; and on the right are adjacent nodes of v_j^- and their respective outgoing links, plotted in dotted lines. In this case in the picture, v_j^- is $v_1^{i,-}$ and v_i^+ is $v_1^{j,+}$.

in $\mathtt{C}_{V_{v_j^-}}\{v_i^+\}$ are matched to some nodes while at least one of these nodes has at least one unmatched adjacent node till the end, those nodes in V_A^+ with blue and green dashed lines should be ignored.

Further moving forward, we may similarly denote set $\mathcal{C}_2$ as $\mathtt{C}_{V_{v_y^{j,+}}}\{v_j^-\}$ ($\forall\ v_y^{j,+} \in \mathtt{C}_{V_{v_j^-}}\{v_i^+\}$). If all nodes in $\mathtt{C}_{\mathcal{C}_2}\{v_y^{j,+}\}$ are matched to some nodes of which all adjacent nodes are matched to some nodes which have at least one unmatched adjacent node till the end, the two unmatched nodes, four unmatched edges and three matched edges form an augmenting path. The expected probability that this happens equals $p_{ma}^{k_j^{in}-1} \cdot p_{ma}^{(k_j^{in}-1)\cdot\alpha_2} \cdot \left(1 - p_{ma}^{(k_j^{in}-1)\cdot\alpha_2\cdot\alpha_3}\right)$, where α_3 is the expected number of nodes in $\mathtt{C}_{v_s^+}\{\mathcal{C}_2\}$ which have no connections with the anterior part (from 1-order path to 2-order path) of the augmenting path (viz. node v_i^+ and node $v_y^{j,+}$) and with the overlapped part being ignored.

To generalize, the augmenting path in Figure S8(a) is 1-order augmenting path about v_i^+ and the augmenting path in Figure S8(b) is a 2-order augmenting path about v_i^+. Thus, if we consider the q-order augmenting path about v_i^+, the probability that this augmenting path exists is

$$\prod_{m=0}^{q-2} p_{ma}^{(k_j^{in}-1)\cdot\prod_{n=1}^{m}\alpha_{n+1}} \cdot \left(1 - p_{ma}^{(k_j^{in}-1)\cdot\prod_{n=2}^{q}\alpha_n}\right).$$

Figure S8 illustrates the process.

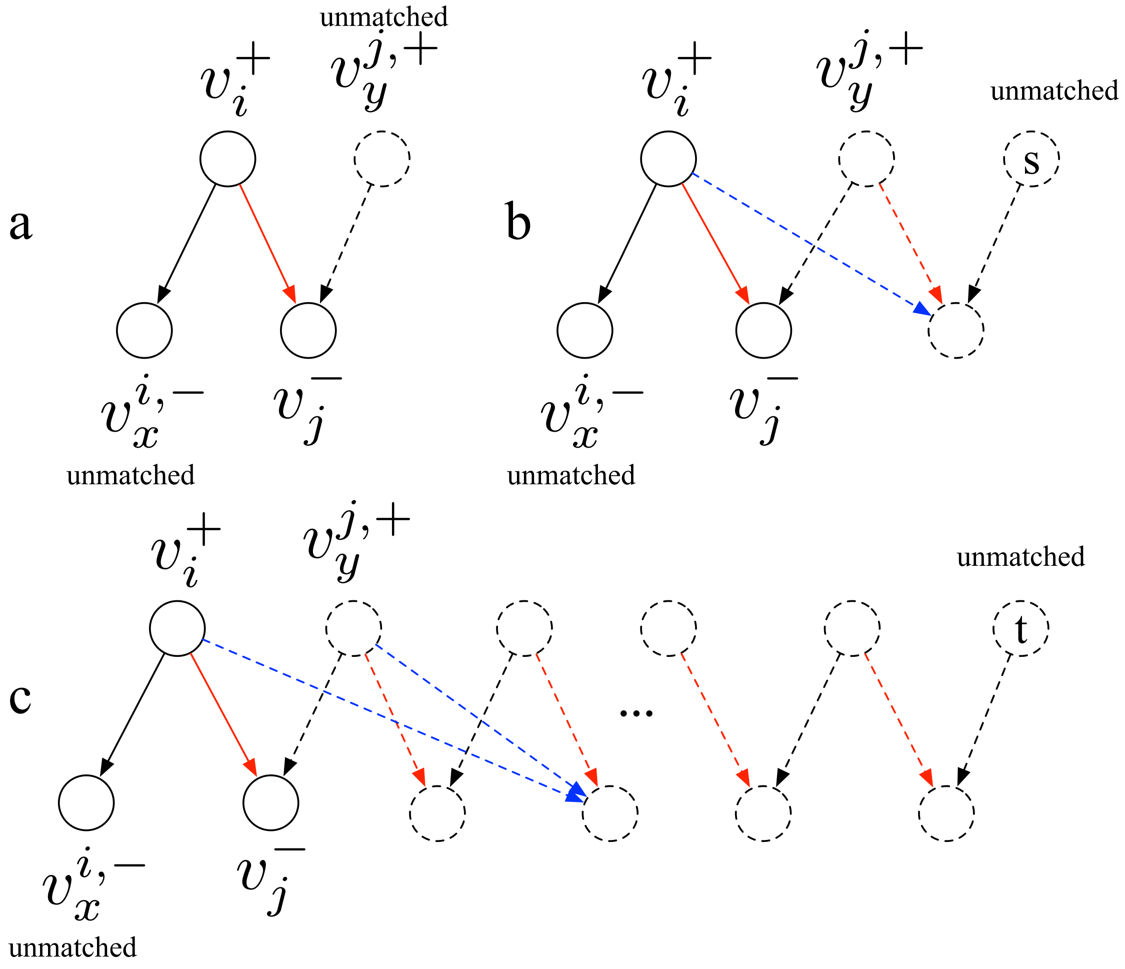

Figure S8: **Estimating the probability that an augmenting path is formed**. Red lines represent matched edges. (a) If $v_x^{i,-}$ and $v_y^{j,+}$ are not matched till the end, an augmenting path is formed. (b) If $v_y^{j,+}$ is matched but node s is unmatched till the end, the augmenting path is also formed. (c) To generalize, if node t is unmatched till the end, an augmenting path is formed.

Therefore, based on the assumption that there is at least one node in $\complement_{V_{v_i^+}}\{v_j^-\}$ not matched till the end, we have the probability that an augmenting path is formed if $(v_i^+ \to v_j^-)$ becomes a match, which is

$$
\begin{aligned}
&1 - p_{ma}^{k_j^{in}-1} + p_{ma}^{k_j^{in}-1} \cdot (1 - p_{ma}^{(k_j^{in}-1)\alpha_2} + p_{ma}^{(k_j^{in}-1)\alpha_2} \cdot (1 - p_{ma}^{(k_j^{in}-1)\alpha_2\alpha_3}) \\
&+ \ldots + p_{ma}^{(k_j^{in}-1)\alpha_2\alpha_3} \cdot \ldots \cdot (1 - p_{ma}^{(k_j^{in}-1)\prod_{n=2}^{q}\alpha_n})\ldots)) \\
=&1 - \prod_{m=0}^{q-1} p_{ma}^{(k_j^{in}-1)\cdot\prod_{n=1}^{m}\alpha_{n+1}} \\
=&1 - p_{ma}^{(k_j^{in}-1)\cdot\sum_{m=0}^{q-1}\prod_{n=1}^{m}\alpha_{n+1}}.
\end{aligned}
\tag{2}
$$

Back to the assumption that there is at least one node in $\complement_{V_{v_i^+}}\{v_j^-\}$ not matched till the end, similarly the probability that an augmenting path about v_j^- exists can be calculated as $\left(1 - p_{ma}^{(k_i^{out}-1)\cdot\sum_{m=0}^{q-1}\prod_{n=1}^{m}\beta_{n+1}}\right)$, where β_n has analogous meaning with α_n. Thus the expected probability that an augmenting path is formed if v_i^+ sends a child request to v_j^- and $(v_i^+ \to v_j^-)$ becomes matched is

$$\hat{p}_{i,ap}(v_i^+ \xrightarrow{q-order} v_j^-) = \left(1 - p_{ma}^{(k_j^{in}-1)\cdot\sum_{m=0}^{q-1}\prod_{n=1}^{m}\alpha_{n+1}}\right) \cdot \left(1 - p_{ma}^{(k_i^{out}-1)\cdot\sum_{m=0}^{q-1}\prod_{n=1}^{m}\beta_{n+1}}\right). \quad (3)$$

Now we can also calculate the expected probability $\hat{p}_{j,ap}(v_i^+ \xrightarrow{q-order} v_j^-)$ that an augmenting path would be formed if v_j^- sends a parent request to v_i^+ and $(v_i^+ \to v_j^-)$ becomes matched. The result is similar to that for $\hat{p}_{i,ap}(v_i^+ \xrightarrow{q-order} v_j^-)$. Specifically, we have

$$\hat{p}_{j,ap}(v_i^+ \xrightarrow{q-order} v_j^-) = \left(1 - p_{ma}^{(k_j^{in}-1)\cdot\sum_{m=0}^{q-1}\prod_{n=1}^{m}\delta_{n+1}}\right) \cdot \left(1 - p_{ma}^{(k_i^{out}-1)\cdot\sum_{m=0}^{q-1}\prod_{n=1}^{m}\gamma_{n+1}}\right), \quad (4)$$

where γ_{n+1} and δ_{n+1} have the analogous meanings with α_{n+1} and $\beta n + 1$ respectively.

Denote $v_x^{i,-}$ as the node with the minimum in-degree in $V_{v_i^+}$, and its in-degree as k_{min}^{in}. As the LM algorithm makes v_i^+ send a child request to $v_x^{i,-}$, we have that the expected probability that an augmenting path is formed if v_i^+ sends a child request to $v_x^{i,-}$ and $(v_i^+ \to v_x^{i,-})$ become matched equals

$$\hat{p}_{i,ap}(v_i^+ \xrightarrow{q-order} v_x^{i,-}) = \left(1 - p_{ma}^{(k_{min}^{in}-1)\cdot\sum_{m=0}^{q-1}\prod_{n=1}^{m}\alpha_{n+1}}\right) \cdot \left(1 - p_{ma}^{(k_i^{out}-1)\cdot\sum_{m=0}^{q-1}\prod_{n=1}^{m}\beta_{n+1}}\right). \quad (5)$$

Since $\hat{p}_{i,ap}(v_i^+ \xrightarrow{q-order} v_x^{i,-})$ is monotonically increasing with k_j^{in}, it reaches the minimum value when $k_j^{in} = k_{min}^{in}$.

For v_j^-, similarly we have that $\hat{p}_{j,ap}$ is minimized as LM algorithm makes v_j^- send a parent request to its adjacent node with the minimal output degree.

Further consider the case where v_i^+ has h adjacent nodes with the same minimal input degree k_{min}^{in} (viz. there exists a tie), $\hat{p}_{i,ap}(v_i^+ \xrightarrow{q-order} v_x^{i,-})$ does not change its form. Thus the strategy that v_i^+ sends a child request to one of its adjacent nodes with the minimal input degree still minimizes $\hat{p}_{i,ap}(v_i^+ \xrightarrow{q-order} v_x^{i,-})$. □

Based on Theorem 3, we can further prove that the LM algorithm allows the system to reach the equilibrium of a static Bayesian game.

Theorem 4. *Given only local information (defined in Section 1.2), the LM algorithm is equivalent to a static Bayesian game. And a Nash equilibrium of the game exists, which is the solution of the LM algorithm.*

Proof. Based on Theorem 2, hereafter we prove Theorem 4 in the corresponding bipartite graph, i.e. LM algorithm guides all the nodes to make its best choice with only local information.

Since the LM algorithm uses only the local information to allow all network nodes to send requests to their selected neighbor nodes simultaneously in every iteration, the structural control problem of complex network with only local information can be viewed as a static game with incomplete information, also known as the static Bayesian game [20]. Specifically, the structural control problem to which LM algorithm is applied can be translated into the game defined as $G = \langle Node,\ \Omega,\ \langle S_i,\ u_i,\ t_i,\ p_i \rangle \rangle$, where

(1) $Node$ is the set of nodes in the digraph consisting of N nodes;

(2) Ω is the set of states of nature (Here "nature" can be interpreted as the consensus of all nodes. Further discussions will be provided later in this section.). Specifically, $\Omega = \{\omega_1,\ \omega_2,\ \omega_3\}$, where ω_1 is that not to be on an augmenting path is most important, ω_2 is that to be matched is very important, and ω_3 is that every node knows that only local information is available to every node in the network;

(3) t_i is the type of Node i. Specifically, t_i defines that Node i is in V_A^+ or V_A^-;

(4) S_i is the set of strategies of Node i, which is to request for child or parent to nodes in the neighborhood of Node i based on the type t_i of Node i.

For v_i^+, its type t_i is that Node i is in V_A^+. Thus we have

$$S_i(t_i = \{v_i^+\}) = \{s_{ix} : s_{ix}\ means\ v_i^+\ sends\ a\ child\ request\ to\ its\ adjacent\ node\ v_x^{i,-}, \\ x = 1, 2, ..., k_i^{out}\}. \tag{6}$$

For v_j^-, its type t_j is that Node j is in V_A^-. Thus we have

$$S_j(t_j = \{v_j^-\}) = \{s_{jy} : s_{jy}\ means\ v_j^-\ sends\ a\ parent\ request\ to\ its\ adjacent\ node\ v_y^{j,+}, \\ y = 1, 2, ..., k_j^{in}\}; \tag{7}$$

(5) $u_i : \Omega \times S \to R$ is the payoff function of Node i, i.e., the payoff of Node i to choose strategies from S_i based on the nature of Ω. The payoff of Node i is only related to the Bayesian game result, and every node is only able to estimate the expected payoff.

For v_i^+, its type t_i is that Node i is in V_A^+.

$$u_i(t_i = \{v_i^+\}) = \begin{cases} 1, & v_i^+\ is\ matched\ and\ not\ on\ an\ augmenting\ path \\ 0, & v_i^+\ is\ not\ mathced\ or\ on\ an\ augmenting\ path \end{cases}. \tag{8}$$

For v_j^-, its type t_j is that Node j is in V_A^-.

$$u_j(t_j = \{v_j^-\}) = \begin{cases} 1, & v_j^- \textit{ is matched and not on an augmenting path} \\ 0, & v_j^- \textit{ is not matched or on an augmenting path} \end{cases}. \quad (9)$$

(6) p_i is probability distribution over Ω for Node i (viz. p_i is the probability that Node i chooses strategy S_i); different nodes have different p_i according to their local information based on the nature of Ω.

The strategy chosen by Node i is based on its payoff function u_i, which is influenced by the strategy chosen by its neighbors. An equilibrium of the game G is defined to be a Nash equilibrium of the game $\hat{G} = \langle Node, \hat{S} = S_1 \times S_2 \times \cdots \times S_N, \hat{u} = u \rangle$. For the structual control problem of complex network with finite nodes, the equivalent game equilibrium always exists [20].

Before solving the equilibrium problem, we provide some further explanations on the "nature" to make it easier to understand. We assume that in the game G, every node is in all the states of Ω. ω_1 means that every node is egoless to reduce the number of independent external control inputs required, as having an augmenting path means that there is one more matching available which could reduce one external control input. ω_2 means that every node is selfish and anxious to be matched as far as such is possible. ω_3 means that while making a decision, every node knows that limited local information is all what the other nodes can obtain.

To prove that the LM algorithm is the equilibrium, we need to show that in the game, considering the strategies of other nodes, every node can maximize its expected payoff by sending child (parent) request to its adjacent node with minimal input (output) degree. We firstly consider the probability $\hat{p}_{i,ap}(v_i^+ \to v_x^{i,-})$ that v_i^+ is on a q-order augmenting path if v_i^+ chooses v_j^- to request for child. From Equations (3) and (4), we have that $\hat{p}_{i,ap}(v_i^+ \xrightarrow{q-order} v_j^-)$ ($\hat{p}_{j,ap}(v_i^+ \xrightarrow{q-order} v_j^-)$) is monotone increasing with k_j^{in} (k_i^{out}), which means v_i^+ (v_j^-) should choose $v_x^{i,-}$ ($v_y^{j,+}$) with the minimal in-degree (out-degree) to request for child (parent) in order to minimize the probability of forming up an augmenting path. Therefore, no matter which type Node i is, nature ω_1 encourages Node i to choose its neighbor node with the minimal in-degree (out-degree) to request for child (parent). This preferred strategic decision of $v_x^{i,-}$ can be inferred by v_i^+ because of nature ω_3: to maximize the probability that v_i^+ is matched, v_i^+ shall firstly estimate the probability each node in $V_{v_i^+}$ will request for parent to it by estimating the probability it has the minimal out-degree among all the adjacent nodes of $v_x^{i,-}$.

Subject to the limitation of having only local information, v_i^+ cannot find out what exactly the out-degree distribution in adjacent neighbor of $v_x^{i,-}$ or in the global network is. Thus v_i^+ may assume that the other nodes in adjacent neighbor of $v_x^{i,-}$ have an out-degree distribution denoted as $\hat{p}^{out}$. Therefore the probability that v_i^+ has the minimal out-degree in adjacent neighbor of $v_x^{i,-}$ is

$\left(1-\sum_{s=0}^{k_i^{out}} \hat{p}^{out}(K=s)\right)^{k^{in}_{v_x^{i,-}}-1}$, where $k^{in}_{v_x^{i,-}}$ is in-degree of $v_x^{i,-}$. If we take into consideration the situation that there are h adjacent nodes with the same minimal out-degree (viz. the tie exists) and $v_x^{i,-}$ would randomly choose one from these h nodes to request for parent, the probability can be revised as

$$\left(1-\sum_{s=0}^{k_i^{out}} \hat{p}^{out}(K=s)\right)^{k^{in}_{v_x^{i,-}}-h} /h,$$

denoted as $\hat{p}(v_x^{i,-} \xrightarrow[parent]{request} v_i^+)$.

As $\hat{p}(v_x^{i,-} \xrightarrow[parent]{request} v_i^+)$ is monotone increasing with $k^{in}_{v_x^{i,-}}$, which means nodes with larger in-degrees are less likely to request for parent to v_i^+, v_i^+ shall choose the adjacent node with the minimal in-degree to request for child to maximize the probability of making a match. Similarly, when there are h nodes with the same minimal in-degree, v_j^- can estimate the probability that $v_y^{j,+}$ requests for child to it as

$$\left(1-\sum_{s=0}^{k_j^{in}} \hat{p}^{in}(K=s)\right)^{k^{out}_{v_y^{j,+}}-h} /h.$$

Thus, v_j^- shall choose the node with the minimal out-degree to request for parent in order to maximize the probability of making a match.

To summarize, by sending child (parent) request to the adjacent node with the minimal input (output) degree, every node can maximize the probability that it is matched and not on an augmenting path. In the game, every node wants to maximize its expected payoff, which mathematically equals the probability that it is matched and not on an augmenting path. In other words, when Node i selects an adjacent node to send a request, it firstly minimizes the probability that it is on an augmenting path, and then maximizes the probability that it is matched. The two goals can be achieved simultaneously when Node i sends a request to the adjacent node with the minimal degree. This is the equilibrium of the static Bayesian game.

Because game G is static where all nodes make strategic decisions simultaneously in each iteration, we have the probability distribution over Ω for Node i as

$$p_i(S_i(t_i=\{v_i^+\})=\{s_{ix}\})=\begin{cases} 1/h, & v_x^{i,-} \; and\ other\ (h-1)\ nodes\ have\ the\ minimal\ in-degree \\ 0, & otherwise \end{cases}, \tag{10}$$

and

$$p_i(S_i(t_i = \{v_i^-\}) = \{s_{iy}\}) = \begin{cases} 1/h, & v_y^{i,+} \textit{ and other } (h-1) \textit{ nodes have the minimal out} - \textit{degree} \\ 0, & \textit{otherwise} \end{cases} . \quad (11)$$

Therefore, the equilibrium of game G is that every node randomly chooses one from those nodes with the minimal degree among its immediate neighbors. LM algorithm guides each node to make its best choice with the limited local information. □

For a network with its in-degree and out-degree distributions being independent of network size N, denote $p^{in}(s)$ as the fraction of nodes with in-degree s, $s = i^{in}_{\min}, i^{in}_{\min}+1, \ldots, i^{in}_{\max}$, where $i^{in}_{\min}$ and $i^{in}_{\max}$ denote the minimum and maximum in-degree of the network nodes, respectively. Similarly define $p^{out}(s)$ as the fraction of nodes with out-degree s, $s = i^{out}_{\min}, i^{out}_{\min}+1, \ldots, i^{out}_{\max}$. We have the following theorem.

Theorem 5. *For a network with its in-degree and out-degree distributions being independent of the size of the network, the average time complexity of the LM algorithm is $O(N)$.*

Proof. Consider a network with N nodes and in-degree/out-degree distributions p^{in} and p^{out}, respectively. To simplify the proof, we start with the case where each node has only a single adjacent node with the minimum in-degree or out-degree.

In the first iteration of the LM algorithm, as each node sends a request to its adjacent node with the minimal in-degree (or out-degree), the probability p_{ij} that node i and node j are matched is decided by the probability that node i has the minimal out-degree among neighbors of node j and the probability that node j has the minimal in-degree among neighbors of node i. Thus we have that the matching probability equals

$$p_{ij} = \left(1 - \sum_{s=0}^{k_i^{out}} p^{out}(s)\right)^{k_j^{in}-1} \left(1 - \sum_{s=0}^{k_j^{in}} p^{in}(s)\right)^{k_i^{out}-1},$$

where k_i^{out} and k_j^{in} are the out-degree of node i and in-degree of node j respectively.

Let the matched nodes be ignored while calculating degree distribution in the following iterations, or in other words, they may be regarded as getting removed. Denote $p^{in,t}$ and $p^{out,t}$ as the in-degree and out-degree distributions of the network in the t-th iteration, respectively. We have

$$p_{xy}^t = \left(1 - \sum_{s=0}^{k_x^{out,t}} p^{out,t}(s)\right)^{k_y^{in,t}-1} \left(1 - \sum_{s=0}^{k_y^{in,t}} p^{in,t}(s)\right)^{k_x^{out,t}-1}.$$

Figure S9 illustrates an example.

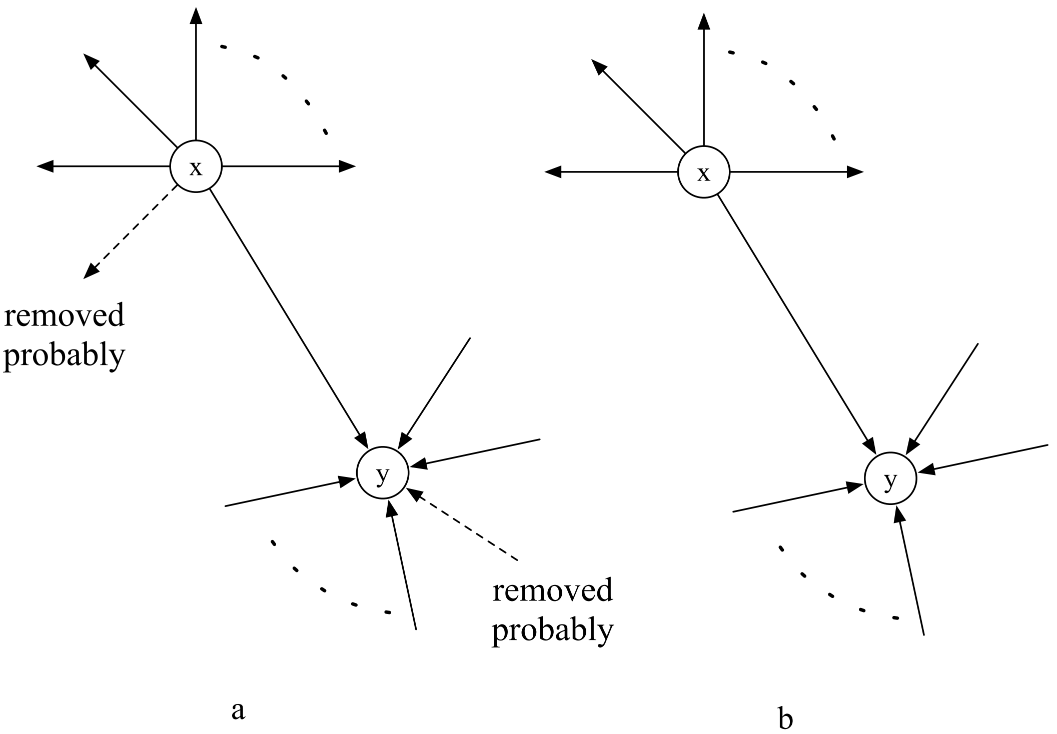

Figure S9: **An example of the probable changes in the local region of v_x and v_y.** (a) In the local region of v_x and v_y, edges may be removed in the previous iterations because nodes at the other end of the edges have made matches. (b) After edge removals, k_x^{out} and k_y^{in} decrease.

Now we extend to the case where node v_x has z_x adjacent nodes with the same minimal in-degree and node v_y has z_y adjacent nodes with the same minimal out-degree. Node v_x shall thus have a probability w to hold on, and a probability $(1-w)$ to randomly choose one from the z_x adjacent nodes with the minimal in-degree to send a request. The probability that v_x sends a child request to v_y thus can be calculated as

$$\sum_{z_x=1}^{k_i^{out}} (1-w)\frac{1}{z_x}\binom{k_i^{out}-1}{z_x-1}\left(1-\sum_{s=0}^{k_j^{in}} p^{in}(s)\right)^{k_i^{out}-z_x} \cdot p^{in}(k_j^{in})^{z_x-1}.$$

Applying the binomial theorem, we have

$$\begin{aligned}
&\sum_{z_x=1}^{k_i^{out}} (1-w)\frac{1}{z_x}\binom{k_i^{out}-1}{z_x-1}\left(1-\sum_{s=0}^{k_j^{in}} p^{in}(s)\right)^{k_i^{out}-z_x} \cdot p^{in}(k_j^{in})^{z_x-1} \\
=&(1-w)\sum_{z_x=1}^{k_i^{out}} \frac{1}{z_x}\frac{(k_i^{out}-1)!}{(z_x-1)!(k_i^{out}-z_x)!}\left(1-\sum_{s=0}^{k_j^{in}} p^{in}(s)\right)^{k_i^{out}-z_x} \cdot \frac{p^{in}(k_j^{in})^{z_x}}{p^{in}(k_j^{in})} \\
=&\frac{1-w}{p^{in}(k_j^{in})}\sum_{z_x=1}^{k_i^{out}} \frac{1}{k_i^{out}}\frac{k_i^{out}!}{z_x!(k_i^{out}-z_x)!}\left(1-\sum_{s=0}^{k_j^{in}} p^{in}(s)\right)^{k_i^{out}-z_x} \cdot p^{in}(k_j^{in})^{z_x} \\
=&\frac{1-w}{k_i^{out}\cdot p^{in}(k_j^{in})}\sum_{z_x=1}^{k_i^{out}} \binom{k_i^{out}}{z_x}\left(1-\sum_{s=0}^{k_j^{in}} p^{in}(s)\right)^{k_i^{out}-z_x} \cdot p^{in}(k_j^{in})^{z_x} \\
=&\frac{1-w}{k_i^{out}\cdot p^{in}(k_j^{in})}\cdot\left(\left(1-\sum_{s=0}^{k_j^{in}} p^{in}(s)+p^{in}(k_j^{in})\right)^{k_i^{out}} - \left(1-\sum_{s=0}^{k_j^{in}} p^{in}(s)\right)^{k_i^{out}}\right) \\
=&\frac{1-w}{k_i^{out}\cdot p^{in}(k_j^{in})}\cdot\left(\left(1-\sum_{s=0}^{k_j^{in}-1} p^{in}(s))\right)^{k_i^{out}} - \left(1-\sum_{s=0}^{k_j^{in}} p^{in}(s)\right)^{k_i^{out}}\right).
\end{aligned}$$

Similarly, for v_y we that have the probability that v_y chooses v_x to request for parent is

$$\frac{1-w}{k_j^{in}\cdot p^{out}(k_i^{out})}\cdot\left(\left(1-\sum_{s=0}^{k_i^{out}-1} p^{out}(s))\right)^{k_j^{in}} - \left(1-\sum_{s=0}^{k_i^{out}} p^{out}(s)\right)^{k_j^{in}}\right).$$

Therefore, for pair $(v_x \to v_y)$, the probability that $(v_x \to v_y)$ is matched is

$$\begin{aligned}
p_{ij} =&\frac{(1-w)^2}{k_i^{out}k_j^{in}\cdot p^{in}(k_j^{in})\cdot p^{out}(k_i^{out})}\cdot\left(\left(1-\sum_{s=0}^{k_j^{in}-1} p^{in}(s))\right)^{k_i^{out}} - \left(1-\sum_{s=0}^{k_j^{in}} p^{in}(s)\right)^{k_i^{out}}\right) \\
&\cdot\left(\left(1-\sum_{s=0}^{k_i^{out}-1} p^{out}(s))\right)^{k_j^{in}} - \left(1-\sum_{s=0}^{k_i^{out}} p^{out}(s)\right)^{k_j^{in}}\right).
\end{aligned}$$

For the t-th iteration,

$$\begin{aligned}
p_{ij}^t =& \frac{(1-w)^2}{k_i^{out,t} k_j^{in,t} \cdot p^{in,t}(k_j^{in}) \cdot p^{out,t}(k_i^{out})} \\
& \cdot \left(\left(1 - \sum_{s=0}^{k_j^{in,t}-1} p^{in,t}(s)) \right)^{k_i^{out,t}} - \left(1 - \sum_{s=0}^{k_j^{in,t}} p^{in,t}(s) \right)^{k_i^{out,t}} \right) \\
& \cdot \left(\left(1 - \sum_{s=0}^{k_i^{out,t}-1} p^{out,t}(s)) \right)^{k_j^{in,t}} - \left(1 - \sum_{s=0}^{k_i^{out,t}} p^{out,t}(s) \right)^{k_j^{in,t}} \right).
\end{aligned}$$

Since

$$\begin{aligned}
& \sum_{z_x=1}^{k_i^{out}} (1-w) \frac{1}{z_x} \binom{k_i^{out}-1}{z_x-1} \left(1 - \sum_{s=0}^{k_j^{in}} p^{in}(s) \right)^{k_i^{out}-z_x} \cdot p^{in}(k_j^{in})^{z_x-1} \\
\geq & \sum_{z_x=k_i^{out}}^{k_i^{out}} (1-w) \frac{1}{z_x} \binom{k_i^{out}-1}{z_x-1} \left(1 - \sum_{s=0}^{k_j^{in}} p^{in}(s) \right)^{k_i^{out}-z_x} \cdot p^{in}(k_j^{in})^{z_x-1} \\
\geq & (1-w) \frac{1}{k_i^{out}} \cdot p^{in}(k_j^{in})^{k_i^{out}-1},
\end{aligned}$$

we have

$$\begin{aligned}
p_{ij}^t =& \frac{(1-w)^2}{k_i^{out,t} k_j^{in,t} \cdot p^{in,t}(k_j^{in}) \cdot p^{out,t}(k_i^{out})} \\
& \cdot \left(\left(1 - \sum_{s=0}^{k_j^{in,t}-1} p^{in,t}(s)) \right)^{k_i^{out,t}} - \left(1 - \sum_{s=0}^{k_j^{in,t}} p^{in,t}(s) \right)^{k_i^{out,t}} \right) \\
& \cdot \left(\left(1 - \sum_{s=0}^{k_i^{out,t}-1} p^{out,t}(s)) \right)^{k_j^{in,t}} - \left(1 - \sum_{s=0}^{k_i^{out,t}} p^{out,t}(s) \right)^{k_j^{in,t}} \right) \\
\geq & \frac{(1-w)^2}{k_i^{out,t} k_j^{in,t}} p^{in,t}(k_j^{in,t})^{k_i^{out,t}-1} p^{out,t}(k_i^{out,t})^{k_j^{in,t}-1}
\end{aligned}$$

For the whole network, the anticipant probability $\hat{p}^t$ of match making is $\sum_i \sum_j \delta_i \delta_{j|i} \cdot p_{ij}^t$, where δ_i is the proportion of nodes with out-degree k_i^{out} in the network, i.e., $p^{out}(k_i^{out})$, and $\delta_{j|i}$ is the conditional

probability that nodes with out-degree k_i^{out} have adjacent nodes with in-degree k_j^{in}. Hence we have

$$
\begin{aligned}
\hat{p}^t =& \sum_i \sum_j \left(\delta_i \delta_{j|i} \cdot p_{ij}^t\right) \\
=& \sum_i \sum_j p^{out,t}(k_i^{out,t}) \cdot \left(1 - \left(1 - p^{in,t}(k_j^{in,t})\right)^{k_i^{out,t}}\right) \cdot \frac{(1-w)^2}{k_i^{out,t} k_j^{in,t}} p^{in,t}(k_j^{in,t})^{k_i^{out,t}-1} p^{out,t}(k_i^{out,t})^{k_j^{in,t}-1} \\
=& \sum_i \sum_j \cdot \left(1 - \left(1 - p^{in,t}(k_j^{in,t})\right)^{k_i^{out,t}}\right) \cdot \frac{(1-w)^2}{k_i^{out,t} k_j^{in,t}} p^{in,t}(k_j^{in,t})^{k_i^{out,t}-1} p^{out,t}(k_i^{out,t})^{k_j^{in,t}} \\
\geq& \sum_i \sum_j \cdot \left(1 - \left(1 - p^{in,t}(k_j^{in,t})\right)^{k_i^{out,t}}\right) \cdot \frac{(1-w)^2}{k_i^{out,t} k_j^{in,t}} p^{in,t}(k_j^{in,t})^{k_i^{out,t}-1} p^{out,t}(k_i^{out,t})^{k_{\max}^{in,t}} \\
\geq& \sum_i \sum_j \cdot \left(1 - \left(1 - p^{in,t}(k_j^{in,t})\right)^{1}\right) \cdot \frac{(1-w)^2}{k_i^{out,t} k_j^{in,t}} p^{in,t}(k_j^{in,t})^{k_i^{out,t}-1} p^{out,t}(k_i^{out,t})^{k_{\max}^{in,t}} \\
=& \sum_i \sum_j \cdot \frac{(1-w)^2}{k_i^{out,t} k_j^{in,t}} p^{in,t}(k_j^{in,t})^{k_i^{out,t}} p^{out,t}(k_i^{out,t})^{k_{\max}^{in,t}} \\
\geq& \sum_i \sum_j \cdot \frac{(1-w)^2}{k_i^{out,t} k_j^{in,t}} p^{in,t}(k_j^{in,t})^{k_{\max}^{out,t}} p^{out,t}(k_i^{out,t})^{k_{\max}^{in,t}} \\
\geq& \sum_i \sum_j \cdot \frac{(1-w)^2}{k_{\max}^{out,t} k_{\max}^{in,t}} p^{in,t}(k_j^{in,t})^{k_{\max}^{out,t}} p^{out,t}(k_i^{out,t})^{k_{\max}^{in,t}} \\
=& \frac{(1-w)^2}{k_{\max}^{out,t} k_{\max}^{in,t}} \sum_i \sum_j p^{in,t}(k_j^{in,t})^{k_{\max}^{out,t}} p^{out,t}(k_i^{out,t})^{k_{\max}^{in,t}} \\
\geq& \frac{(1-w)^2}{k_{\max}^{out,t} k_{\max}^{in,t}} \sum_i \sum_j \left(p^{in,t}(k_j^{in,t}) p^{out,t}(k_i^{out,t})\right)^{k_t^{\max}}
\end{aligned}
$$

where $k_{max}^{out,t}$ and $k_{max}^{in,t}$ are the maximum out-degree and maximum in-degree of the network in the t-th iteration respectively, and $k_{\max}^t = \max\{k_{\max}^{out,t}, k_{\max}^{in,t}\}$.

As $\sum_{i=0}^{k_{\max}^{out,t}} \sum_{j=0}^{k_{max}^{in,t}} p^{in,t}(k_j^{in,t}) p^{out,t}(k_i^{out,t}) = 1$, denoting P_{ij} as $p^{in,t}(k_j^{in,t}) p^{out,t}(k_i^{out,t})$ and Q_t as $k_{\max}^{out,t} \cdot k_{\max}^{in,t}$, we have $\sum_{i,j}^{Q_t} P_{ij} = 1$. From Hölder inequality, we have

$$
\begin{aligned}
\left(\sum_{i,j}^{Q_t} P_{ij}^{k_{\max}^t}\right)^{\frac{1}{k_{\max}^t}} \cdot \left(\sum_{i,j}^{Q_t} 1\right)^{1-\frac{1}{k_{\max}^t}} &\geq \sum_{i,j}^{Q_t} P_{ij} \cdot 1 = 1. \\
\Rightarrow \sum_{i,j}^{Q_t} P_{ij}^{k_{\max}^t} \geq \left(\sum_{i,j}^{Q_t} 1\right)^{1-k_{\max}^t} &= Q_t^{1-k_{\max}^t}
\end{aligned}
$$

Therefore

$$
\begin{aligned}
\hat{p}^t \geq& \frac{(1-w)^2}{k_{\max}^{out,t} k_{\max}^{in,t}} \sum_i \sum_j \left(p^{in,t}(k_j^{in,t}) p^{out,t}(k_i^{out,t})\right)^{k_{\max}^t} \\
\geq& \frac{(1-w)^2}{k_{\max}^{out,t} k_{\max}^{in,t}} Q_t^{1-k_{\max}^t} = \frac{(1-w)^2}{Q^{k_{\max}^t}} = \hat{p}^{*,t} \geq p^{*,1}.
\end{aligned}
\tag{12}
$$

From Equation (12), $\hat{p}^{*,t}$ monotonically increases when $k_{\max}^{out,t}$ and $k_{\max}^{in,t}$ decrease. As the LM algorithm removes edges in every iteration , $k_{\max}^{out,t}$ and $k_{\max}^{in,t}$ monotonically decrease with an increasing value of t. Thus $\hat{p}^{*,t}$ has its minimum value when $t = 1$. Denote $\hat{p}^{*,1}$ as $\hat{p}^*$. We have that $\hat{p}^*$ is independent of the network size according to the theorem assumption, and $\hat{p}^*$ is strictly positive.

Denote the number of iterations until the algorithm stops as t_{end} and assume that, at the end of the $(t_{end} - 1)$-th iteration, there are ξ_{node} nodes left in the network ($\xi_{node} \in (0, N]$), which cannot make any further matches in the t_{end}-th iteration. We have

$$\left(\prod_{m=1}^{t_{end}-1} (1 - \hat{p}^m) \right) \cdot N = \xi_{node}.$$

As $\hat{p}^m \geq \hat{p}^*$ for $m = 1, 2, \ldots, t_{end} - 1$, we have that

$$(1 - \hat{p}^*)^{t_{end}-1} \geq \xi_{node}/N$$

and hence the expected number of iterations is capped at

$$t_{end} \leq \log_{(1-\hat{p}^*)}(\xi_{node}/N) + 1.$$

For convenience of discussion, hereafter we term this upper bound for the number of iterations as t_{end}.

Note that for every node seeking a match in each iteration, at most two operations are needed, namely to send out a parent and a child requests respectively. In the first iteration, $\hat{p}^1 \cdot N$ nodes get matched at a cost of $2 \cdot N$ operations. In the second iteration, averagely $\hat{p}^2(1 - \hat{p}^1) \cdot N$ nodes get matched, after $(1 - \hat{p}^1) \cdot 2 \cdot N$ operations. In the t-th iteration ($t > 1$), there are $\prod_{m=1}^{t-1}(1 - \hat{p}^m) \cdot 2 \cdot N$ operations. In the last iteration, there are ξ_{node} nodes left after $(2 \cdot \xi_{node})$ operations. Therefore, there will be a total of

$$2 \cdot N + \sum_{t=2}^{t_{end}-1} (\prod_{m=1}^{t-1} (1 - \hat{p}^m)) \cdot 2 \cdot N + 2 \cdot \xi_{node}$$

operations.

For simplicity, it can be derived that the number of operations has an upper bound as follows:

$$
\begin{aligned}
& 2 \cdot N + \sum_{t=2}^{t_{end}-1} (\prod_{m=1}^{t-1} (1-\hat{p}^m)) \cdot 2 \cdot N + 2 \cdot \xi_{node} \\
\leq & 2 \cdot N + \sum_{t=2}^{t_{end}-1} (1-\hat{p}^*)^{t-1} \cdot 2 \cdot N + 2 \cdot \xi_{node} \\
= & 2 \cdot N \cdot \sum_{t=1}^{t_{end}-1} (1-\hat{p}^*)^{t-1} + 2 \cdot \xi_{node} \\
= & 2 \cdot N \cdot \frac{1-(1-\hat{p}^*)^{t_{end}-1}}{1-(1-\hat{p}^*)} + 2 \cdot \xi_{node} \\
= & 2 \cdot N \cdot \frac{1-(1-\hat{p}^*)^{log_{(1-\hat{p}^*)}(\xi_{node}/N)}}{1-(1-\hat{p}^*)} + 2 \cdot \xi_{node} \\
= & 2 \cdot N \cdot \frac{1-\xi_{node}/N}{\hat{p}^*} + 2 \cdot \xi_{node} \\
= & 2 \cdot N \cdot \left(\frac{1}{\hat{p}^*} - \frac{\xi_{node}/N}{\hat{p}^*} + \xi_{node}/N \right) \\
= & 2 \cdot N \cdot \left(\frac{1}{\hat{p}^*} - \left(\frac{1}{\hat{p}^*} - 1 \right) \cdot \xi_{node}/N \right) \\
< & 2 \cdot N \cdot \frac{1}{\hat{p}^*}
\end{aligned}
$$

Thus we have that the total number of operations is bounded by $\frac{2 \cdot N}{\hat{p}^*}$. As $\hat{p}^*$ is independent of the size of the network, the average time complexity of LM algorithm is $O(N)$. □

From Theorem 5, we see that the LM algorithm has a linear average time complexity. For a given w, the expected number of iterations needed is independent of the network size, but related to network nodal degree distribution.

2.3 Definitions of the networks with other distributions

Let $f(x)$ be the probability density function of the nodal degree distribution in the network. In this contribution we add to the popular ER and B-A random networks a few more cases in which network nodal degrees follow the Chi-squared distribution, the Weibull distribution, and the Gamma distribution, respectively. These distribution functions are widely used in inferential statistics.

The probability density function $f(x)$ of the Chi-squared distribution is defined as [21]

$$
f(x, \mu) = \begin{cases} \frac{x^{(\mu/2)-1} e^{-x/2}}{2^{\mu/2}\Gamma(\frac{\mu}{2})}, & \text{if } x \geq 0; \\ 0, & \text{otherwise,} \end{cases} \tag{13}
$$

where μ is the freedom degree parameter and $\Gamma(\frac{\mu}{2})$ is the Gamma function that has closed-form values for integer μ. The Chi-squared distribution is also known as the Chi-square or χ^2 distribution.

The probability density function $f(x)$ of the Weibull distribution [22] is

$$f(x;\mu,k)=\begin{cases}\frac{k}{\mu}(\frac{x}{\mu})^{k-1}e^{-(x/\mu)^k}, & \text{if} \quad x\geq 0;\\ 0, & \text{otherwise,}\end{cases} \tag{14}$$

where $k>0$ is the shape parameter and $\mu>0$ is the scale parameter of the distribution. The Weibull distribution is related to a number of other probability distributions. In particular, it interpolates between the exponential distribution ($k=1$) and the Rayleigh distribution ($k=2$). For convenience, we let $k=1$ in this contribution.

The Gamma distribution [23] belongs to a family of two-parameter continuous probability distributions. Specifically, the probability density function $f(x)$ using the shape-scale parametrization is given by

$$f(x,k,\theta)=\frac{x^{k-1}e^{-\frac{x}{\theta}}}{\theta^k\Gamma(k)} \quad \text{for } x>0 \text{ and } k,\theta>0. \tag{15}$$

Here k and θ are the shape and scale parameters respectively, and $\Gamma(k)$ is the Gamma function evaluated at k.

2.4 Performance of Local-game Matching

We test the LM method on synthetic and real-life networks. The synthetic networks include the ER model [24], the BA network [25] [26], and networks generated using Chi-squared [21], Weibull [22] and Gamma [23] distributions, respectively. Topology information about all the real-life networks we tested are available from open sources (see the reference citations in Table S3). Note that both the LM and MM methods have been tested using Monte Carlo simulations. Specifically, on each of 10 independently generated synthetic networks we carry out 10 simulation rounds. Because the error bars of the results are very small, they are not included in the figures. Since random tie-breaking in real-life networks has little effect on the final results measured by the number of driver nodes, we present the results from only a single round of simulation.

We denote $n_D^{LM}=\frac{N_D^{LM}}{N}$ and $n_D=\frac{N_D}{N}$ as the respective percentages that the driver nodes found by LM and MM account for in the whole network, and denote μ the mean in-/out degree of the network (termed as *mean degree* in this report). We use the absolute error between LM and MM, Error $=n_D^{LM}-n_D$, to measure the performance.

Figure S10(a) shows the percentage of driver nodes identified by the LM and MM methods in the synthetic networks with different average nodal degrees. Note that the MM method achieves the optimal

Table S2: **The absolute errors between LM and MM in synthetic networks (**$N = 10000$**. The values in brackets show** n_D^{LM} **with** $w = \frac{2}{3}$**).**

Mean	E-R	B-A	Chi-squared	Weibull	Gamma
$\mu = 2$	0.46% (0.3246)	0.29% (0.3762)	0.16% (0.3803)	0.16% (0.3760)	0.18% (0.4673)
$\mu = 3$	1.23% (0.1740)	0.76% (0.2500)	0.79% (0.2246)	0.39% (0.2780)	0.15% (0.3183)
$\mu = 4$	0.73% (0.0963)	1.16% (0.1568)	0.66% (0.1323)	0.25% (0.2203)	0.20% (0.2246)
$\mu = 5$	0.22% (0.0556)	0.53% (0.0747)	0.52% (0.0830)	0.30% (0.1810)	0.27% (0.1540)
$\mu = 6$	0.06% (0.0256)	0.36% (0.0144)	0.18% (0.0326)	0.16%(0.1640)	0.13% (0.1026)
$\mu = 7$	0.00% (0.0143)	0.35% (0.0073)	0.03% (0.0240)	0.21% (0.1406)	0.02% (0.0663)
$\mu = 8$	0.00% (0.0066)	0.23% (0.0039)	0.00% (0.0113)	0.00% (0.1173)	0.01% (0.0516)
$\mu = 9$	0.00% (0.0056)	0.07% (0.0015)	0.00% (0.0093)	0.1140 (0.1146)	0.00% (0.0300)
$\mu = 10$	0.06% (0.0030)	0.01% (0.0004)	0.00% (0.0033)	0.03% (0.0883)	0.03% (0.0180)

solution. Also note that, utilizing only the local information, the LM method steadily achieves suboptimal solutions in terms of the number of driver nodes. Table S2 summarizes the calculation results produced by the two methods, and shows that the error of the LM method is typically $\ll 1\%$.

Figures S10(b) and S10(c) show that the two methods produce approximately the same number of driver nodes and that they also identify the nodes with approximately the same input and output degree distributions. Thus the two methods either find approximately the same set of nodes, or find two sets of nodes with approximately the same statistical properties. The sub-optimality of the LM method in these synthetic networks is thus verified.

We test the performance of the LM method in a number of real-life networks and the results are summarized in Table S3. Data of the topologies of these networks is freely available in the cited references. Note that the number of driver nodes identified by the LM method are consistently to be close to or equal to the optimal solutions identified by the MM method.

2.5 Empirical estimation of the number of iteration steps

Simulation results verify that the number of iterations is independent of network size. Figure S11 shows the LM method in three ER networks with the same mean nodal degree $\mu = 10$ but with sizes $N = 1000, N = 2000$, and $N = 3000$. The number of matched nodes at each iteration is plotted. Note that the number of iterations required is independent of N and that the calculations converge after $\mathbf{k} = 12$ iterations in all the three cases. This occurs because LM is a local information-based decentralized method

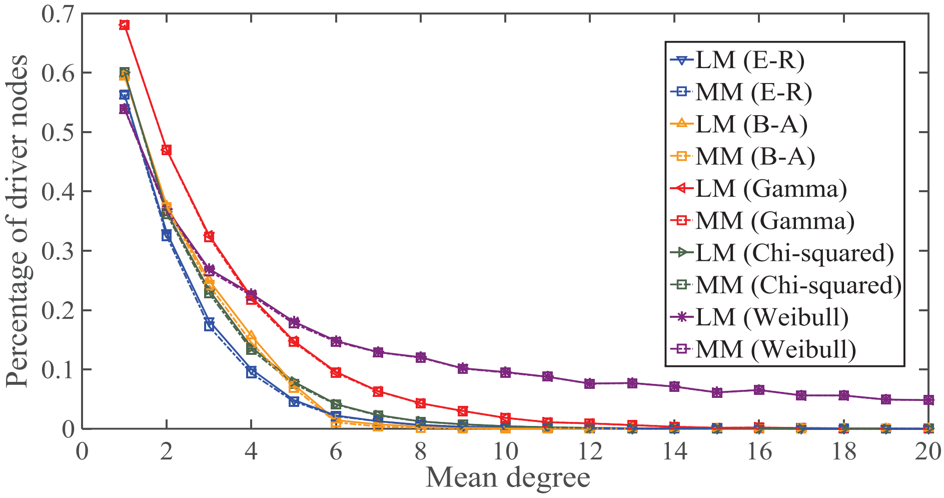

(a)

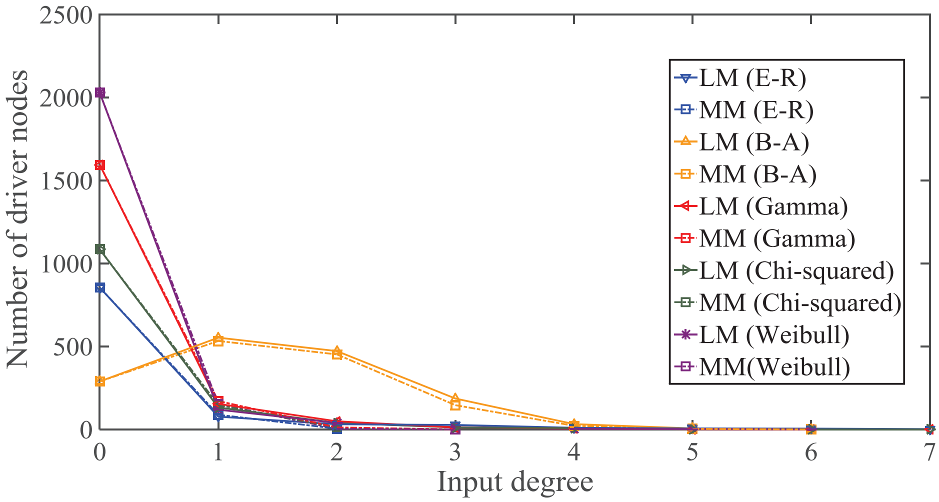

(b)

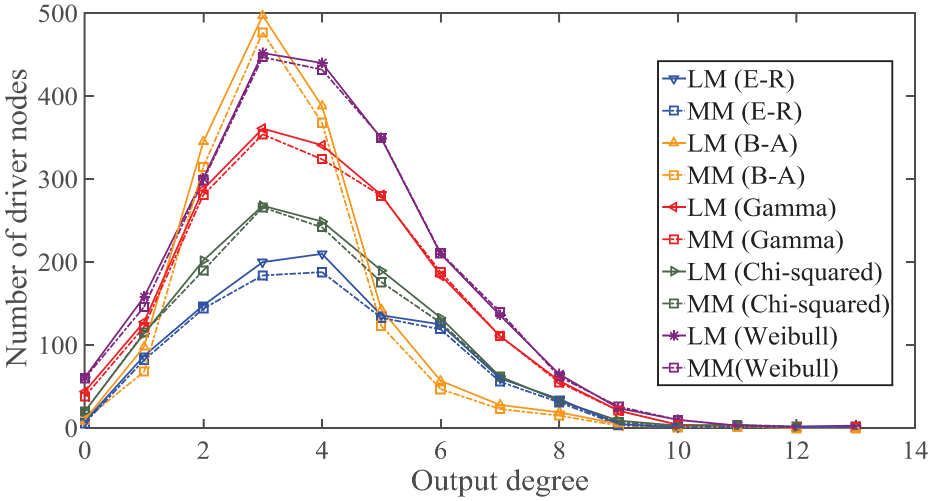

(c)

Figure S10: **LM and MM in synthetic networks: (a)** Number of driver nodes with respect to the mean degree μ for ER, B-A and other networks for LM and MM (N = 10000). **(b)** Input nodal degree distributions of the driver nodes found by LM and MM, respectively ($N = 10000$ and $\mu = 6$). **(c)** Output degree nodal degree distributions of the driver nodes found by LM and MM, respectively.

Table S3: **The absolute errors between LM and MM in real-life networks ($^*: w = \frac{2}{3}$; $^{**}: w = 0.85$; and $w = 0$ for the others.)**

Data Set	Network	Nodes	Edges	N_D	N_D^{LM}	Error
Airports [27, 28]	us*	1574	28236	581	588	0.44%
	intl*	2939	30501	872	890	0.61%
	airports500*	500	5960	125	128	0.60%
Web [29–31]	problogs*	1224	19025	436	443	0.47%
	web-BerkStan*	685230	7600595	260229	265157	0.72%
	web-Google*	875713	5105039	422608	425153	0.27%
	web-Stanford*	281903	2312497	89346	91702	0.83%
Electronic Circuit [32]	circuit-s208	122	189	29	29	0.00%
	circuit-s420	252	399	59	59	0.00%
	circuit-s838	512	819	119	119	0.00%
Food Web [33–37]	CrystalC	24	125	10	10	0.00%
	Florida	128	2106	30	30	0.00%
	Maspalomas	30	87	9	9	0.00%
	Rhode	25	58	8	8	0.00%
	Michigan	39	221	13	13	0.00%
	baydry	128	2137	29	29	0.00%
Gnutella P2P [38, 39]	p2p-Gnutella04*	10876	39994	4640	4700	0.55%
	p2p-Gnutella05	8846	31839	5111	5112	0.01%
	p2p-Gnutella08	6301	20777	4105	4106	0.02%
	p2p-Gnutella24	26518	65369	18965	18965	0.00%
.	p2p-Gnutella25	22687	54705	16478	16478	0.00%
	p2p-Gnutella30	36682	88328	26965	26966	0.00%
	p2p-Gnutella31	62586	147892	46227	46227	0.00%
Social Influence [40–42]	soc-sign-epinions	131828	841372	78952	79155	0.15%
	Wiki-Talk	2394385	5021410	2317964	2318078	0.004%
	Wiki-Vote	7115	103689	4736	4736	0.00%
	physician-discuss-rev	231	498	85	85	0.00%
Neural [43–46]	celegans	297	2345	49	49	0.00%
	cocomac	5336	36758	3577	3577	0.00%
	mac95	94	2390	9	9	0.00%
Social [30, 47]	email-EuAll	265214	420045	245791	245791	0.00%
	cons-frequency-rev	46	879	2	2	0.00%
	manuf-famiarity-rev	77	2326	0	0	0.00%
	manuf-frequency-rev	77	2228	1	1	0.00%
	physician-friend-rev*	228	506	52	52	0.00%
	Slashdot0811	77360	905468	49	49	0.00%
	Slashdot0902	82168	948464	3737	3737	0.00%
	soc-pokec-relationships	1023163	8239685	838575	838576	0.00%
Amazon [48]	amazon0302**	262111	1234877	8458	13326	1.86%
	amazon0505**	410236	3356824	14839	22592	1.88%
UC Irvine Messaging [49]	one-mode-char	1899	20296	614	625	0.58%
Corporate Ownership [50]	corp-own	7253	6713	5777	5777	0.00%
Transcription Yeast [51]	yeast	688	1079	565	565	0.00%

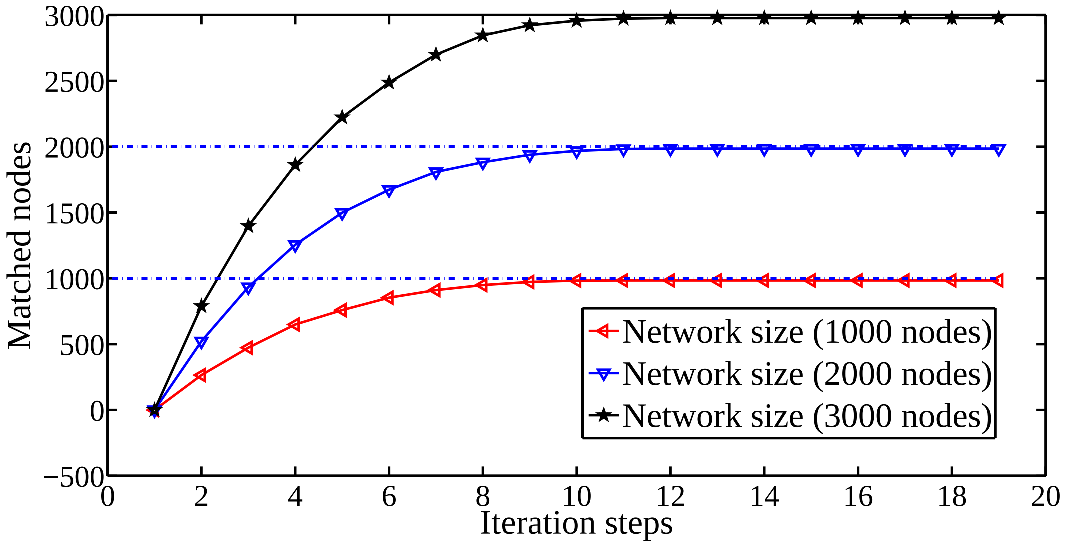

Figure S11: **Number of iterations vs. number of matched nodes** in ER networks with different network sizes but the same average nodal degree. Note that the number of driver nodes (unmatched nodes) equals N minus the final number of matched nodes.

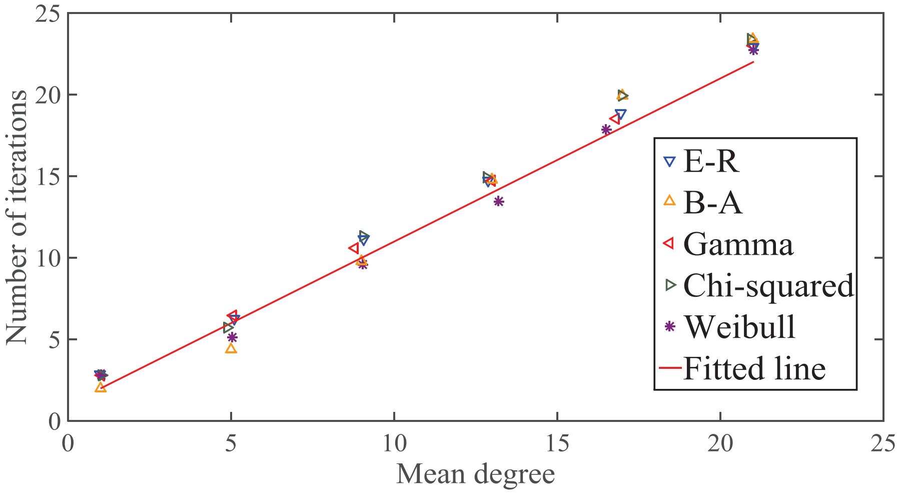

Figure S12: **Number of iterations in synthetic networks at** $w = 0$**.** The fitted line is $y = x + 1$.

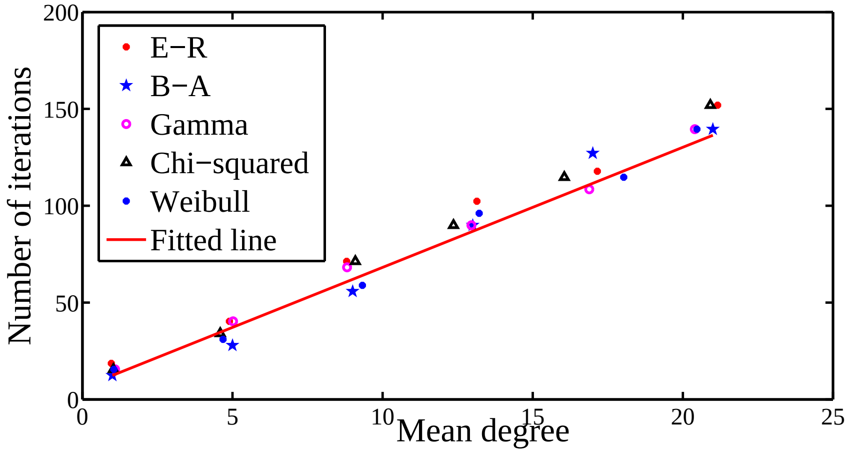

Figure S13: **Number of iterations in synthetic networks at $w = \frac{2}{3}$.** The fitted line is $y = 6.11x + 1.12$.

in which the convergence speed of the algorithm is determined by how quickly a match of requests can be achieved. This can be affected by the number of parent/child nodes possessed by each node, and is therefore related to the average nodal degree but is essentially unaffected by network size. This conclusion holds in all the synthetic networks we have tested.

The LM method requests simple local operations (i.e., child/parent locating and matching operations) in each iteration. We find that the number of iterations needed is consistently low. In all the networks that we have tested, the number of iterations $\mathbf{k}$ turns out to be approximately linearly proportional to the average node degree μ. Figures S12 and Figure S13 show that this conclusion holds in synthetic networks. Specifically, setting the waiting probability $w = 0$, we repeat the simulation in 10 independently generated synthetic networks with $N = 10000$ and plot the average results. The "fitted line" in each figure is generated by mean square error curve fitting. As shown in Figure S12, in all the synthetic networks, the numbers of iterations needed approximate a simple linear function of the average nodal degree $\hat{\mathbf{k}} = a\mu + b$, where both the slope a and the intercept b are approximately 1. When the waiting probability $w \neq 0$, e.g., when $w = \frac{2}{3}$, as shown in Figure S13, the linear relationship between the number of iterations needed and the mean nodal degree remains valid, although the values of a and b differ.

We also test the number of iterations $\mathbf{k}$ in 30 real-life networks listed in Table S3, where the mean nodal degree varies approximately from $\mu = 2$ to $\mu = 30$ with different values of the waiting probability ($w = 0$, $w = 0.1$, $w = 0.3$, $w = 0.5$, $w = \frac{2}{3}$, and $w = 0.8$). Figures S14 and S15 show that the linear relationship between $\mathbf{k}$ and μ basically remains valid in real-life networks. When w increases, the slope of the fitted line also increases. This is to be expected because a large value of w indicates a longer average waiting time before the decision to randomly break the tie is made. Note that the slope of the

fitted line for synthetic networks with a particular w is steadily close to the slope for real-life networks with the same w; the main difference is that the linear relationship becomes more significant in synthetic networks. This is because synthetic networks are random networks while real-life networks have distinct local structures which may significantly affect the number of iterations.

2.6 Effects of the waiting probability w.

Introducing a non-zero waiting probability w often improves the performance of the LM algorithm. Figures S16(a) and S16(b) show that this is the case because it decreases the probability that the better of two solutions is overlooked when a tie is randomly broken. The effects of the waiting probability w needs further study, however. Although a higher w value lowers the probability of overlooking the better of two solutions in random tie breaking, the cost is that a larger number of iterations is needed. As an example, we compare the algorithm performance under different values of $E(n_{\text{wait}})$ where, for a given value of w, $E(n_{\text{wait}}) = \frac{w}{1-w}$, as stated in Theorem 6, denotes the expected number of iterations a node needs to wait until making its decision.

Figures S16(c) and S16(d) show the matching performance, defined as $M_p(x) = 1 - (n_D^{LM}(x) - n_D^{MM})$, under different values of window size $E(n_{\text{wait}})$. Note that although a non-zero waiting probability w helps improve the network matching performance in the ER random networks and a number of real-life networks, moderate $E(n_{\text{wait}})$ values improve performance but very large $E(n_{\text{wait}})$ values (or w values approaching 1) do not significantly further improve performance. Setting $E(n_{\text{wait}}) = 4$ produces approximately the same results as setting $E(n_{\text{wait}}) \to \infty$. Even when $E(n_{\text{wait}}) = 2$ or $w = \frac{2}{3}$ the performance of LM approaches that of MM.

Since LM performs well in most real-life networks even when $w = 0$, this leaves little space for significant further improvement. Hence only five different synthetic and real-life networks are presented in Figures S16(c) and S16(d). When the performance of LM with $w = 0$ becomes less satisfactorily (e.g., in the five networks selected to be presented in the figure), the use of $w \neq 0$ improves performance. To keep a good balance between algorithm performance and the number of iterations, we set $E(n_{\text{wait}})$ in the range of $[2, 4]$.

Theorem 6. *In the waiting operation, if a node has a waiting probability w, the expected waiting time steps is $E(n_{wait}) = \frac{w}{1-w}$.*

Proof. Let $i = n_{wait}$. We know that i is a random variable which takes its value from 0 to $+\infty$.

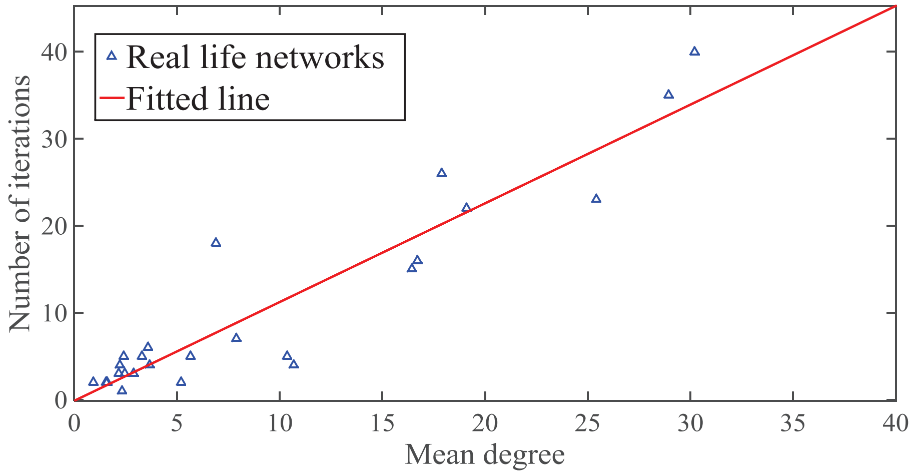

(a)

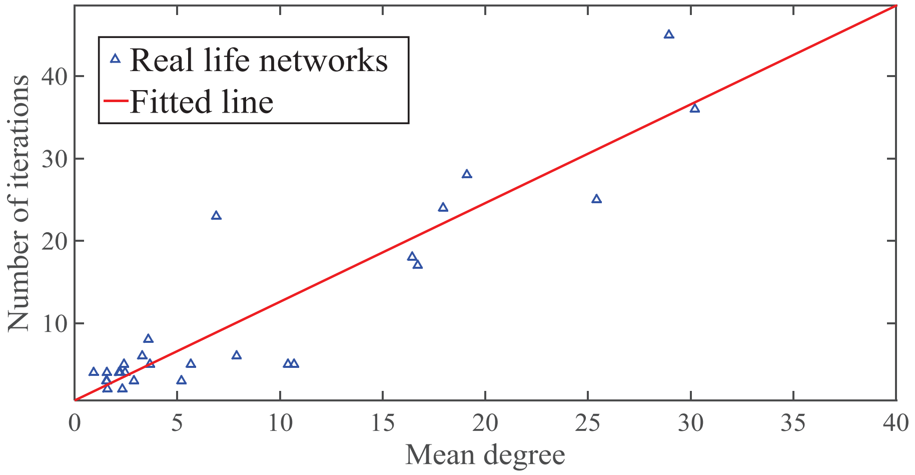

(b)

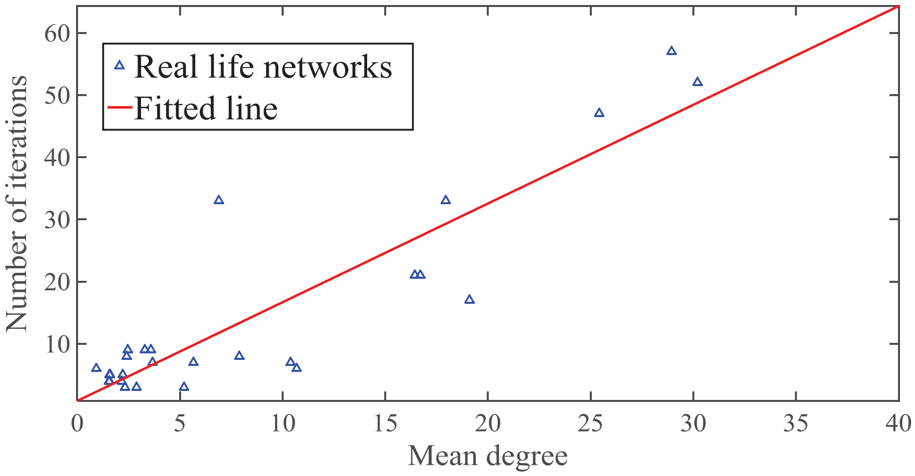

(c)

Figure S14: **LM in real-life networks: number of iterations vs. mean nodal degree.** The fitted lines are $y = 1.085x + 0.7$, $y = 1.2x + 0.62$ and $y = 1.59x + 0.81$ for $w = 0$, $w = 0.1$ and $w = 0.3$, respectively.

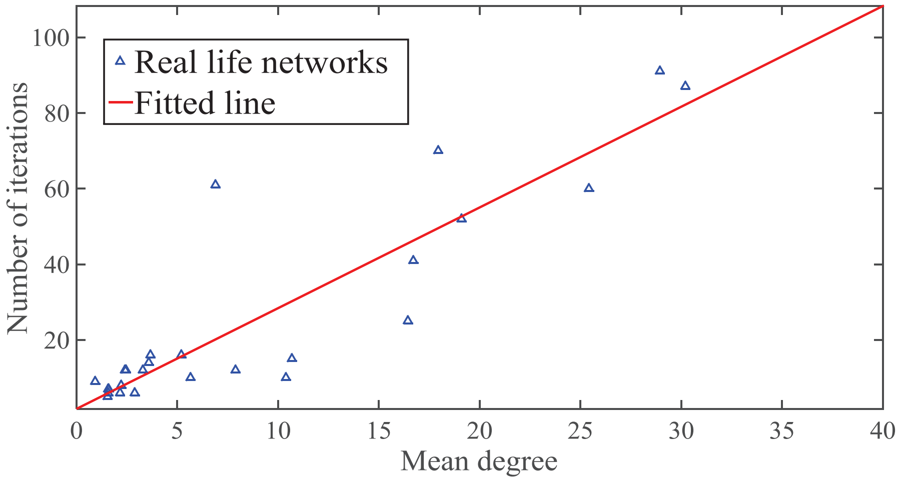

(a)

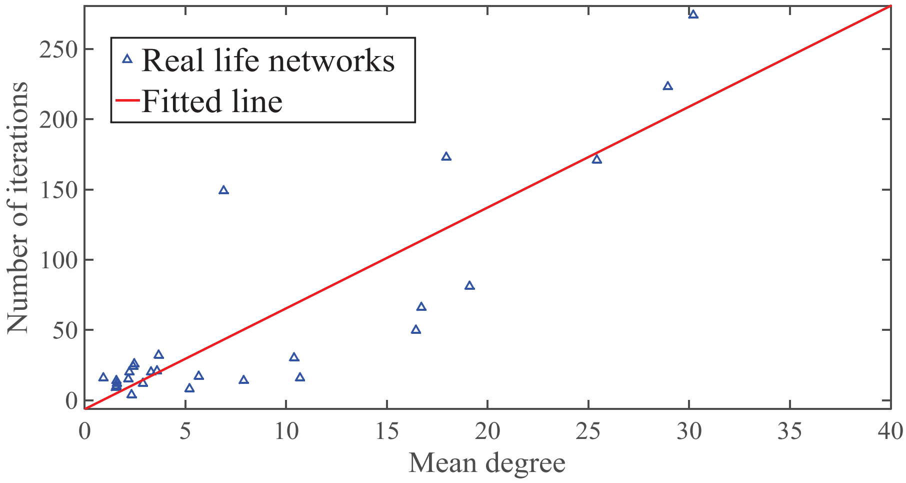

(b)

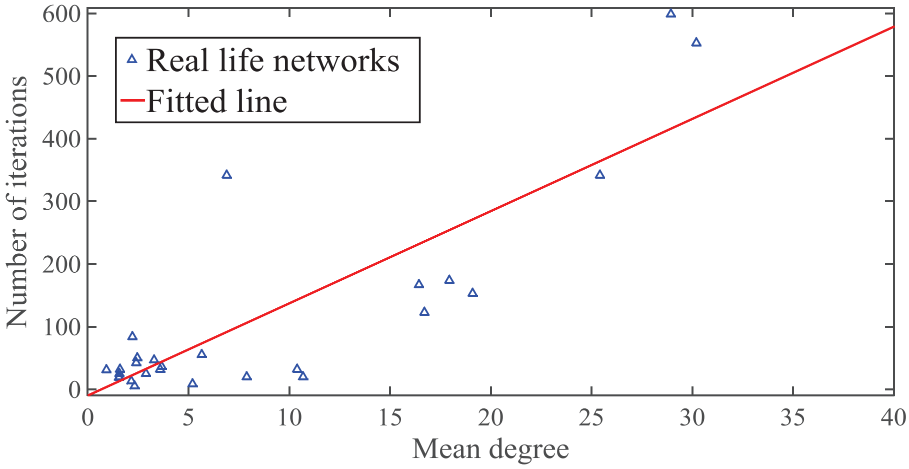

(c)

Figure S15: **LM in real-life networks: number of iterations vs. mean nodal degree.** The fitted lines are $y = 2.66x + 1.79$, $y = 7.18x - 6.33$ and $y = 14.71x - 10$ for $w = 0.5$, $w = \frac{2}{3}$, and $w = 0.8$, respectively.

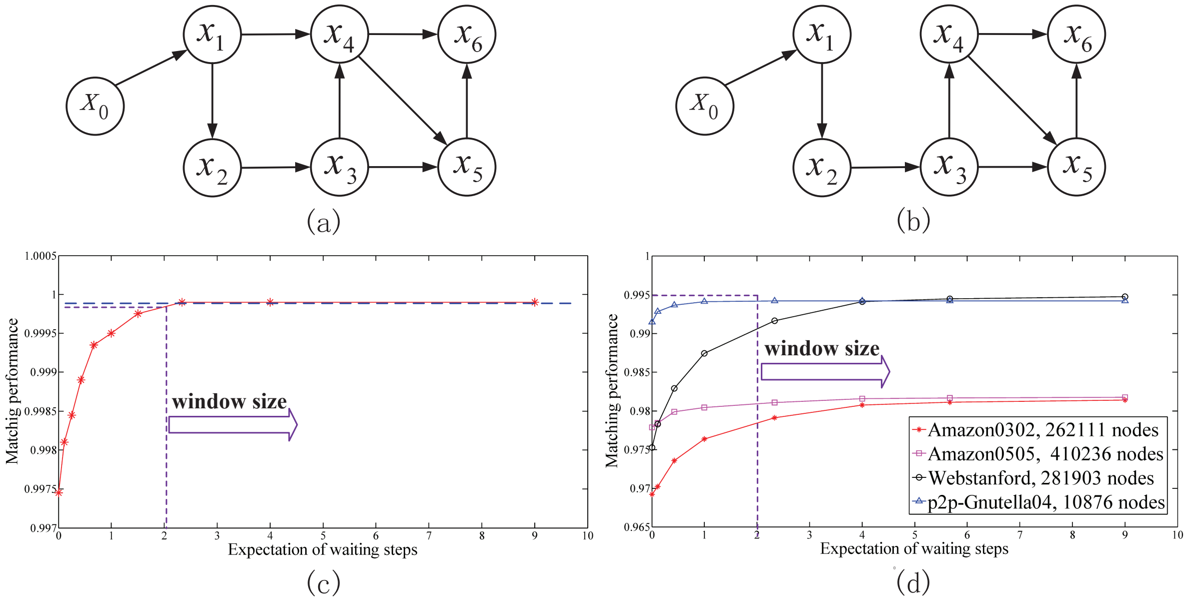

Figure S16: **Having a non-zero waiting probability w helps improve LM's performance.** **(a)** A simple example illustrating that having a non-zero waiting probability w may help improve the matching performance. As shown in (a), in the first iteration, x_3's child nodes (x_4 and x_5) have the same input degree, and x_4's parent nodes (x_1 and x_3) have the same output degree. If there is no waiting, x_3 and x_5 may achieve a match. If x_3 waits for one iteration, however, since x_1 and x_2 will achieve a match in the first iteration and the link between x_1 and x_4 will be removed as shown in **(b)** , x_3 shall match with x_4 in the second iteration. Finally, the optimal solution $x_0 \to x_1 \to x_2 \to ... \to x_6$ can be obtained. **(c)** Waiting helps improve LM's performance in an ER network ($N = 5000$, $\mu = 7$). **(d)** Waiting helps improve LM's performance in real-life networks.

Thus

$$E(n_{wait}) \quad = \sum_{i=1}^{+\infty} iw^i(1-w) = (1-w)\cdot(\sum_{i=0}^{+\infty}(i+1)w^i - \sum_{i=0}^{+\infty} w^i) \\ = (1-w)\cdot(S(w) - \frac{1}{1-w}) \tag{16}$$

where

$$S(w) = \sum_{i=0}^{+\infty}(i+1)w^i \tag{17}$$

Note that

$$\int S(w)dw = \int \sum_{i=0}^{+\infty}(i+1)w^i dw = \sum_{i=0}^{+\infty} w^{i+1} = \frac{w}{1-w} \tag{18}$$

then

$$S(w) = \frac{d\frac{w}{1-w}}{dw} = \frac{1}{(1-w)^2} \tag{19}$$

So finally we obtain that

$$E(n_{wait}) = (1-w)\cdot\frac{1}{(1-w)^2} - 1 = \frac{w}{1-w} \tag{20}$$

□

3 Implicit Linear Quadratic Regulator

3.1 Problem formulation of ILQR

As mentioned in the main paper, ILQR can be formulated as a matrix optimization problem under orthonormal boundary condition, where the objective is to drive the states from any initial state $x_0 \in R^{N\times 1}$ to converge to the origin ($\mathbf{x}(t_f) = \mathbf{0}$) during the time interval $[0, t_f]$ at the minimum cost, where the cost function is defined as $\mathbb{E}\{\int_0^{t_f} \mathbf{u}^T(t)\mathbf{u}(t)dt\}$ [52]; the operator $\mathbb{E}[\cdot]$ takes the expectation of the argument over all realizations of the random initial state. The problem of an ILQR is to determine an input matrix $B \in R^{N\times M}$ and also design control inputs $\mathbf{u} \in R^{M\times 1}$ such that the quadratic cost of controlling a directed network $\dot{\mathbf{x}}(t) = A\mathbf{x}(t) + B\mathbf{u}(t),\ \mathbf{x}(0) = \mathbf{x}_0$ is minimum. Since the pairs $B(t), \mathbf{u}(t)$ and $kB(t), \frac{1}{k}\mathbf{u}(t)$ represent the same inputs and outputs for a nonzero k, it is natural to fix the norm of matrix B. There are different ways of doing this and each way corresponds to a distinctive boundary

condition. In this paper, we consider the orthonormal boundary condition, i.e., B is restricted to be an orthonormal matrix. In this case, the minimum cost control problem is formulated as a constrained optimization problem, where the objective is to drive the system from an initial state x_0 to the origin during the time interval $[0, t_f]$ with the minimum cost:

$$\begin{aligned} &\min_{\mathbf{u},\,B} \quad \mathbb{E}\left[\int_0^{t_f} \|\mathbf{u}(t)\|^2\, dt\right] \\ s.t. \quad &B \in R^{N\times M} \; with \; B^T B = I_M \; and \; (A,B) \; is \; controllable \\ &\dot{\mathbf{x}}(t) = A\mathbf{x}(t) + B\mathbf{u}(t),\; \mathbf{x}(0) = \mathbf{x}_0, \mathbf{x}(t_f) = \mathbf{0} \end{aligned} \tag{21}$$

where $\mathbf{x}(t) = [x_1^T(t), ..., x_M^T(t)]^T$ and $\mathbf{u}(t) = [u_1^T(t), ..., u_M^T(t)]^T$ with M being the number of control inputs, and I_M is an identity matrix with dimension M. Note that both $\mathbf{u}$(t) and B are decision variables to be determined throughout the process in ILQR, where $\mathbf{u}$(t) specifies the values of controller inputs at each operating time and B determines the nodes to which the controller inputs are connected and the weights of the connections.

When B is selectable, both $\mathbf{x}(t)$ and $\mathbf{u}(t)$ become functions of B, which are denoted as $\mathbf{x}(t, B)$ and $\mathbf{u}(t, B)$, respectively. The energy cost function therefore becomes a function of t_f and B, which can be rewritten as

$$\begin{aligned} \min \quad & \mathbb{E}(t_f, B) = \mathbb{E}\left[\int_0^{t_f} \mathbf{u}^T(t,B)\mathbf{u}(t,B)dt\right] \\ s.t. \quad & B^T B = I_M, \\ & \dot{\mathbf{x}}(t) = A\mathbf{x}(t) + B\mathbf{u}(t),\; \mathbf{x}(0) = \mathbf{x}_0, \mathbf{x}(t_f) = \mathbf{0} \end{aligned} \tag{22}$$

where (A, B) is controllable (implies that $M > N_D$) and $\mathbf{x}(t, B) = [x_1^T(t, B), ..., x_M^T(t, B)]^T$ and $\mathbf{u}(t, B) = [u_1^T(t, B), ..., u_M^T(t, B)]^T$. In [53, 54], $\mathbf{u}(t, B)$ is given by

$$\mathbf{u}(t,B) \quad = -B^T e^{A^T(t_f - t)} W_B^{-1} e^{At_f}\mathbf{x}_0 \tag{23}$$

where W_B is the Gramian matrix

$$W_B \quad = \int_0^{t_f} e^{At} B B^T e^{A^T t} dt \tag{24}$$

This is because, with Equation (23), we have

$$\begin{aligned} \mathbf{x}(t_f, B) \quad &= e^{At}\mathbf{x}_0 + \int_0^{t_f} e^{A(t_f-\tau)} B\mathbf{u}(\tau)d\tau \\ &= e^{At_f}\mathbf{x}_0 - \int_0^{t_f} e^{A(t_f-\tau)} B B^T e^{A^T(t_f-\tau)} W_B^{-1} e^{At_f}\mathbf{x}_0 d\tau \\ &= e^{At_f}\mathbf{x}_0 - \left[\int_0^{t_f} e^{A(t_f-\tau)} B B^T e^{A^T(t_f-\tau)} d\tau\right] W_B^{-1} e^{At_f}\mathbf{x}_0 \end{aligned} \tag{25}$$

By letting $\tilde{t} = t_f - \tau$, we obtain

$$\begin{aligned}
\mathbf{x}(t_f, B) &= e^{At}\mathbf{x}_0 + \int_0^{t_f} e^{A(t_f-\tau)}B\mathbf{u}(\tau)d\tau \\
&= e^{At_f}\mathbf{x}_0 - \int_0^{t_f} e^{A\tilde{t}}BB^T e^{A^T\tilde{t}}W_B^{-1}e^{At_f}\mathbf{x}_0 d\tilde{t} \\
&= e^{At_f}\mathbf{x}_0 - \left[\int_0^{t_f} e^{A\tilde{t}}BB^T e^{A^T\tilde{t}}d\tilde{t}\right] W_B^{-1}e^{At_f}\mathbf{x}_0 \\
&= e^{At_f}\mathbf{x}_0 - W_B W_B^{-1}e^{At_f}\mathbf{x}_0 \\
&= 0
\end{aligned} \tag{26}$$

Thus for any given B satisfying that (A, B) is controllable, we can always design $\mathbf{u}(t)$ to drive the system from an arbitrary initial state to the origin in a fixed time interval $[0, t_f]$. For a given t_f, the control cost function $\mathbb{E}(.)$ is only dependent on the selection of B. Our objective is therefore to find an optimal input matrix B such that $\mathbb{E}(B)$ is minimized. Before presenting *orthonormal-condition-based projected gradient method* (OPGM) that searches such a B iteratively, we need to consider the derivative of $\mathbb{E}(t_f, B)$ with respect to B, i.e., $\frac{\partial \int_0^{t_f} \mathbf{u}^T(t,B)\mathbf{u}(t,B)dt}{\partial B}$ and $\frac{\partial \int_0^{t_f} \mathbf{x}^T(t,B)\mathbf{x}(t,B)dt}{\partial B}$, respectively.

From Equations (23) and (24), we obtain

$$\begin{aligned}
\int_0^{t_f} \mathbf{u}^T(t,B)\mathbf{u}(t,B)dt &= \int_0^{t_f} \mathbf{x}_0^T e^{A^T t_f}W_B^{-T}e^{A(t_f-t)}BB^T e^{A^T(t_f-t)}W_B^{-1}e^{At_f}\mathbf{x}_0 dt \\
&= \mathbf{x}_0^T e^{A^T t_f}W_B^{-T} \cdot \int_0^{t_f} e^{A(t_f-t)}BB^T e^{A^T(t_f-t)}dt \cdot W_B^{-1}e^{At_f}\mathbf{x}_0 \\
&= \mathbf{x}_0^T e^{A^T t_f}W_B^{-T} \cdot W_B \cdot W_B^{-1}e^{At_f}\mathbf{x}_0 \\
&= \mathbf{x}_0^T e^{A^T t_f}W_B^{-T}e^{At_f}\mathbf{x}_0 \\
&= \mathbf{x}_0^T e^{A^T t_f}W_B^{-1}e^{At_f}\mathbf{x}_0 \\
&= tr\left(\mathbf{x}_0^T e^{A^T t_f}W_B^{-1}e^{At_f}\mathbf{x}_0\right) \\
&= tr\left(W_B^{-1}e^{At_f}\mathbf{x}_0\mathbf{x}_0^T e^{A^T t_f}\right)
\end{aligned} \tag{27}$$

where $tr(.)$ denotes matrix trace operator.

By assuming that each element of initial state $\mathbf{x}_0 = [x_{01}, \ldots, x_{0N}]^T$ is an identical independent distributed (i.i.d) variable with zero mean and variance 1, we obtain a constrained non-convex matrix optimization problem with the input matrix B as its variables:

$$\begin{aligned}
&\min_B \quad \mathbb{E}(B) = tr\left(\left[\int_0^{t_f} e^{At}BB^T e^{A^T t}dt\right]^{-1} e^{At_f}\mathbb{E}(\mathbf{x}_0\mathbf{x}_0^T)e^{A^T t_f}\right) \\
&s.t. \quad (A, B) \ is\ controllable, \\
&\qquad\ B^T B = I_M
\end{aligned} \tag{28}$$

where $\mathbb{E}\left[\mathbf{x}_0\mathbf{x}_0^T\right] = I_N$. Note that (A, B) is controllable requires that $M \geq N_D$.

3.2 Convergence Proof of OPGM

To prove the convergence of OPGM proposed in Equations (2) and (3) in the main paper, we introduce the following lemmas first.

Lemma 2. *$B^TB = I_M$ is equivalent to $N(B) = 0$ where $N(B) = tr(B^TBB^TB) - 2tr(B^TB) + M$.*

Proof $\forall B \in \mathbb{R}^{N\times M}$, $B^TB = I_M \Leftrightarrow I_M - B^TB = 0 \Leftrightarrow tr\left((I_M - B^TB)^T(I_M - B^TB)\right) = 0 \Leftrightarrow tr(B^TBB^TB - 2B^TB + I_M) = 0 \Leftrightarrow N(B) = 0$. □

Lemma 3. *Given $x \geq 0$, for every $\varepsilon > 0$, there exists $\eta > 0$ such that*

$$|(1+\eta\cdot x)^{-\frac{1}{2}} - (1 - \tfrac{1}{2}\eta\cdot x)| < \varepsilon$$

Proof. The result can be easily obtained by applying Taylor expression. Thus the proof is omitted. □

Lemma 4. *Denote that $\hat{\mathcal{T}}_k = (I_N - B_kB_k^T)$ and $\frac{\partial\mathbb{E}(B_k)}{\partial B_k} = F(B_k)\cdot B_k$ where $F(B_k) \in \mathbb{R}^{N\times N}$ and*

$$F(B_k) = -\int_0^{t_f} 2e^{A^Tt}W_{B_k}^{-T}e^{At_f}\mathbb{E}(\mathbf{x}_0\mathbf{x}_0^T)e^{A^Tt_f}W_{B_k}^{-T}e^{At}dt$$

As $\mathbb{E}(\mathbf{x}_0\mathbf{x}_0^T) = I$, we have

$$F(B_k) = -\int_0^{t_f} 2e^{A^Tt}W_{B_k}^{-T}e^{At_f}e^{A^Tt_f}W_{B_k}^{-T}e^{At}dt$$

Also, by denoting that $\hat{B}_{k+1} = B_k - \eta\Delta B_k$ and $\Delta B_k = \hat{\mathcal{T}}_k\cdot\nabla\mathbb{E}(B_k) = \hat{\mathcal{T}}_k\cdot F(B_k)\cdot B_k$, we have

$$\hat{\mathcal{T}}_k^T = \hat{\mathcal{T}}_k,\ F^T(B_k) = F(B_k)$$

and

$$
\begin{aligned}
tr(\Delta {B_k}^T B_k B_k^T B_k) &= tr(B_k^T \Delta B_k B_k^T B_k) \\
&= tr(B_k^T B_k \Delta B_k^T B_k) \\
&= tr(B_k^T B_k B_k^T \Delta B_k), \\
tr(\Delta {B_k}^T \Delta B_k B_k^T B_k) &= tr(\Delta B_k^T B_k \Delta B_k^T B_k) \\
&= tr(B_k^T \Delta B_k \Delta B_k^T B_k) \\
&= tr(\Delta B_k^T B_k B_k^T \Delta B_k) \\
&= tr(B_k^T B_k \Delta B_k^T \Delta B_k) \\
&= tr(B_k^T \Delta B_k B_k^T \Delta B_k)
\end{aligned} \tag{29}
$$

and

$$
\begin{aligned}
tr(\Delta {B_k}^T \Delta B_k \Delta B_k^T B_k) &= tr(\Delta B_k^T \Delta B_k B_k^T \Delta B_k) \\
&= tr(\Delta B_k^T B_k \Delta B_k^T \Delta B_k) \\
&= tr(B_k^T \Delta B_k \Delta B_k^T \Delta B_k)
\end{aligned} \tag{30}
$$

Proof. It is easy to obtain that $F^T(B_k) = F(B_k)$ and $\hat{\mathcal{T}}_k^T = \hat{\mathcal{T}}_k$. From the definition of $F(B_k)$ and ΔB_k, we have

$$
\begin{aligned}
tr(\Delta B_k^T B_k B_k^T B_k) &= tr(B^T F(B_k)^T \hat{\mathcal{T}}_k^T B_k B_k^T B_k) \\
&= tr(B^T F(B_k) \hat{\mathcal{T}}_k B_k B_k^T B_k) \\
&= tr(B^T \hat{\mathcal{T}}_k F(B_k) B_k B_k^T B_k) \\
&= tr(B_k^T \Delta B_k B_k^T B_k).
\end{aligned} \tag{31}
$$

Similar to Equation (31), it is easy to obtain Equations (29) and (30). □

Lemma 5. *$\forall B \in \mathbb{R}^{N\times M}$ and $B \neq \mathbf{0}$, $\frac{tr^2(B^T B)}{tr(B^T B B^T B)} \leq M$.*

Proof. Let $Q = B^T B \in \mathbb{R}^{M\times M}$, and $\lambda_i (i = 1, 2, 3, ..., M)$ be the eigenvalue of Q. Then we have $Q^T = (B^T B)^T = B^T B = Q$, $tr(B^T B) = tr(Q)$, $tr(B^T B B^T B) = tr(Q^T Q) = tr(Q^2)$ and $\lambda_i^2 (i = 1, 2, 3, ..., M)$ is the eigenvalue of Q^2.

Considering $L=\sum_{i=1}^{M}(\lambda_i-\bar{\lambda})^2 \geq 0$, where $\bar{\lambda}=\frac{tr(Q)}{M}$, we obtain

$$\begin{aligned} L &= \textstyle\sum_{i=1}^{M}(\lambda_i-\bar{\lambda})^2 \\ &= \textstyle\sum_{i=1}^{k}\lambda_i^2 - 2\cdot\sum_{i=1}^{k}\lambda_i\cdot\bar{\lambda} + M\bar{\lambda}^2 \\ &= tr(Q^2) - 2\cdot tr(Q)\bar{\lambda} + M\bar{\lambda}^2 \\ &= tr(Q^2) - 2M\bar{\lambda}\cdot\bar{\lambda} + M\bar{\lambda}^2 \\ &= tr(Q^2) - M\bar{\lambda}^2 \\ &= tr(Q^2) - M\cdot\tfrac{tr^2(Q)}{M^2} \\ &\geq 0 \end{aligned} \tag{32}$$

Thus, we have $\frac{tr^2(Q)}{tr(Q^2)} \leq M \Rightarrow \frac{tr^2(B^TB)}{tr(B^TBB^TB)} \leq M.\square$

Lemma 6. *For a controllable (A,B), $F(B_k)$ is a negative definite matrix, where*

$$F(B_k) = -\int_0^{t_f} 2e^{A^Tt}W_{B_k}^{-T}e^{At}e^{A^Tt}TW_{B_k}^{-T}e^{At}dt \tag{33}$$

Proof. Let $C=\sqrt{2}\cdot e^{A^Tt}W_{B_k}^{-T}e^{At}$, then $F(B_k)$ can be written as $F(B_k)=-\int_0^{t_f}C^TCdt<0$, which means that $F(B_k)$ is a negative definite matrix. $\square$

Lemma 7. *For symmetric matrices $X,\ Y \in \mathbb{R}^{N\times N}$ and random matrices $U\in\mathbb{R}^{M\times N},\ Z\in\mathbb{R}^{N\times M}$, $tr(UXYZ)=tr(UYXZ)$, if ZU is symmetric.*

Proof. From $tr(Q^T)=tr(Q)$ and $tr(QGJ)=tr(GJQ)=tr(JQG), \forall Q,\ G,\ J$, we have

$$\begin{aligned} tr(UXYZ) &= tr(XYZU) \\ &= tr\left((XYZU)^T\right) \\ &= tr\left((ZU)^TY^TX^T\right) \\ &= tr(ZUYX) \\ &= tr(UYXZ). \end{aligned} \tag{34}$$

$\square$

Theorem 7. *For iterative process of OPGM:*

$$\begin{aligned} \hat{B}_{k+1} &= B_k - \eta\cdot\left(I_N - B_kB_k^T\right)\cdot\nabla\mathbb{E}(B_k) \\ B_{k+1} &= \sqrt{\frac{tr\left(\hat{B}_{k+1}^T\hat{B}_{k+1}\right)}{tr\left(\hat{B}_{k+1}^T\hat{B}_{k+1}\hat{B}_{k+1}^T\hat{B}_{k+1}\right)}}\cdot\hat{B}_{k+1} \end{aligned}$$

where $\nabla\mathbb{E}(B_k)$ is the gradient of $\nabla\mathbb{E}(B)$ at $B = B_k$, we have

$$\nabla\mathbb{E}(B) = -\frac{\partial E(B)}{\partial B} = -\int_0^{t_f} 2e^{A^Tt}W_B^{-T}e^{At_f}e^{A^Tt_f}W_B^{-T}e^{At}dt\cdot B$$

(which is given in Equations (2)-(3) in the main paper) is convergent, and $\mathbb{E}(B_k)$ converges to an extreme point $\mathbb{E}(B^*)$ where B^* has orthonormal columns, i.e., ${B^*}^TB^* = I_M$, if η is sufficiently small.

Proof. We use $\hat{\mathcal{T}}_k$, $\nabla N(B_k)$ and $\nabla\mathbb{E}(B)$ to denote $(I_N - B_kB_k^T)$, $\frac{\partial N(B_k)}{\partial B_K}$ and $\frac{\partial\mathbb{E}(B_k)}{\partial B_k}$ respectively. Our gradient descent method is to minimize $\mathbb{E}(B)$ and $N(B)$ simultaneously. The convergence is proven if we can establish

$$\mathbb{E}(B_{k+1}) - \mathbb{E}(B_k) \le 0,\ \textit{and}\ \mathbb{E}(B_k) \ge 0, \tag{35}$$

and

$$N(B_{k+1}) - N(B_k) \le 0,\ \textit{and}\ N(B_k) \ge 0 \tag{36}$$

From Lemma 5 and the iterative process of OPGM, we have

$$\begin{aligned}
&0 < tr(B_{k+1}^TB_{k+1})\\
&= tr\left(\left(\sqrt{\frac{tr\left(\hat{B}_{k+1}^T\hat{B}_{k+1}\right)}{tr\left(\hat{B}_{k+1}^T\hat{B}_{k+1}\hat{B}_{k+1}^T\hat{B}_{k+1}\right)}}\cdot\hat{B}_{k+1}\right)^T\left(\sqrt{\frac{tr\left(\hat{B}_{k+1}^T\hat{B}_{k+1}\right)}{tr\left(\hat{B}_{k+1}^T\hat{B}_{k+1}\hat{B}_{k+1}^T\hat{B}_{k+1}\right)}}\cdot\hat{B}_{k+1}\right)\right)\\
&= \frac{tr\left(\hat{B}_{k+1}^T\hat{B}_{k+1}\right)}{tr\left(\hat{B}_{k+1}^T\hat{B}_{k+1}\hat{B}_{k+1}^T\hat{B}_{k+1}\right)}\cdot tr(\hat{B}_{k+1}^T\hat{B}_{k+1})\\
&\le M,
\end{aligned} \tag{37}$$

and

$$\begin{aligned}
&tr(B_{k+1}^TB_{k+1}B_{k+1}^TB_{k+1})\\
=\ &\frac{tr^2\left(\hat{B}_{k+1}^T\hat{B}_{k+1}\right)}{tr^2\left(\hat{B}_{k+1}^T\hat{B}_{k+1}\hat{B}_{k+1}^T\hat{B}_{k+1}\right)}\cdot tr\left(\hat{B}_{k+1}^T\hat{B}_{k+1}\hat{B}_{k+1}^T\hat{B}_{k+1}\right)\\
=\ &\frac{tr^2\left(\hat{B}_{k+1}^T\hat{B}_{k+1}\right)}{tr\left(\hat{B}_{k+1}^T\hat{B}_{k+1}\hat{B}_{k+1}^T\hat{B}_{k+1}\right)}\\
=\ &tr(B_{k+1}^TB_{k+1})\\
>\ &0
\end{aligned} \tag{38}$$

For $N(B_{k+1})$, from Equations (37) and (38), we have

$$\begin{aligned}
N(B_{k+1})\ &= tr(B_{k+1}^TB_{k+1}B_{k+1}^TB_{k+1}) - 2tr(B_{k+1}^TB_{k+1}) + M\\
&= M - tr(B_{k+1}^TB_{k+1})\\
&\ge 0.
\end{aligned} \tag{39}$$

For $N(\hat{B}_{k+1}) - N(B_k)$, we have

$$\begin{aligned}
\nabla N(B_k) &= \tfrac{\partial N(B_k)}{\partial B_k} \\
&= \tfrac{\partial\left(tr(B_k^T B_k B_k^T B_k) - 2tr(B_k^T B_k) + M\right)}{\partial B_k} \\
&= 4B_k B_k^T B_k - 4B_k \\
&= 4(B_k B_k^T - I_N)B_k.
\end{aligned} \tag{40}$$

Using Taylor expansion to expand $N(B)$ [55], we have

$$\begin{aligned}
N(\hat{B}_{k+1}) &= N(B_k - \eta \Delta B_k) \\
&= N(B_k) - \eta tr(\nabla N(B_k)^T \cdot \Delta B_k) \\
&= N(B_k) - \eta tr\left(\left(4(B_k B_k^T - I_N)B_k\right)^T \hat{\mathcal{T}}_k F(B_k) B_k\right) \\
&= N(B_k) - 4\eta \cdot tr\left(B_k^T (B_k B_k^T - I_N)(I_N - B_k B_k^T) F(B_k) B_k\right) \\
&= N(B_k) + 4\eta \cdot tr\left(B_k^T (I_N - B_k B_k^T)(I_N - B_k B_k^T) F(B_k) B_k\right).
\end{aligned} \tag{41}$$

From Lemma 6 and Lemma 7, Equation (41) can be written as

$$\begin{aligned}
N(\hat{B}_{k+1}) - N(B_k) &= 4\eta \cdot tr\left(B_k^T (I_N - B_k B_k^T)(I_N - B_k B_k^T) F(B_k) B_k\right) \\
&= 4\eta \cdot tr\left(B_k^T (I_N - B_k B_k^T) F(B_k)(I_N - B_k B_k^T) B_k\right) \\
&\leq 0.
\end{aligned} \tag{42}$$

as $B_k B_k^T$ is symmetric and $F(B_k)$ is symmetric and negative definite.

Regarding $N(B_{k+1}) - N(\hat{B}_{k+1})$, we have

$$\begin{aligned}
&N(B_{k+1}) \\
&= N\left(\sqrt{\tfrac{tr\left(\hat{B}_{k+1}^T \hat{B}_{k+1}\right)}{tr\left(\hat{B}_{k+1}^T \hat{B}_{k+1} \hat{B}_{k+1}^T \hat{B}_{k+1}\right)}} \cdot \hat{B}_{k+1}\right) \\
&= \tfrac{tr^2\left(\hat{B}_{k+1}^T \hat{B}_{k+1}\right)}{tr^2\left(\hat{B}_{k+1}^T \hat{B}_{k+1} \hat{B}_{k+1}^T \hat{B}_{k+1}\right)} tr(\hat{B}_{k+1}^T \hat{B}_{k+1} \hat{B}_{k+1}^T \hat{B}_{k+1}) - 2\tfrac{tr\left(\hat{B}_{k+1}^T \hat{B}_{k+1}\right)}{tr\left(\hat{B}_{k+1}^T \hat{B}_{k+1} \hat{B}_{k+1}^T \hat{B}_{k+1}\right)} tr(\hat{B}_{k+1}^T \hat{B}_{k+1}) + M \\
&= M - \tfrac{tr^2\left(\hat{B}_{k+1}^T \hat{B}_{k+1}\right)}{tr\left(\hat{B}_{k+1}^T \hat{B}_{k+1} \hat{B}_{k+1}^T \hat{B}_{k+1}\right)}.
\end{aligned} \tag{43}$$

Thus,

$$\begin{aligned}
&N(B_{k+1}) - N(\hat{B}_{k+1}) \\
&= M - \tfrac{tr^2\left(\hat{B}_{k+1}^T \hat{B}_{k+1}\right)}{tr\left(\hat{B}_{k+1}^T \hat{B}_{k+1} \hat{B}_{k+1}^T \hat{B}_{k+1}\right)} - \left(tr(\hat{B}_{k+1}^T \hat{B}_{k+1} \hat{B}_{k+1}^T \hat{B}_{k+1}) - 2tr(\hat{B}_{k+1}^T \hat{B}_{k+1}) + M\right) \\
&= 2tr(\hat{B}_{k+1}^T \hat{B}_{k+1}) - \tfrac{tr^2\left(\hat{B}_{k+1}^T \hat{B}_{k+1}\right)}{tr\left(\hat{B}_{k+1}^T \hat{B}_{k+1} \hat{B}_{k+1}^T \hat{B}_{k+1}\right)} - tr(\hat{B}_{k+1}^T \hat{B}_{k+1} \hat{B}_{k+1}^T \hat{B}_{k+1}) \\
&= -\tfrac{\left(tr(\hat{B}_{k+1}^T \hat{B}_{k+1} \hat{B}_{k+1}^T \hat{B}_{k+1}) - tr(\hat{B}_{k+1}^T \hat{B}_{k+1})\right)^2}{tr(\hat{B}_{k+1}^T \hat{B}_{k+1} \hat{B}_{k+1}^T \hat{B}_{k+1})} \\
&\leq 0.
\end{aligned} \tag{44}$$

From Equations (42) and (44), we have

$$N(B_{k+1}) - N(B_k) = N(B_{k+1}) - N(\hat{B}_{k+1}) + N(\hat{B}_{k+1}) - N(B_k) \leq 0. \tag{45}$$

Thus the first inequality in (36) is proven. Now letting $Q = B^T B$ for Lemma 2, $N(B)$ becomes a convex function of Q. Note that since $N(Q)$ has a quadratic form, it has a unique global optimal point corresponding to $Q = B^T B = I$. Then, from Equations (39) and (45), the convergence of $N(B)$ is proven. This means that for any $\epsilon > 0$, $\exists K \in \mathbb{N}$ such that $\forall k > K$, $|N(B_k)| < \epsilon$, and this can be also written as

$$N(B_k) = o(\eta) \quad or \quad B_k^T B_k = I_M + o(\eta) \tag{46}$$

For $\mathbb{E}(B)$, we have

$$\begin{aligned} \mathbb{E}(B_k) &= \mathbb{E}\{\textstyle\int_0^{t_f} \mathbf{u}^T(t, B_k)\mathbf{u}(t, B_k)dt\} \\ &= \mathbb{E}\{\textstyle\int_0^{t_f} ||\mathbf{u}(t, B_k)||_2 dt\} \\ &\geq 0. \end{aligned} \tag{47}$$

To prove that $\mathbb{E}(B_{k+1}) - \mathbb{E}(B_k) \leq 0$, we first use B_k to express B_{k+1} directly. By letting $\Delta B_k = \hat{\mathcal{T}}_k \cdot \nabla \mathbb{E}(B_k)$, we have

$$\begin{aligned} & B_{k+1} \\ &= \sqrt{\frac{tr\left(\hat{B}_{k+1}^T \hat{B}_{k+1}\right)}{tr\left(\hat{B}_{k+1}^T \hat{B}_{k+1} \hat{B}_{k+1}^T \hat{B}_{k+1}\right)}} \cdot \hat{B}_{k+1} \\ &= \sqrt{\frac{tr\left(\hat{B}_{k+1}^T \hat{B}_{k+1}\right)}{tr\left(\hat{B}_{k+1}^T \hat{B}_{k+1} \hat{B}_{k+1}^T \hat{B}_{k+1}\right)}} \cdot (B_k - \eta \cdot \Delta B_k) \\ &= \sqrt{\frac{tr((B_k - \eta\Delta B_k)^T (B_k - \eta\Delta B_k))}{tr((B_k - \eta\Delta B_k)^T (B_k - \eta\Delta B_k)(B_k - \eta\Delta B_k)^T (B_k - \eta\Delta B_k))}} \cdot (B_k - \eta \cdot \Delta B_k) \\ &= \left(tr(B_k^T B_k - \eta \Delta B_k^T B_k - \eta B_k^T \Delta B_k + \eta^2 \Delta B_k^T \Delta B_k)\right)^{\frac{1}{2}} \cdot \Big(tr(B_k^T B_k B_k^T B_k - \Delta B_k^T B_k B_k^T B_k - B_k^T \Delta B_k B_k^T B_k \\ &\quad + \Delta B_k^T \Delta B_k B_k^T B_k - B_k^T B_k \Delta B_k^T B_k + \Delta B_k^T B_k \Delta B_k^T B_k + B_k^T \Delta B_k \Delta B_k^T B_k \\ &\quad - \Delta B_k^T \Delta B_k \Delta B_k^T B_k - B_k^T B_k B_k^T \Delta B_k \\ &\quad + \Delta B_k^T B_k B_k^T \Delta B_k + B_k^T \Delta B_k B_k^T \Delta B_k - \Delta B_k^T \Delta B_k B_k^T \Delta B_k + B_k^T B_k \Delta B_k^T \Delta B_k \\ &\quad - \Delta B_k^T B_k \Delta B_k^T \Delta B_k - B_k^T \Delta B_k \Delta B_k^T \Delta B_k + \Delta B_k^T \Delta B_k \Delta B_k^T \Delta B_k)\Big)^{-\frac{1}{2}} \cdot (B_k - \eta \cdot \Delta B_k) \end{aligned} \tag{48}$$

From Lemma 2 and Lemma 3, Equation (48) can be written as

$$
\begin{aligned}
&B_{k+1} \\
&= \left(tr(B_k^T B_k) - 2\eta tr(\Delta B_k^T B_k) + \eta^2 tr(\Delta B_k^T \Delta B_k)\right)^{\frac{1}{2}} \cdot (tr(B_k^T B_k B_k^T B_k) - 4\eta tr(\Delta B_k^T B_k B_k^T B_k) \\
&\quad + 6\eta^2 tr(\Delta B_k^T \Delta B_k B_k^T B_k) - 4\eta^3 tr(\Delta B_k^T \Delta B_k \Delta B_k^T B_k) + \eta^4 tr(\Delta B_k^T \Delta B_k \Delta B_k^T \Delta B_k))^{-\frac{1}{2}} \cdot (B_k - \eta \cdot \Delta B_k) \\
&= \left(tr(B_k^T B_k)\left(1 - 2\eta \frac{tr(\Delta B_k^T B_k)}{tr(B_k^T B_k)} + o(\eta)\right)\right)^{\frac{1}{2}} \cdot \left(tr(B_k^T B_k B_k^T B_k)\left(1 - 4\eta \frac{tr(\Delta B_k^T B_k B_k^T B_k)}{tr(B_k^T B_k B_k^T B_k)} + o(\eta)\right)\right)^{-\frac{1}{2}} \cdot (B_k - \eta \cdot \Delta B_k) \\
&= tr^{\frac{1}{2}}(B_k^T B_k)\left(1 - \eta \frac{tr(\Delta B_k^T B_k)}{tr(B_k^T B_k)} + o(\eta)\right) \cdot tr^{-\frac{1}{2}}(B_k^T B_k B_k^T B_k)\left(1 + 2\eta \frac{tr(\Delta B_k^T B_k B_k^T B_k)}{tr(B_k^T B_k B_k^T B_k)} + o(\eta)\right) \cdot (B_k - \eta \cdot \Delta B_k) \\
&= \sqrt{\frac{tr(B_k^T B_k)}{tr(B_k^T B_k B_k^T B_k)}}\left(1 - \eta \frac{tr(\Delta B_k^T B_k)}{tr(B_k^T B_k)} + 2\eta \frac{tr(\Delta B_k^T B_k B_k^T B_k)}{tr(B_k^T B_k B_k^T B_k)} + o(\eta)\right) \cdot (B_k - \eta \cdot \Delta B_k)
\end{aligned}
\tag{49}
$$

By using Equation (38) we have $\sqrt{\frac{tr(B_k^T B_k)}{tr(B_k^T B_k B_k^T B_k)}} = 1$. Then applying Taylor expansion to expand $\mathbb{E}(B)$ [55], we have

$$
\begin{aligned}
&\mathbb{E}(B_{k+2}) \\
&= \mathbb{E}\left(\left(1 - \eta \frac{tr(\Delta B_{k+1}^T B_{k+1})}{tr(B_{k+1}^T B_{k+1})} + 2\eta \frac{tr(\Delta B_{k+1}^T B_{k+1} B_{k+1}^T B_{k+1})}{tr(B_{k+1}^T B_{k+1} B_{k+1}^T B_{k+1})} + o(\eta)\right) \cdot (B_{k+1} - \eta \cdot \Delta B_{k+1})\right) \\
&= \mathbb{E}\left(B_{k+1} - \left(\eta \frac{tr(\Delta B_{k+1}^T B_{k+1})}{tr(B_{k+1}^T B_{k+1})} - 2\eta \frac{tr(\Delta B_{k+1}^T B_{k+1} B_{k+1}^T B_{k+1})}{tr(B_{k+1}^T B_{k+1} B_{k+1}^T B_{k+1})}\right) \cdot B_{k+1} - \eta \Delta B_{k+1} + o(\eta)\right) \\
&= \mathbb{E}(B_{k+1}) - \eta tr\left(\nabla \mathbb{E}^T(B_{k+1})\left[\left(\frac{tr(\Delta B_{k+1}^T B_{k+1})}{tr(B_{k+1}^T B_{k+1})} - 2\frac{tr(\Delta B_{k+1}^T B_{k+1} B_{k+1}^T B_{k+1})}{tr(B_{k+1}^T B_{k+1} B_{k+1}^T B_{k+1})}\right) B_{k+1} + \Delta B_{k+1}\right]\right) + o(\eta) \\
&= \mathbb{E}(B_{k+1}) - \eta\left(\frac{tr(\Delta B_{k+1}^T B_{k+1})}{tr(B_{k+1}^T B_{k+1})} - 2\frac{tr(\Delta B_{k+1}^T B_{k+1} B_{k+1}^T B_{k+1})}{tr(B_{k+1}^T B_{k+1} B_{k+1}^T B_{k+1})}\right) \cdot tr(\nabla \mathbb{E}^T(B_{k+1}) B_{k+1}) - \eta tr(\nabla \mathbb{E}^T(B_{k+1}) \Delta B_{k+1}) + o(\eta) \\
&= \mathbb{E}(B_{k+1}) - \eta\left(\frac{tr(\Delta B_{k+1}^T B_{k+1})}{tr(B_{k+1}^T B_{k+1})} - 2\frac{tr(\Delta B_{k+1}^T B_{k+1} B_{k+1}^T B_{k+1})}{tr(B_{k+1}^T B_{k+1} B_{k+1}^T B_{k+1})}\right) \cdot tr(B_{k+1}^T F^T(B_{k+1}) B_{k+1}) \\
&\quad - \eta tr(B_{k+1}^T F^T(B_{k+1}) \tilde{\mathcal{T}}_{k+1} F(B_{k+1}) B_{k+1}) + o(\eta)
\end{aligned}
\tag{50}
$$

From Equation (46), $\exists K \in \mathbb{N},\ \forall k > K$, we have $B_k^T B_k = I_M + o(\eta)$. Then, it follows that

$$
\begin{aligned}
\hat{\mathcal{T}}_{k+1}^2 &= (I_N - B_{k+1} B_{k+1}^T)^2 \\
&= I_N - B_{k+1} B_{k+1}^T - B_{k+1} B_{k+1}^T + B_{k+1} B_{k+1}^T B_{k+1} B_{k+1}^T \\
&= I_N - B_{k+1} B_{k+1}^T - B_{k+1} B_{k+1}^T + B_{k+1}\left(I_M + o(\eta)\right) B_{k+1}^T \\
&= I_N - B_{k+1} B_{k+1}^T + o(\eta) \\
&= \hat{\mathcal{T}}_{k+1} + o(\eta).
\end{aligned}
\tag{51}
$$

Thus,

$$
\begin{aligned}
\eta tr(B_{k+1}^T F^T(B_{k+1}) \hat{\mathcal{T}}_{k+1} F(B_{k+1}) B_{k+1}) &= \eta tr\left(B_{k+1}^T F^T(B_{k+1})\left(\hat{\mathcal{T}}_{k+1}^2 + o(\eta)\right) F(B_{k+1}) B_{k+1}\right) \\
&= \eta tr\left(B_{k+1}^T F^T(B_{k+1}) \hat{\mathcal{T}}_{k+1}^2 F(B_{k+1}) B_{k+1}\right) + o(\eta).
\end{aligned}
\tag{52}
$$

Likewise, for

$$\eta\left(\frac{tr(\Delta B_{k+1}^T B_{k+1})}{tr(B_{k+1}^T B_{k+1})} - 2\frac{tr(\Delta B_{k+1}^T B_{k+1} B_{k+1}^T B_{k+1})}{tr(B_{k+1}^T B_{k+1} B_{k+1}^T B_{k+1})}\right) \cdot tr(B_{k+1}^T F^T(B_{k+1})B_{k+1})$$

we have

$$\begin{aligned} tr(\Delta B_{k+1}^T B_{k+1}) &= tr\left(B_{k+1}^T F^T(B_{k+1})(I_N - B_{k+1}B_{k+1}^T)B_{k+1}\right) \\ &= tr\left(B_{k+1}^T F^T(B_{k+1})(B_{k+1} - B_{k+1}B_{k+1}^T B_{k+1})\right) \\ &= tr\left(B_{k+1}^T F^T(B_{k+1})\left(B_{k+1} - B_{k+1}(I_M + o(\eta))\right)\right) \\ &= tr\left(B_{k+1}^T F^T(B_{k+1})\left(B_{k+1} - B_{k+1} + o(\eta)\right)\right) \\ &= o(\eta), \end{aligned} \tag{53}$$

and

$$\begin{aligned} tr(\Delta B_{k+1}^T B_{k+1} B_{k+1}^T B_{k+1}) &= tr\left(B_{k+1}^T F^T(B_{k+1})(I_N - B_{k+1}B_{k+1}^T) \cdot B_{k+1}B_{k+1}^T B_{k+1}\right) \\ &= tr\left(B_{k+1}^T F^T(B_{k+1})(B_{k+1} - B_{k+1}B_{k+1}^T B_{k+1}) \cdot B_{k+1}^T B_{k+1}\right) \\ &= tr\left(B_{k+1}^T F^T(B_{k+1})\left(B_{k+1} - B_{k+1}(I_M + o(\eta))\right) \cdot B_{k+1}^T B_{k+1}\right) \\ &= tr\left(B_{k+1}^T F^T(B_{k+1})\left(B_{k+1} - B_{k+1} + o(\eta)\right) \cdot B_{k+1}^T B_{k+1}\right) \\ &= o(\eta). \end{aligned} \tag{54}$$

Thus, we have

$$\eta\left(\frac{tr(\Delta B_{k+1}^T B_{k+1})}{tr(B_{k+1}^T B_{k+1})} - 2\frac{tr(\Delta B_{k+1}^T B_{k+1} B_{k+1}^T B_{k+1})}{tr(B_{k+1}^T B_{k+1} B_{k+1}^T B_{k+1})}\right) \cdot tr(B_{k+1}^T F^T(B_{k+1})B_{k+1}) = o(\eta). \tag{55}$$

With Equations (52) and (55), Equation (50) can be expressed as $\exists K \in \mathbb{N},\ \forall k > K$,

$$\mathbb{E}(B_{k+2}) - \mathbb{E}(B_{k+1}) = -\eta tr\left(B_{k+1}^T F^T(B_{k+1})\hat{\mathcal{T}}_{k+1}^2 F(B_{k+1})B_{k+1}\right) + o(\eta) \leq 0. \tag{56}$$

From Equations (47) and (56), the convergence of $\mathbb{E}(B_k)$ is shown for a sufficiently small η. By combining the results shown in Equation (46), $\mathbb{E}(B_k)$ converges to $\mathbb{E}(B^*)$ with B^* being an orthonormal matrix. □

Remarks: The update rule from $\hat{B}_{k+1}$ to B_{k+1} in Equation (3) of the main paper is explained as follows. We define a norm function as

$$N(B) = tr\left(\left(B^T B - I_M\right)^T \left(B^T B - I_M\right)\right) = tr(B^T B B^T B) - 2tr(B^T B) + M \tag{57}$$

Note that $\forall B \in \mathbb{R}^{N\times M}$, $B^T B = I_M$ is equivalent to $N(B) = 0$. Let $B_{k+1} = \rho_k \cdot \hat{B}_{k+1}$. It is seen that there is no exact solution ρ_k satisfying that $N(B_{k+1}) = 0$ since it is generally impossible to drag a tensor

of B on the boundary constraints by just automatic scaling. In the iteration, we minimize $N(B_{k+1})$ by solving $\frac{\partial N(\rho_k \cdot \hat{B}_{k+1})}{\partial \rho_k} = 0$, which gives

$$N(\rho_k) = \rho_k^4 tr(\hat{B}_{k+1}^T \hat{B}_{k+1} \hat{B}_{k+1}^T \hat{B}_{k+1}) - 2\rho_k^2 tr(\hat{B}_{k+1}^T \hat{B}_{k+1}) + M \tag{58}$$

Then we can obtain $\rho_k = \sqrt{\frac{tr\left(\hat{B}_{k+1}^T \hat{B}_{k+1}\right)}{tr\left(\hat{B}_{k+1}^T \hat{B}_{k+1} \hat{B}_{k+1}^T \hat{B}_{k+1}\right)}}$. □

3.3 Control node selection

Note that both B_0 and B^* obtained by OPGM imply that each node can be connected to each of the M controllers, which may not yield a practical solution for large-size networks. A practical and interesting problem therefore is to find the minimum cost control solution under the constraint that only a small set of nodes can be directly connected to external controllers. We term this small node set as *control node set* and consider the case where each control node can be connected to only a unique controller for achieving the lowest connection complexity.

For this case, we propose a very simple approach to derive the control node set from B^*. Note that the absolute value of link weight $\left|B_{ij}^*\right|$ reflects the importance of the node i for the $j-$th controller. Define an *importance index* vector

$$r = \frac{[r_1 \ \dots \ r_i \ \dots \ r_N]}{\max(r_1, \dots, r_i, \dots, r_N)} \tag{59}$$

where $r_i = \sum_j \left|B_{ij}^*\right|$ for $i = 1, ..., N$. Clearly, we have $\max\{r\} = 1$. The importance index of node i evaluates the relative importance of this node in achieving the minimum cost control objective. We form the *control node set* as the first M nodes with the largest importance index values. The corresponding connection matrix, denoted as $\mathcal{B}^*$, can be easily constructed: if a node i is the $k-$th node of the control node set, we set $\mathcal{B}_{i,k}^* = 1$; otherwise, $\mathcal{B}_{i,k}^* = 0$.

3.4 Comparisons between PGM and OPGM

Note that the matrix optimization model in the main paper with orthornormal boundary constraint $B^T B = I_M$ is revisited from the trace boundary constraint $tr(B^T B) = M$ in one of our most recent work in [52], where *projected gradient method* (PGM) is proposed to locate the control nodes and then determine input matrix B.

Our finding is that the orthornormal boundary condition slightly outperforms trace boundary constraint for the problem of optimal cost control of complex networks since the control nodes selected by OPGM is more concentrated on the nodes that divide an elementary dilation equally for achieving a lower energy cost. The experiment is done in a dilation topology with $N = 6, M = 3$. To ensure the

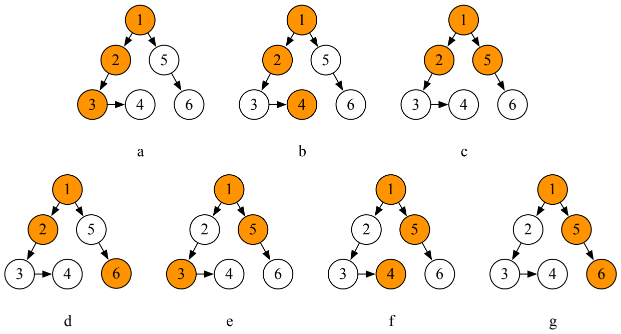

Figure S17: **Control nodes by OPGM and PGM on an elementary dilation.** The length of the dilation is $N = 6$ and the number of control node is $M = 3$.

controllability of the networks in Figure S17, node 1 must be selected as a control node. When node 1 is fixed as a control node, there are totally 7 types of control nodes selection method which ensure the controllability of the network. Among them, control node set $\{node\ 1, node\ 3, node\ 5\}$ divides the dilation equally, and it consumes the lowest energy. By experimenting the projected gradient algorithms under different boundary constraints repeatedly, we could obtain the estimated probability for each control node set selection method. The experimental results are presented in Table S4.

We observe that OPGM tends to divide the dilation equally with a higher probability for consuming less energy. By repeating the simulation 10000 times, the probability for selecting $\{node\ 1, node\ 3, node\ 5\}$ is around 44.63% for OPGM, which is higher than that of PGM.

In addition, if we orthogonalize B_0 to satisfy that $B_0^T B_0 = I_M$, the performance of OPGM can be further improved as the selected control nodes are more concentrated on node set $\{node\ 1, node\ 3, node\ 5\}$. This implies that although convergence of OPGM is guaranteed under arbitrary initial B_0, having an orthogonalized initial B_0 helps improve the performance significantly.

Table S4: **Comparisons of OPGM and PGM on an elementary dilation.** Control nodes selected by OPGM are more concentrated on the nodes dividing the elementary dilation equally. The length of the dilation is $N = 6$ and the number of control node is $M = 3$. For conciseness, $\{node\ 1, node\ 3, node\ 5\}$ is written as $(1, 3, 5)$.

Algorithm	Initial condition	(1,3,5)	(1,2,5)	(1,2,4)	(1,4,5)	(1,2,3)	(1,5,6)	(1,2,6)
PGM	$tr(B_0^T B_0) = M$	39.95%	16.60%	12.06%	14.79%	11.54%	2.98%	2.08%
OPGM	Arbitrary	44.63%	14.70%	12.26%	14.02%	11.61%	1.74%	1.05%
OPGM	$B_0^T B_0 = I_M$	65.685%	34.229%	0.057%	0.029%	0.000%	0.000%	0.000%

4 Minimizing Longest Control Path

For the illustration of MLCP, first locate original N_D^{LM} directed control paths (DCPs) and N_C circled control paths (CCPs) using LM algorithm. Denote the length of each DCP and CCP as L_i for $1 \leq i \leq$

N_D^{LM}, and C_j for $1 \leq j \leq N_C$, respectively. In implementing MLCP, it is assumed that all nodes have the same information that LM obtained. The steps of MLCP are presented as follows.

4.1 MLCP Algorithm

The detailed flow of MLCP algorithm is as follows:

Step 1. Randomly select a CCP ($j-$th path, for example), and break it by removing one of its links; then imagine that it is connected to the end node of a randomly selected LCP ($i-$th path, for example) to form a new DCP. Whether a physical link exists between the end node of the broken CCP and the end of the randomly selected LCP does not matter, as we shall see more clearly later. Update the length of the path to $L_i + C_j \to L_i$; repeat this process until there is no CCP left. Finally we shall still have N_D^{LM} DCPs.

Step 2. Determine the assignment of the m_0 new external control inputs to these newly formed DCPs. Suppose that each path is assigned n_i control inputs, obviously we have $\sum_i n_i = m_0$:

Initialize $n_i = 0$ for all $1 \leq i \leq N_D^{LM}$

for $k = 1, 2, ..., m_0$

if $\frac{L_i}{1+n_i}$ attains the maximum at the i-th DCP.

then $n_i = n_i + 1$

end

end

Step 3. For each DCP, mark the $\kappa \cdot \ulcorner \frac{L_i}{1+\kappa} \urcorner + 1$ th node as an *independent control node* (refer to the concept definition section) for the $i-$th path where $\kappa = 0, 1, \ldots, n_i$. Totally there are $N_D^{LM} + m_0$ marked nodes and each should be connected to an external control input.

Step 4. If there is no *independent control node* in one of the original CCPs, randomly select one node and mark it as a *dependent control node*, which is connected a nearest existing external input.

As can be seen from the above discussions, only a small set of nodes are determined as control nodes and to be connected to external inputs, and each control node can only be binary connected to one of the M external input, which yields a practical solution for large-size networks. For more detailed information, please refer to the following example.

4.2 Example illustration

A simple example is presented in Figure S18 to illustrate the implementation of the MLCP algorithm. As shown in Figure S18(a), the network has 15 nodes and 18 edges. Now we have $m_0 = 2$ extra external control inputs. After LM algorithm, there are four segments left as shown in Figure S18(b), i.e. two

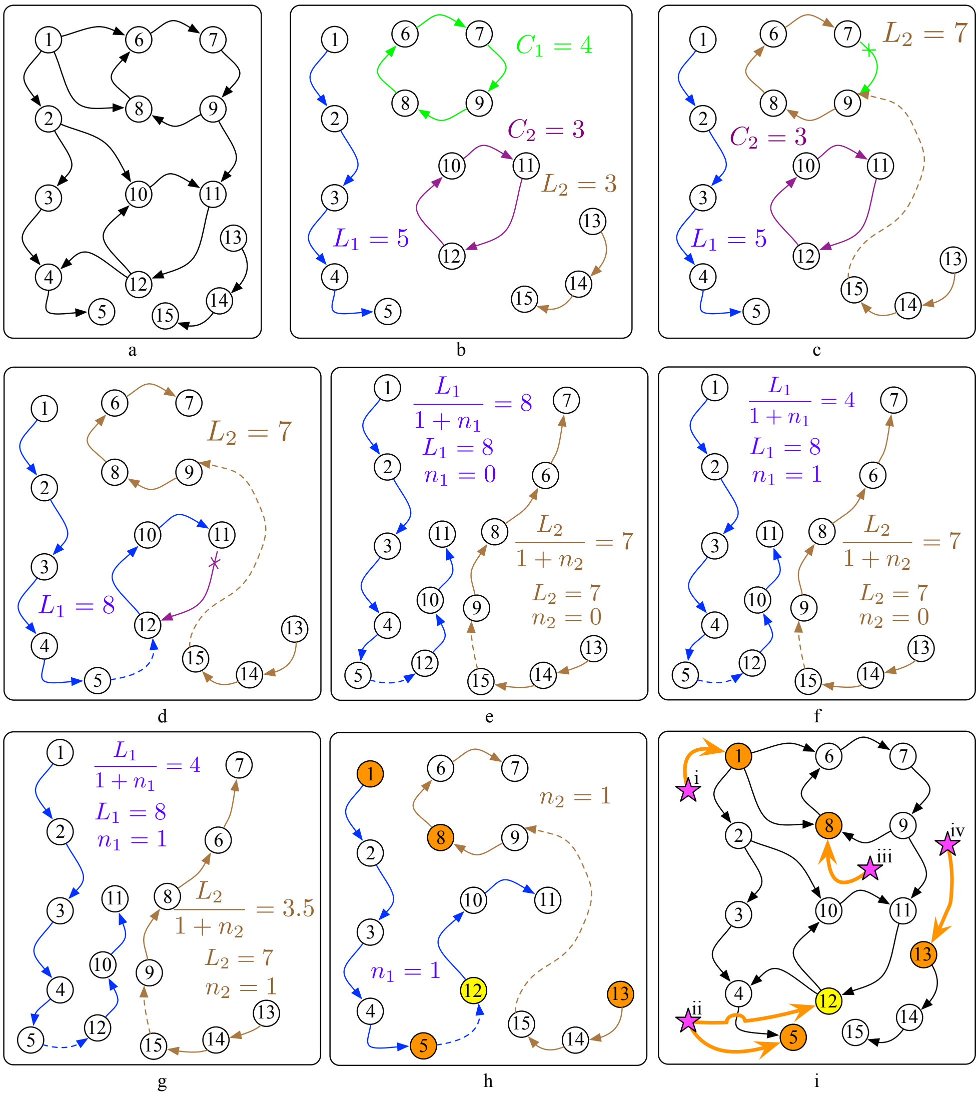

Figure S18: **An example of MLCP algorithm**. (a) The origin topology structure of the network. (b) After the LM algorithm, only matches are left. Thus the network is decomposed into four segments, which include blue and brown DCPs, and green and purple CCPs. (c) At the 1st step, green CCP is selected to be broken. Edge ($7 \to 9$) is removed. A directed dashed arrow from node 15 to node 9 is added and C_1 becomes the tail part of L_2. (d) The purple CCP is selected to be broken. Edge ($11 \to 12$) is removed. Node 5 is assumed to be connected to Node 12. (e) At the beginning of the 2nd step, $n_1 = 0$ and $n_2 = 0$. (f) Because $\frac{L_1}{1+n_1} = 8 > \frac{L_2}{1+n_2} = 7$, the first extra external control input is connected to L_1. Then $n_1 = 1$ and $\frac{L_1}{1+n_1} = 4$. (g) Because $\frac{L_1}{1+n_1} = 4 < \frac{L_2}{1+n_2} = 7$, the second extra external control input is connected to L_2. Then $n_2 = 1$ and $\frac{L_2}{1+n_2} = 3.5$. (h) Node 1, node 5, node 8 and node 13 are set as independent control nodes which are colored in orange. Node 12 is set as a dependent control node colored in yellow. (i) Back to the origin topology of the network, external control inputs are denoted as carmine five-pointed stars.

DCPs and two CCPs. The length of the blue DCP is 5, and the length of the brown DCP is 3. The length of the green CCP is 4, and the length of the purple CCP is 3.

Now we use the MLCP algorithm to locate the external control inputs. In the first step, we randomly select one CCP. In this example as shown in Figure S18(c), we choose the green CCP denoted as C_1. Break it by randomly removing one of its edges. In this example, edge $(7 \to 9)$ is removed. Then imagine it is connected to the end node of a randomly selected DCP. In this example, brown DCP is selected. An directed edge is added from the end node of the brown path to the origin of broken circle (i.e., green path). The added edge is denoted as a directed dashed line. Now the green CCP becomes the tail part of the brown DCP, which increases L_2 by C_2. Thus now $L_2 = 4 + 3 = 7$.

There is still one CCP left, i.e., the purple CCP as shown in Figure S18(d). Similarly as before, we break it by randomly removing one edge of it, say, edge $(11 \to 12)$, and then add a directed dashed from node 5 to node 12. Now $L_1 = 5+3 = 8$. As there is no CCP left, the MLCP algorithm goes to the second step.

At this step, we allocate the extra external control inputs. At the beginning, the blue DCP has $n_1 = 0$ extra external control inputs, and the brown DCP has $n_2 = 0$. As shown in Figure S18(e), the blue DCP has a larger $\frac{L_i}{1+n_i}$. Thus the first extra external control input is connected to the blue DCP.

Afterwards, $n_1 = 1$ and n_2 is still zero, and the brown DCP now has a larger $\frac{L_i}{1+n_i}$ as shown in Figure S18(f). Therefore, the second external control input is connected to the brown DCP. Now $n_1 = 1$ and $n_2 = 1$, as shown in Figure S18(g).

The third step is to distribute the external control inputs as uniformly as possible in each DCP. For the blue DCP, it has two external control inputs. The first one has to be connected to the origin of the DCP to ensure the structural controllability. The second one is set on node 5 to divide DCP as evenly as possible. Because node 12, node 10 and node 11 which originally consist the purple CCP all lack independent control inputs directly setting on them, we randomly choose one node from the purple CCP (in this example, node 12 is selected which is colored in yellow as shown in Figure S18(h)) and connect the nearest external control input in the blue DCP, which is the control input set on node 5, to node 12.

As for the brown DCP, similarly as before we set the origin and node 8 as the independent control nodes. The result is shown in Figure S18(h). All the independent control nodes are colored in orange.

At last, we allocate the four external control inputs into the network as shown in Figure S18(i). External control inputs are denoted as carmine five-pointed stars.

Table S5: **Network Experiment Results.** N_D^{LM} represents the number of driver nodes found by LM, with which the network becomes controllable. m_0 represents the number of extra control inputs added into network. N.A denotes "not available" because PGM and OPGM are not applicable to large size networks due to the computational complexity issues. The elements in the input matrix B^* are allowed to be real values for PGM and OPGM but only binary values for MLCP and RAM.

Network	Nodes	Edges	N_D	N_D^{LM}	m_0	Energy Cost			
						PGM	OPGM	MLCP	RAM
circuit-s208	122	189	29	29	48	3.42E02	3.39E02	9.39E02	6.48E09
circuit-s420	252	399	59	59	74	1.32E03	1.22E03	1.41E04	6.61E10
circuit-s838	512	819	119	119	178	2.57E03	1.94E03	4.75E03	2.86E10
Maspalomas	30	87	9	9	6	1.55E02	1.65E02	3.52E03	1.05E07
Rhode	25	58	8	8	7	0.83E02	1.00E02	4.20E02	1.39E05
p2p-Gnutella04	10876	39994	4640	4700	3940	N.A	N.A	4.64E04	3.29E07
p2p-Gnutella25	22687	54705	16478	16478	2000	N.A	N.A	1.89E05	3.06E08
E-R Network	100	299	19	19	31	3.95E02	3.92E02	3.11E03	1.98E05
	500	1492	87	87	176	1.64E03	1.80E03	9.0E03	2.62E07
	1000	3033	182	182	528	N.A	N.A	5.15E03	2.76E05
B-A Network	100	341	21	21	20	1.75E03	1.39E03	2.39E04	6.59E07
	500	1548	144	144	100	2.81E03	2.56E03	3.35E04	1.48E07
	1000	3039	263	263	355	N.A	N.A	9.61E03	5.53E06
physician-discuss	231	498	85	85	100	3.80E02	3.18E02	9.22E02	4.50E04
physician-friend	228	506	52	52	66	1.01E03	9.40E02	2.37E04	1.3612E06
corp-own	7253	6713	5777	5777	800	N.A	N.A	1.65E04	3.26E06

Table S6: **Dense Network Experiment Results.** N_D^{LM} represents the number of driver nodes found by LM, with which the network becomes controllable. m_0 represents the extra number of control inputs added into network.

Network	Nodes	Edges	N_D	N_D^{LM}	m_0	Energy Cost			
						PGM	OPGM	MLCP	RAM
us	1574	28236	581	588	493	N.A	N.A	8.49E01	1.36E02
CrystalC	24	125	10	10	7	0.48E02	0.40E02	3.71E03	4.48E03
E-R Network	200	1534	2	2	80	1.00E03	9.73E02	1.49E04	1.73E04
B-A Network	200	1531	2	2	80	1.12E03	1.07E03	1.93E04	2.16E04
celegans	297	2345	49	49	121	7.87E02	6.84E02	7.33E03	1.55E04
cons-frequency	46	879	2	2	15	0.05E02	0.05E02	1.23E02	1.27E02
Wiki-Vote	7115	103689	4736	4736	1000	N.A	N.A	3.50E03	9.40E03

4.3 Performance of Minimizing Longest Control Path

MLCP is applied to a few synthetic networks including the ER network with Poisson nodal-degree distribution [24], the BA network with power-law nodal-degree distribution [25] [26], and a large number of real-life networks. To evaluate the performance of the MLCP method, we let it be compared with a method with random connections between controllers and network nodes. In order to generate a random solution that ensures network controllability, we first apply the LM algorithm to find one set of driver nodes, which is very close to the optimal solutions found by MM as shown in Table S2 and S3, Section 2. Then we randomly choose m_0 additional nodes in the network to construct the control node set. This simple method is termed as *Random Allocation Method* (RAM) in the main paper. For MLCP, similarly we use the LM algorithm to generate the driver node set and then use MLCP to allocate the m_0 additional control nodes. We calculate the control costs of network corresponding to MLCP and RAM solutions, respectively.

The results on both synthetic networks and real-life networks are summarized in Tables S5-S6. Data of the topologies of the real-life networks is freely available in the cited references.

From Table S5, we observe that MLCP performs comparably against OPGM though each control node is restricted to be connected to a single external input, and it steadily outperforms RAM by an average of about four orders of magnitude. From Table S6, however, the observations are rather different. As the listed networks are relatively dense networks, the control costs obtained by MLCP are averagely only about half less than those obtained by RAM. Overall, similar conclusions have held in all our extensive simulations: MLCP significantly outperforms RAM in low-degree networks while the two methods perform almost indistinguishably as the networks become dense.

4.4 Experiments and Analysis for Figure 4(c) in the Main Paper

To demonstrate that MLCP performs much better than RAM in sparse networks, while the performances of the two methods become less distinguishable as the mean degree of the network becomes higher, we illustrate on one case in an evolving network with an increasing mean nodal degree. The results are presented in Figure 4(c) in the main paper. The network evolution and the simulation testing method are summarized as follows: (1) generate an ER network with an mean degree of about $\mu = 1$; (2) randomly choose a pair of nodes and add one directed edge between them; (3) using MLCP and RAM to assign $M = N_D^{LM} + m_0$ external control inputs into the network; and (4) calculate the control costs of the network corresponding to MLCP and RAM solutions, respectively. Repeat the steps (2)-(4) until the mean degree equals 14.

For a sparse network with a low mean degree, the number of driver nodes has to be relatively high

to ensure structural controllability, which leads to a relatively low control cost. As the mean degree increases, the number of driver nodes needed decreases and the average/maximum length of control paths gradually increases, which interestingly drives up the control cost as we set m_0 a constant value of 80 in this set of experiments. This explains the increasing control cost with the mean degree that can be observed in Figure 4(c). When the mean degree of the network is further increased, the number of driver nodes N_D^{LM} decreases insignificantly but adding edges makes the average/maximum length of control paths become shorter, which reduces the control cost. Overall, the conclusion is that either having a larger number of controllers or a higher network density helps shorten the average/maximum control path length, and consequently lowers network control cost. As to the comparisons between MLCP and RAM, we see that MLCP outperforms RAM by up to eight orders of magnitude in sparse network, while the two methods perform comparably when the mean in-/out-degree of the network is larger than 6.

References

[1] Lin, C. Structural controllability. *IEEE Trans. Automat. Contr.* **19**, 201-208 (1974).

[2] Hartshorne, R. Algebraic Geometry. *Springer-Verlag*, 1977.

[3] Hopcroft, J. E. & Karp, R. M. An $n^{5/2}$ algorithm for maximum matchings in bipartite graphs. *SIAM J. Comput.* **2**, 225-231, 1973.

[4] Israeli, A. & Itai, A. A fast and simple randomized parallel algorithm for maximal matching. *Information Processing Letters* **22**, 77-80, 1986.

[5] Schieber, B. & Moran, S. Parallel algorithms for maximum bipartite matchings and maximum 0-1 flows. *Journal of Parallel and Distributed Computing* **6**, 20-38, 1989.

[6] Wu, M. M. & Loui, M. C. An encient distributed algorithm for maximum matching in general graphs. *Algorithmica* **5**, 383-406, 1990.

[7] Hoepman, J. H. Simple distributed weighted matchings. arXiv preprint cs/0410047, 2004.

[8] Lotker, Z., Patt-Shamir, B. & Rosen, A. Distributed approximate matching. *SIAM Journal on Computing* **39**, 445-460, 2009.

[9] Mansour, Y. & Vardi, S. A local computation approximation scheme to maximum matching. *Approximation, Randomization, and Combinatorial Optimization. Algorithms and Techniques*, Springer, 260-273, 2013.

[10] Chen, S., Li, X. & Zhou, X. Y. Stochastic linear quadratic regulators with indefinite control weight costs. *SIAM Journal on Control and Optimization* **36**, 1685-1702, 1998.

[11] Bemporad, A. et al. The explicit linear quadratic regulator for constrained systems. *Automatica* **38**, 3-20, 2002.

[12] Anderson, B.D.O. & Moore, J.B. Optimal control: linear quadratic methods. *Courier Corporation*,2007.

[13] Li, D., Qian, F. & Fu, P. Optimal nominal dual control for discrete-time linear-quadratic Gaussian problems with unknown parameters. *Automatica* **44**, 119-127, 2008.

[14] Selesnick, I. W. The estimation of Laplace random vectors in additive white Gaussian noise. *IEEE Transactions on Signal Processing* **56**, 3482-3496, 2008.

[15] Jalili, M., Sichani, O. A. & Yu, X. Optimal pinning controllability of complex networks: Dependence on network structure. *Phys. Rev. E* **91**, 012803, 2015.

[16] Pham, Q. C. & Slotine, J. J. Stable concurrent synchronization in dynamic system networks. *Neural Networks* **20**, 62-77, 2007.

[17] Yu, W., Chen, G. & Lu, J. On pinning synchronization of complex dynamical networks. *Automatica* **45**, 429-435 (2009).

[18] Lin, F., Fardad, M. & Jovanovic, M. R. Algorithms for leader selection in stochastically forced consensus networks. *IEEE Trans. Automat. Contr.* **59**, 1789-1802 (2014).

[19] Liu, Y. Y., Slotine, J. & Barabasi, A. Controllability of complex networks. *Nature* **473**, 167-173, 2011.

[20] Harsanyi, J. C. Games with Incomplete Information Played by Bayesian Players. *Management Science* **14**, 159-183, 1967.

[21] Wilson, E. B. &Hilferty, M. M. The distribution of chi-square. *Proc. Natl. Acad. Sci.* **17**, 684-688, 1931.

[22] Muraleedharan, G. &Soares, C. G. Characteristic and moment generating functions of generalised Pareto and Weibull distributions. *Journal of Scientific Research and Reports* **3**, 1861-1874, 2014.

[23] Banneheka, B. & Ekanayake, G. A new point estimator for the median of gamma distribution. *Vidyodaya Journal of Science* **14**, 95-103, 2009.

[24] Erdos, P. & Renyi, A. On the Eevolution of random graphs. *Publ. Math. Inst. Hungar. Acad. Sci.* 5, 17-61, 1960.

[25] Albert, R. & Barabasi A. Statistical mechanics of complex networks, *Rev. Mod. Phys.* **74**, 47-97, 2000.

[26] Newman M. E. J. Power laws, Pareto distributions and Zipf's law. *Contemp. Phys.* **46**, 323-351, 2013.

[27] Opsahl, T., Agneessens, F. & Skvoretz, J. Node centrality in weighted networks: generalizing degree and shortest paths. *Social Networks* **32**, 245-251, 2010.

[28] Marcelino, J. & Kaiser, M. Critical paths in a metapopulation model of H1N1: efficiently delaying influenza spreading through flight cancellation. *PLoS Currents* **4**, 2012.

[29] Adamic, L. A. & Glance, N. The political blogosphere and the 2004 US election: divided they blog. *Proc. Int. Workshop on Link Discovery, ACM* **2005**, 26-43, 2005.

[30] Leskovec, J. et al. Community structure in large networks: natural cluster sizes and the absence of large well-defined clusters. *Internet Mathematics* **6**, 29-123, 2009.

[31] Albert, R., Jeong, H. & Barabasi, A. L. Internet: diameter of the world-wide web. *Nature* **401**, 130-131, 1999.

[32] Milo, R. et al. Superfamilies of evolved and designed networks. *Science* **303**, 1538-1542, 2004.

[33] Christian, R. R. & Luczkovich, J. J. Organizing and understanding a winters seagrass foodweb network through effective trophic levels. *Ecol. Model.* **117**, 99-124, 1999.

[34] Monaco, M. E. & Ulanowicz, R. E. Comparative ecosystem trophic structure of three US mid-Atlantic estuaries. *Mar. Ecol. Prog. Ser.* **161**, 239-254, 1997.

[35] Almunia, J. et al. Benthic-pelagic switching in a coastal subtropical lagoon. *Estuar. Coast. Shelf S.* **49**, 363-384, 1999.

[36] Baird, D., Luczkovich, J. & Christian, R. R. Assessment of spatial and temporal variability in ecosystem attributes of the St Marks National Wildlife Refuge, Apalachee Bay, Florida. *Estuar. Coast. Shelf S.* **47**, 329-349, 1998.

[37] Baird, D. & Ulanowicz, R. E. The seasonal dynamics of the Chesapeake Bay ecosystem. *Ecol. Monogr.* **59**, 329-364, 1989.

[38] Leskovec, J., Kleinberg, J. & Faloutsos, C. Graph evolution: densification and shrinking diameters. *ACM Trans. Knowl. Discov. D.* **1**, 2, 2007.

[39] Ripeanu, M. & Foster, I. Mapping the gnutella network: macroscopic properties of large scale peer-to-peer systems. *Peer-to-Peer Systems. Springer Berlin Heidelberg* **2429**, 85-93, 2002.

[40] Leskovec, J., Huttenlocher, D. & Kleinberg, J. Signed networks in social media. *Proc. SIGCHI Conference on Human Factors in Computing Systems* **2010**, 1361-1370, 2010.

[41] Burt, R. S. Social contagion and innovation: cohesion versus structural equivalence. *American Journal of Sociology* **92**, 1287-1335, 1987.

[42] Leskovec, J., Huttenlocher, D. & Kleinberg, J. Predicting positive and negative links in online social networks. *Proc. Int. Conference on World Wide Web* **2010**, 641-650, 2010.

[43] Watts D. J. & Strogatz, S. H. Collective dynamics of small-worldnetworks. *Nature* **393**, 440-442, 1998.

[44] Bakker, R. Wachtler, T. & Diesmann, M. CoCoMac 2.0 and the future of tract-tracing databases. *Frontiers in neuroinformatics* **6**, 30, 2012.

[45] Kaiser, M. & Hilgetag, C. C. Nonoptimal component placement, but short processing paths, due to long-distance projections in neural systems. *PLos Comput. Biol.* **2**, e95, 2006.

[46] Young, M. P. The organization of neural systems in the primate cerebral cortex. *Proc. Roy. Soc. Lond. B Bio.* **252**, 13C18, 1993.

[47] Takac, L. & Zabovsky, M. Data Analysis in Public Social Networks. *Int. Scientific Conference and International Workshop Present Day Trends of Innovations* **2012**, 1-6, 2012.

[48] Leskovec, J., Adamic, L. A. & Huberman, B. A. The dynamics of viral marketing. *ACM Trans. Web* **1**, 5, 2007.

[49] Opsahl, T. & Panzarasa, P. Clustering in weighted networks. *Social Networks* **31**, 155-163, 2009.

[50] Norlen, K. et al. EVA: extraction, visualization and analysis of the telecommunications and mediaownership network. *Proc. Int. Telecommunications Society Biennial Conference* **2002** , 1-27, 2002.

[51] Milo, R. et al. Network Motifs: Simple Building Blocks of Complex Networks. *Science* **298**, 824-827, 2002.

[52] Li, G. et al. Minimum cost control of complex networks. *New Journal of Physics* **18**, 013012, 2016.

[53] Rugh, W. J. Linear system theory. *Prentice-Hall, USA*, 1996.

[54] Klipp, E. et al. Systems Biology: a textbook. *Wiley-VCH, Germany*, 2009

[55] Dattorro, J. Convex optimization & Euclidean distance geometry. *Meboo publishing USA*, 2010.
